# Repeat 1 of TAL effectors affects target specificity for the base at position zero

**DOI:** 10.1093/nar/gku341

**Published:** 2014-05-03

**Authors:** Tom Schreiber, Ulla Bonas

**Affiliations:** Department of Genetics, Martin Luther University, Weinbergweg 10, 06120 Halle (Saale), Germany

## Abstract

AvrBs3, the founding member of the *Xanthomonas* transcription-activator-like effectors (TALEs), is translocated into the plant cell where it localizes to the nucleus and acts as transcription factor. The DNA-binding domain of AvrBs3 consists of 17.5 nearly-identical 34 amino acid-repeats. Each repeat specifies binding to one base in the target DNA via amino acid residues 12 and 13 termed repeat variable diresidue (RVD). Natural target sequences of TALEs are generally preceded by a thymine (T_0_), which is coordinated by a tryptophan residue (W232) in a degenerated repeat upstream of the canonical repeats. To investigate the necessity of T_0_ and the conserved tryptophan for AvrBs3-mediated gene activation we tested TALE mutant derivatives on target sequences preceded by all possible four bases. In addition, we performed domain swaps with TalC from a rice pathogenic *Xanthomonas* because TalC lacks the tryptophan residue, and the TalC target sequence is preceded by cytosine. We show that T_0_ works best and that T_0_ specificity depends on the repeat number and overall RVD-composition. T_0_ and W232 appear to be particularly important if the RVD of the first repeat is HD (‘rep1 effect’). Our findings provide novel insights into the mechanism of T_0_ recognition by TALE proteins and are important for TALE-based biotechnological applications.

## INTRODUCTION

**Figure 1.**
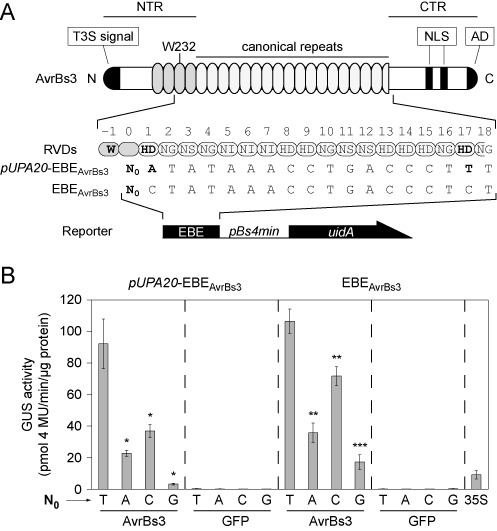
Imperfect target sequences of AvrBs3 increase the importance of T_0_. (**A**) Schematic presentation of AvrBs3 and reporter constructs. Repeats are indicated by ovals [gray: degenerated repeats in the N-terminal region (NTR)]. T3S signal: T3S secretion signal; NLS: nuclear localization signal; AD: activation domain; EBE: effector binding element (upper strand); N_0_: position zero of EBE. Bold letters refer to mismatches in the EBE_AvrBs3_. EBEs were fused to the *Bs4* minimal promoter (*pBs4min*) driving expression of the β-glucuronidase (GUS) reporter gene *uidA*. (**B**) Quantification of AvrBs3 activities for different EBEs. GUS activities were determined 3 days after *Agrobacterium*-mediated delivery of effector- and reporter-constructs into leaves of *Nicotiana benthamiana* (see ‘Materials and Methods’ section). Asterisks indicate a significant difference in activity of the same TALE-derivative tested with EBE-T_0_ (Student's t-test; *P-value ≤ 0.05; **P-value ≤ 0.01; ***P-value ≤ 0.001). Experiments were performed three times with similar results.

Transcription activator-like effectors (TALEs) are bacterial type III effector proteins in plant-pathogenic *Xanthomonas* spp., which act as transcription factors in the plant cell (1). AvrBs3, the founding member of the highly conserved TALE family, was isolated from the pepper and tomato pathogen *X. campestris* pv. *vesicatoria* (*Xcv*) (2). We previously showed that AvrBs3 is translocated into the plant cell via the type III secretion system, localizes to the nucleus and activates *UPA* (upregulated by AvrBs3) genes, including the cell size regulator *UPA20* and the resistance gene *Bs3* in pepper (3–5). TALE proteins are characterized by three conserved domains: an N-terminal region (NTR) which harbors the type III secretion and translocation signal, a central repeat region of variable length that has deoxyribonucleic acid (DNA)-binding activity and a C-terminal region (CTR) that contains nuclear localization signals (NLSs) and an acidic activation domain (AD) (1) (Figure 1A). The repeat region determines the specificity of a given TALE and represents a novel type of DNA-binding domain (4,5). The archetypal TALE, AvrBs3, contains 17.5 nearly-identical tandem repeats of 34 amino acids (aa) which differ mainly at positions 12 and 13, termed repeat variable diresidue (RVD). Experimental and computer-based analyzes revealed a ‘one repeat to one base pair’ recognition mode of TALEs in which one RVD specifies binding to one nucleotide in the target sequence. The most common RVDs are HD, NI, NG and NN, which specifically bind cytosine, adenine, thymine and guanine/adenine, respectively (6,7). Crystal structures of TALEs with and without DNA provided insights into the structural basis for the TALE–DNA interaction (8–11). The repeat region forms a superhelical structure that, if bound to double-stranded DNA, is wrapped around the DNA helix tracking along the sense strand. Comparison of DNA-free and DNA-bound TALEs revealed a conformational change of the protein that is compressed upon DNA-binding (8). Each repeat contains two α-helices connected by a loop which exposes residue 13 (RVD-loop). While only amino acid 13 mediates the specific contact to the matching base, amino acid 12 has a structural function by contacting the alanine residue (position 8) and the isoleucine residue (position 9) in the first helix of the same repeat which stabilizes the RVD-loop (8,10,12). The phosphate group of each nucleotide is coordinated by the residues glycine (positions 14 and 15), lysine (position 16) and glutamine (position 17) of the following repeat (*oxyanion clip*) fixing residue 13 and facilitating RVD-base specificity by a combination of positive recognition and negative discrimination (8,12).

In nature, the RVD-defined target sequences (effector binding elements; EBEs) are typically preceded by a thymine at position zero (T_0_), which was shown to be important for full TALE function (6,13,14). To our knowledge, TalC from the African *X. oryzae* pv*. oryzae* (*Xoo*) strain BAI3 is the only TALE for which a natural target sequence is preceded by a cytosine (C_0_) in the promoter of the susceptibility gene *OsSweet14* (*pOsSweet14*) (15). Structure analyzes revealed that the TALE DNA-binding domain is extended by four degenerated repeats in the NTR, termed repeat −3, −2, −1 and 0 (9–11). Although a non-canonical ‘repeat zero’ was predicted to coordinate binding to T_0_ (6) the initial T is coordinated by repeat −1 (10). Intriguingly, repeat −1 forms an α-helices-connecting loop comparable to the RVD-loop of canonical repeats. Repeat −1 contains a tryptophan residue (W232 in AvrBs3), which is believed to coordinate T_0_ by van der Waals interactions (9,10). By contrast, Stella *et al*. (11) who provide a 3D structure of DNA-bound AvrBs3 discuss that R266 in AvrBs3 contacts T_0_. Notably, both the tryptophan and arginine residues are conserved in TALE proteins. One exception is TalC which harbors a cysteine instead of a tryptophan (15). Recently, TALE homologs from *Ralstonia solanacearum* (RTLs*-Ralstonia* TALE-like) were described to function similarly to TALEs from *Xanthomonas*, however, RTLs need G_0_ in the corresponding EBEs (16). The NTR of RTLs differs from TALEs, but structure prediction suggests similar folding and that RTLs coordinate G_0_ with an arginine (16,17).

The discovery of the TALE recognition mode [‘TAL-code’; (6)] allows the target prediction of natural TAL effectors as well as the generation of new DNA-binding domains with any desired DNA-binding specificity (18). Besides designing TALEs for gene activation different executor domains can be fused to the DNA-binding domain, e.g. a FokI nuclease. TALEs therefore became a powerful tool for biotechnological applications such as genome editing (18). As the need for T_0_ limits DNA-targeting by artificial (designer) TALEs (dTALEs) we wondered whether the specificity for position zero can be changed and how important the tryptophan at position 232 (W232) is for AvrBs3 activity. We, therefore, analyzed AvrBs3 derivatives carrying different amino acid substitutions at position 232 in the context of different RVDs in repeat 1 with respect to specificity for the initial nucleotide (N_0_). Here, we demonstrate that T_0_ specificity depends on the number of repeats and the RVD composition, and that RVD1 affects T_0_ specificity.

## MATERIALS AND METHODS

### Bacterial and plant growth conditions

*Escherichia coli* strains were grown at 37°C in lysogenic broth media (LB; tryptone 10 g/l, yeast extract 5 g/l, 170 mM NaCl, pH 7.0) with selective antibiotics. *Agrobacterium tumefaciens* strain GV3101 was grown at 30°C in yeast extract broth media (YEB; beef extract 5 g/l, bacto yeast extract 1 g/l, bacto peptone 5 g/l, 15 mM sucrose, 1 M MgSO_4_, pH 7.2) with selective antibiotics. *Nicotiana benthamiana* plants were grown in the greenhouse (day and night temperatures of 23°C and 19°C, respectively) with 16 h light and 40–60% humidity.

### Generation of reporter and effector constructs

An entry clone containing the *UPA20* promoter target sequence of AvrBs3 (*pUPA20*-EBE_AvrBs3_) in front of the tomato *Bs4* minimal promoter (*pBs4min*) (19) was used as template to generate *pUPA20*-EBE_AvrBs3_ and the optimized EBE (EBE_AvrBs3_) with varying nucleotides at positions N_0_ and N_1_ (Supporting information). Mutations were introduced by PCR using oligonucleotides TS23 and TS24-TS42 (Supporting information, Table S1). Entry clones with the EBEs of ARTrep18-1, ARTrep18-2 and ARTrep18-3 in front of *pBs4min* were used as a template to generate mutations at position 0 using oligonucleotides TS23 and TS43–TS48 (Supporting information, Table S1). Inserts of entry clones were recombined into pGWB3 (20) (GATEWAY^®^ LR Clonase^®^ II Enzyme mix; Life Technologies) leading to AvrBs3-inducible β-glucuronidase (GUS) reporter constructs. The coding sequence (CDS) of *avrBs3* (accession number: X16130) was cloned by ‘Golden Gate’ cloning as reported (21). The *avrBs3* CDS was divided into three modules (NTR, repeat region and CTR; Supporting information) which were flanked by BsaI sites and cloned into pJET (Thermo SCIENTIFIC). This allows assembly of single modules into a compatible destination vector (21). Point mutations in the *avrBs3* NTR were introduced by PCR using oligonucleotides TS1 + TS2 − TS18; TS19 + TS20; TS21 + TS22) (Supporting information, Table S1). Changes in AvrBs3 RVD-composition and the generation of artificial repeat regions were accomplished using a TALE-repeat library based on *hax3* (Supporting information) (21). The *avrBs3* sub-modules and repeat regions were assembled as N-terminal *c-Myc* fusions into the binary vector pGGA8 (S. Thieme unpublished; Supporting information) allowing expression of *avrBs3* or *artrep18* constructs under control of the constitutive cauliflower mosaic virus *35S* promoter (effector construct). *A. tumefaciens* strain GV3101 was transformed with reporter or effector constructs by electroporation.

### AvrBs3-activity assay

Transient GUS reporter assays were performed as described (4). *Agrobacterium* carrying an effector and reporter construct, respectively, was resuspended in *Agrobacterium* infiltration media (10 mM MES, 10 mM MgCl_2_, 150 μM acetosyringone) to an optical density of 0.8 and mixed in a 1:1 ratio. *Agrobacterium* mixtures were inoculated into leaves of four to seven weeks old *N. benthamiana* plants using a needleless syringe. Two to three days post inoculation (dpi) two leaf discs (diameter 0.9 cm) of three plants were harvested and used for quantitative GUS activity assays (4). Green fluorescent protein (GFP) and *35S:GUS* (35S) served as negative and positive controls, respectively. Error bars are based on the standard deviation from three technical replicates. Experiments were performed three times with similar results.

### Analysis of protein expression

Two to three days post infection (dpi) three leaf discs were harvested and ground by TissueLyser (Qiagen). Protein extracts were mixed with 100 μl 4xLaemmli (250 mM Tris–HCl (pH 6.8), 8% sodium dodecyl sulphate (SDS), 40% glycerol, 10% β-mercaptoethanol) and boiled for 5 min. Protein samples were separated by 8% SDS-polyacrylamide gel electrophoresis and transferred to nitrocellulose. C-Myc tagged proteins were detected using a polyclonal c-Myc-specific antibody (Santa Cruz). ECL™Anti-Rabbit IgG (GE Healthcare) was used for detection by enhanced chemiluminescence.

## RESULTS

### Imperfect target sequences increase the importance of T_0_

Permutation of the AvrBs3-targeted *UPA* box in the *Bs3* promoter revealed that a thymine at position zero (T_0_) is essential for the AvrBs3-induced hypersensitive response (13). However, *Bs3* promoter activation was not quantified. Interestingly, the *UPA* box consensus contains a mismatch at position 1, which is bound by the first RVD (HD1), i.e. adenine instead of cytosine (19). Here, we investigated the effects of mismatches in the AvrBs3 target box and quantified promoter activation in dependency of the nucleotide at position zero (N_0_). For this, we used the established reporter system consisting of the *Bs4* minimal promoter (*pBs4min*) preceded by the AvrBs3-effector binding element (EBE_AvrBs3_) driving expression of GUS (6). AvrBs3 and GFP (negative control) were expressed as N-terminal c-Myc fusions under control of the strong and constitutive *35S* promoter. Both, the reporter and effector (or GFP) expression constructs were delivered by *A. tumefaciens* into leaves of *N. benthamiana* (Figure 1A). We generated four reporter constructs differing at N_0_ containing (i) the *UPA20*-derived EBE_AvrBs3_ (*UPA20*-EBE_AvrBs3_) and (ii) the optimized, RVD-defined EBE_AvrBs3_, respectively. As shown in Figure 1, we confirmed the importance of T_0_ for activation by AvrBs3 with the hierarchy T_0_ > C_0_ > A_0_ > G_0_. The comparison between the different EBEs suggests that in case of imperfect target sequences the importance of T_0_ increases and that all nucleotides at position zero work better in the optimal EBE_AvrBs3_ (Figure 1B). As shown in Supplementary Figure S1, AvrBs3 and GFP were stably expressed. To exclude side effects due to mismatches we used the optimal EBE_AvrBs3_ in all following experiments.

### Analysis of AvrBs3 tryptophan (W232) mutants

**Figure 2.**
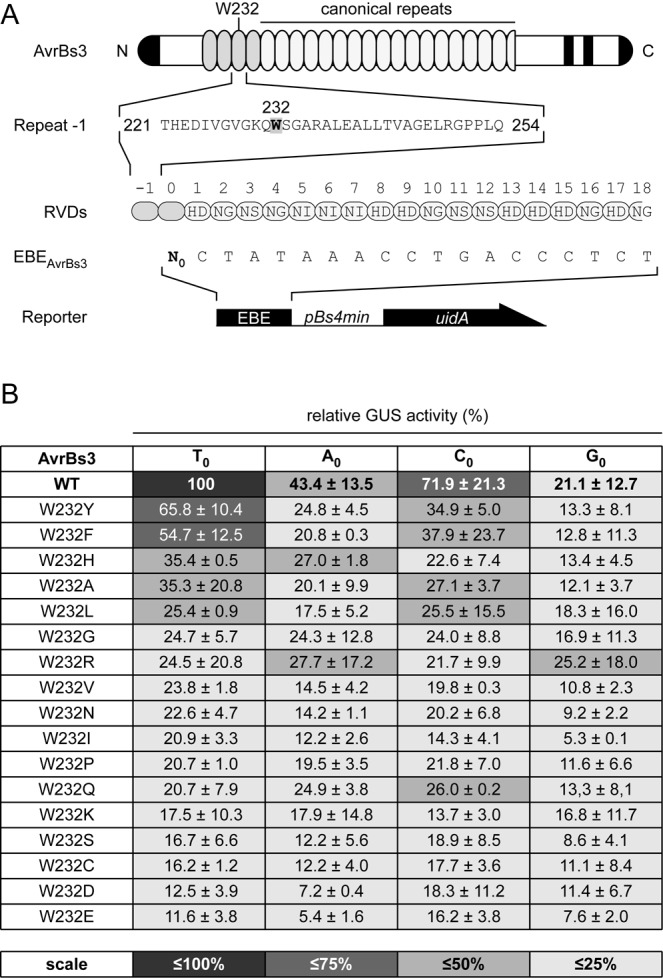
W232 is necessary for full AvrBs3 activity. (**A**) Schematic presentation of AvrBs3 and reporter constructs. The amino acid sequence of repeat −1 is given; residue W232 is highlighted. (**B**) Relative GUS activity (%) induced by AvrBs3 and W232-mutants. Reporter constructs differed at position N_0_. AvrBs3(WT) activity with EBE(T_0_) was set to 100%. Standard deviation is based on the mean of three independent experiments. Color scale: GUS activities smaller than 100%.

Structural data revealed that a tryptophan residue located in the ‘RVD-loop’ of repeat −1 is the most proximal amino acid to T_0_ in the target DNA. The tryptophan is believed to interact with the base by van der Waals forces (10). Both the tryptophan residue and T_0_ are highly conserved in natural TALEs and target sequences, respectively (18). To investigate the importance of tryptophan at position 232 (W232) in AvrBs3 and to identify amino acids that broaden or change target specificity for N_0_ we generated *avrBs3* mutant derivatives. The activity of AvrBs3 and derivatives was determined using the GUS reporter system containing the optimal EBE_AvrBs3_ (Figure 2A). Figure 2B shows that most amino acid substitutions in AvrBs3 led to drastically reduced activity. However, substitutions of W232 by the aromatic amino acids tyrosine (W232Y) and phenylalanine (W232F) retained the highest activity (∼70 and 50%) compared to the wild-type (WT) protein and, like WT AvrBs3, worked best with T_0_ (Figure 2B). Expression of all proteins was confirmed by immunoblot (Supplementary Figure S2). Together, these results confirm the crucial importance of W232 in AvrBs3. There were no AvrBs3 derivatives with single substitutions that significantly performed better with any nucleotide at position zero (N_0_) than the WT. Only AvrBs3(W232R) showed slightly increased activity in combination with G_0_.

Recently, a 3D structure of DNA-bound AvrBs3 was described (11). Notably, comparison of the structure of the NTR to previously published structures suggests different residues to be crucial for the coordination of T_0_. In AvrBs3, T_0_ is supposed to be coordinated by arginine 266 (R266) in repeat 0, with the participation of R236 in repeat −1 (Supplementary Figure S3A) (11). We therefore substituted R266 in AvrBs3 by glycine and found slightly reduced activity, but specificity for T_0_ was comparable to WT AvrBs3 (Supplementary Figure S3B). The AvrBs3 derivative R236G displayed only low activity with the T_0_ EBE, possibly due to very low protein expression levels, which was below the detection limit (Supplementary Figure S3B).

### The degenerated repeats cooperate with repeat 1

**Figure 3.**
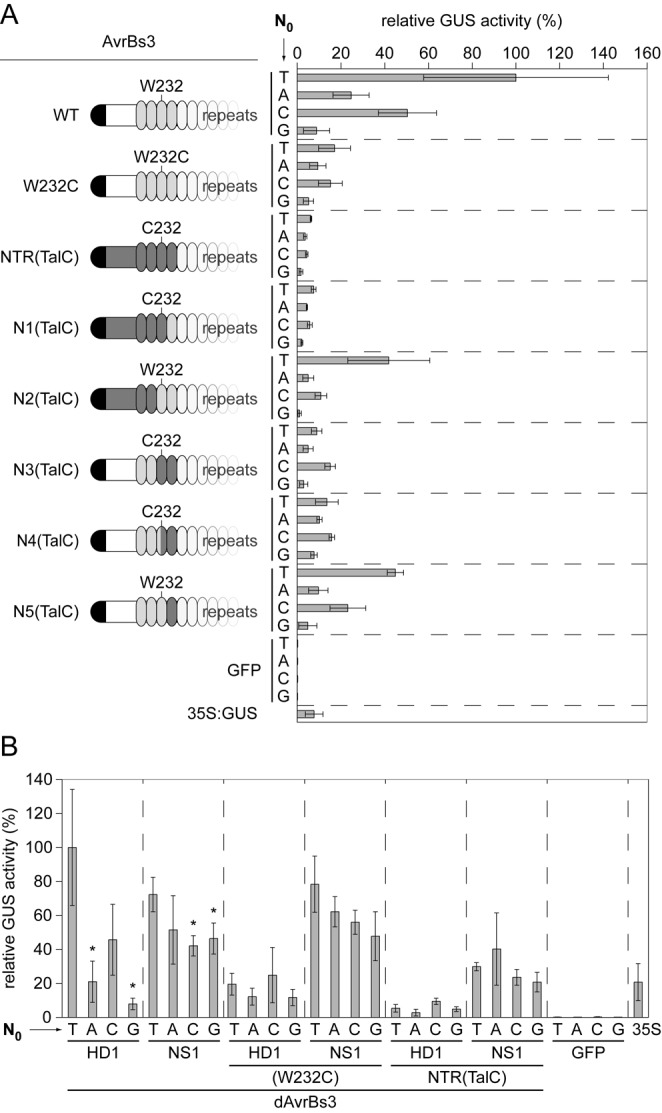
Repeat 1 of AvrBs3 cooperates with degenerated repeats of the NTR. (**A** and **B**) Relative GUS activities (%) induced by AvrBs3 and derivatives 3 days after *Agrobacterium*-mediated delivery of effector- and reporter-constructs into leaves of *Nicotiana benthamiana*. AvrBs3(WT) activity with EBE(T_0_) was set to 100%. Asterisks indicate a significant difference in activity of the same TALE-derivative tested with EBE-T_0_ (Student's t-test; *P-value ≤ 0.05; **P-value ≤ 0.01; ***P-value ≤ 0.001). Experiments were performed three times with similar results.

The tryptophan (W232) that coordinates TALE contact to the base at position N_0_ is not conserved in TalC from the rice pathogen *X. oryzae* pv. *oryzae* (15). Instead, TalC contains a cysteine residue, which when introduced into AvrBs3 led to low activity [AvrBs3(W232C); Figure 2B]. Notably, the natural target box of TalC starts with C_0_ (15). TalC harbors additional substitutions and a deletion of 23 aa in the NTR (Supplementary Figure S4A). To test whether the NTR of TalC confers a preference for an EBE with C_0_ we compared the activities of AvrBs3, AvrBs3(W232C) and chimeras between TalC and AvrBs3 (Figure 3A). As targets we used the same four different EBE_AvrBs3_-reporters as in Figure 2. Surprisingly, the swap of the NTRs resulted in a non-functional AvrBs3 protein. Sequence comparison (Supplementary Figure S4A) revealed an amino acid difference in TalC repeat 0, which according to 3D data (9) is located in an α-helical region that is tightly packed together with the neighboring helix of the canonical repeat 1. We therefore tested chimeras between TalC-NTR and AvrBs3 in which we shortened the fragment contributed by TalC. As shown in Figure 3A, AvrBs3 activity improved when the protein contained only the very NTR of TalC including repeat −2 [AvrBs3-N2(TalC)]. However, AvrBs3 containing only repeat −1 and repeat 0 from TalC displayed very low activity [AvrBs3-N3(TalC)]. Only the exchange of repeat 0 is tolerated (AvrBs3-N5(TalC) but led to reduced AvrBs3 activity (Figure 3A). This confirms our hypothesis that amino acid differences in the helices of repeat 0 and repeat 1 affect protein activity. Furthermore, the results underpin the necessity of W232 for AvrBs3 function.

Next, we reasoned that the degenerated repeats (repeat −3 to 0) might cooperate with repeat 1 which differs between TalC (NS1) and AvrBs3 (HD1) (Supplementary Figure S4B). We therefore generated designer AvrBs3 constructs [dAvrBs3; WT, W232C, NTR(TalC)] differing in the RVD of repeat 1 (RVD1; HD1 to NS1) (Figure 3B). Interestingly, NS1 led to a very good activity of AvrBs3 irrespective of N_0_ in EBE_AvrBs3_ and the presence of tryptophan, cysteine or alanine at position 232 and glycine at position 236 (Figure 3B; Supplementary Figure S5). Expression of all AvrBs3-TalC chimera *in planta* was shown by western blot (Supplementary Figure S6). In conclusion, NS1 broadens the target specificity of AvrBs3 for all four bases at N_0_ and tolerates mutations in the NTR.

### Activity of AvrBs3 with an HD-repeat 1 depends on T_0_

**Figure 4.**
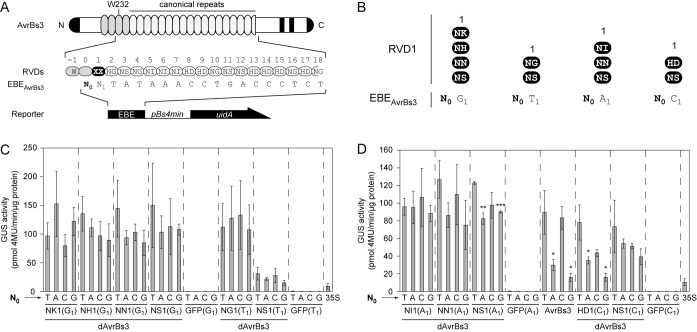
The RVD in repeat 1 of AvrBs3 affects specificity for T_0_ in the DNA target sequence. (**A**) Schematic presentation of AvrBs3-derivatives and DNA EBEs. AvrBs3-derivatives contain different RVDs in repeat 1 (black oval; RVD = XX) and were analyzed with corresponding EBEs (N_1_) that vary in position zero (N_0_). (**B**) Schematic presentation of the analyzed AvrBs3–EBE combinations. (**C** and **D**) GUS activities induced by AvrBs3 and derivatives 3 days after *Agrobacterium*-mediated delivery of effector- and reporter-constructs into leaves of *Nicotiana benthamiana*. Please note that inoculations and tissue harvest for (C) and (D) were performed together for all samples of this experiment, but for technical reasons GUS activities were determined on different days. Asterisks indicate a significant difference in activity of the same TALE-derivative tested with EBE-T_0_ (Student's t-test; *P-value ≤ 0.05; **P-value ≤ 0.01; ***P-value ≤ 0.001). Experiments were performed twice with similar results.

To test whether other RVDs in repeat 1 of AvrBs3 behave similarly to NS1 we replaced HD1 with commonly used RVDs (NK, NH, NN, NG, NI). AvrBs3 activity was assessed using reporters with EBEs based on the optimal recognition specificity of the chosen RVDs (Figure 4A and B. Surprisingly, all analyzed RVDs resulted in good AvrBs3 activity irrespective of N_0_ (Figure 4C and D) suggesting that T_0_ is particularly important if the repeat region starts with HD1. We termed this the ‘rep1 effect’. In addition, AvrBs3 constructs containing NH1, NN1 and NS1 showed a slight preference for T_0_, although differences were not significant. The native AvrBs3 showed an activity comparable to dAvrBs3-HD1 (Figure 4C and D. Relative GUS values of all dAvrBs3-derivatives are summarized in Supplementary Figure S7A, and their expression was confirmed by immunoblot (Supplementary Figure S7B). Notably, dAvrBs3(NS1) displayed robust and T_0_-independent activity with EBEs A_1_/C_1_/G_1_, whereas NS1 together with EBE T_1_ resulted in very low activity with all four reporter constructs (EBE N_0_T_1_) (Figure 4C and D. This is probably due to the conformation of the serine in contact to thymine which is unfavored if present at position 1 (8). Interestingly, dAvrBs3(HD1) displayed similar activities with the EBEs C_1_ and A_1_ keeping the specificity for the base at position 0 (T_0_ > C_0_ > A_0_ > G_0_) (Supplementary Figure S7C). This suggests that adenine at position 1 allows a similar interaction as cytosine with the RVD HD1.

### T_0_-dependency is affected by repeat number and RVD composition

**Figure 5.**
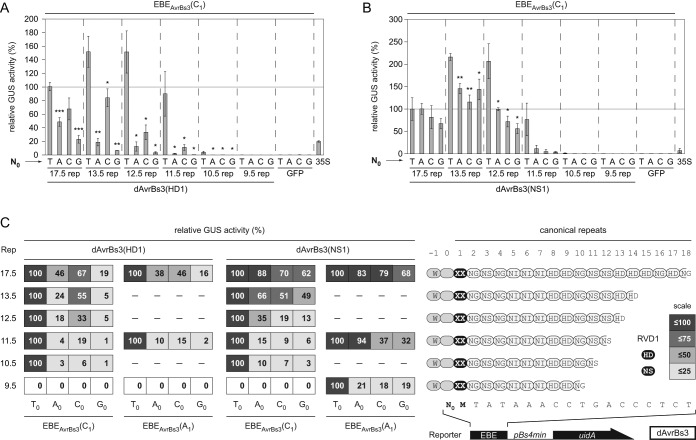
The number of AvrBs3 repeats affects T_0_-dependency. (**A** and **B**) GUS activities induced by AvrBs3 and derivatives 3 days after *Agrobacterium*-mediated delivery of effector- and reporter-constructs into leaves of *Nicotiana benthamiana*. Asterisks indicate a significant difference in activity of the same TALE-derivative tested with EBE-T_0_ (Student's t-test; *P-value ≤ 0.05; **P-value ≤ 0.01; ***P-value ≤ 0.001). (**C**) Comparison of relative GUS activities induced by AvrBs3-derivatives dependent on the base at position N_0_. Constructs and corresponding EBEs are schematically presented in the right panel. AvrBs3 derivatives differ in number of repeats and the repeat 1 RVD (black oval; RVD = XX). Chosen RVD1: HD or NS. The activity of AvrBs3-derivatives for EBE(T_0_) was set to 100%. Relative GUS values are based on the mean of three independent experiments. Color scale: GUS activities smaller than 100%.

Surprisingly, several RVDs in repeat 1 resulted in good AvrBs3 activity irrespective of N_0_ (Figure 4C and D. This result is in contrast to the strong conservation of T_0_ in natural target sequences and published data (6,14,22,23). We reasoned that the T_0_-dependency might be influenced by both repeat number and RVD composition. To address this, we shortened the AvrBs3 repeat region (17.5 repeats) to obtain dAvrBs3 constructs with 13.5 to 9.5 repeats and exchanged HD1 to NS1 because of its broad recognition specificity with the EBEs N_0_C_1_ and N_0_A_1_ (Figure 5). Although there is a tendency for T_0_ preference, dAvrBs3(NS1)-17.5 displayed no significant difference in activity with different bases at N_0_, whereas the activity of dAvrBs3(HD1)-17.5 depended on N_0_ with the hierarchy T_0_ > C_0_ > A_0_ > G_0_ (‘rep1 effect’; Figure 5A and B.

Interestingly, reducing the number of repeats increased T_0_-dependency in all cases even if the repeat region starts with NS1. While AvrBs3 constructs carrying 13.5 and 12.5 repeats displayed higher activity than AvrBs3 with 17.5 repeats, activities of AvrBs3 constructs with 11.5 repeats were comparable and T_0_-dependent. AvrBs3 constructs (HD1 and NS1) with 10.5 and 9.5 repeats showed weak and no activity, respectively, if combined with the EBEs N_0_C_1_ (Figure 5A and B. All AvrBs3 derivatives were stably expressed (Supplementary Figure S8A). Notably, dAvrBs3(NS1)-11.5 displayed increased activity and reduced T_0_-dependency if combined with EBE N_0_A_1_ instead of EBE N_0_C_1_ (Figure 5C; Supplementary Figure S8B). Furthermore, if we compare the results for dAvrBs3(HD1)-11.5 rep with EBE_AvrBs3_(C_1_) and dAvrBs3(NS1)-11.5 rep with EBE_AvrBs3_(A_1_) the influence of RVD1 on T_0_-dependency becomes obvious (‘rep1 effect’) (Figure 5C). Altogether, these data corroborate the need for T_0_ in case of a short repeat region.

**Figure 6.**
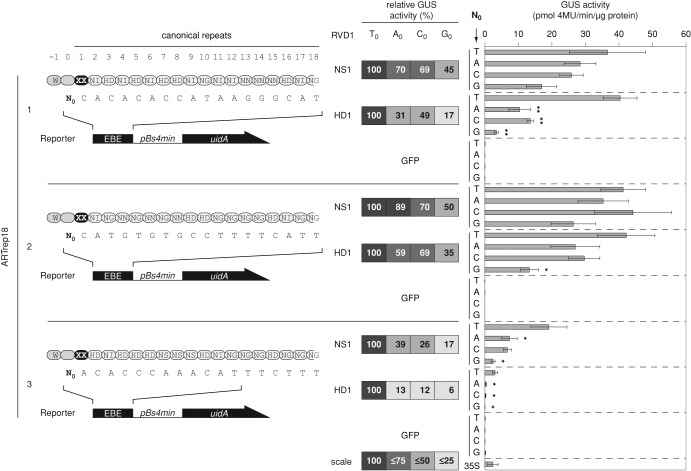
The effect of the TALE RVD-composition on T_0_-dependency. GUS activities induced by ARTrep18-derivatives 2 days after *Agrobacterium*-mediated delivery of effector- and reporter-constructs into leaves of *Nicotiana benthamiana*. Asterisksindicate a significant difference in activity of the same TALE-derivative tested with EBE-T_0_ (Student's t-test; *P-value ≤ 0.05; **P-value ≤ 0.01; ***P-value ≤ 0.001). Constructs and corresponding EBEs are schematically presented in the left panel. TALEs (ARTrep18) differ in the repeat 1 RVD (black oval; RVD = XX). RVD1: NS and HD. Relative GUS activities are given in the middle. TALE activity with EBE(T_0_) was set to 100%. Relative GUS values are based on the mean of three independent experiments. Color scale: GUS activities smaller than 100%.

Next, we investigated whether the T_0_-dependency and ‘rep1 effect’ are influenced by the RVD composition. For this, we constructed six artificial TALEs consisting of 17.5 repeats that differ in the RVD-composition (ARTrep18-1, ARTrep18-2 and ARTrep18-3) and RVD1 (HD1 or NS1; Figure 6). Activities of ARTrep18-1 and ARTrep18-2 with the corresponding EBEs (T_0_) were comparable, whereas ARTrep18-3 displayed reduced activity (Figure 6A). It appears that a highly active TALE is less dependent on T_0_ (e.g. ARTrep18-2). TALEs with HD1 and EBE G_0_ showed the lowest activity in each case (Figure 6B). Expression of all constructs was shown by western blot (Figure S9C).

Taken together, the results show a clear hierarchical T_0_-dependency of TALEs: repeat number, the RVD-composition and the ‘rep 1’ effect (compare Figures 5 and 6).

## DISCUSSION

### T_0_-dependency and ‘rep1 effect’

TALE-derived DNA-binding domains serve as powerful tools to direct executor domains to desired target sequences. A considerable constraint is the dependency on a thymine at position zero of the EBE(T_0_). To be able to change the specificity of T_0_ one needs to understand the molecular mechanism of T_0_ coordination. Here, this question was addressed by studying derivatives of AvrBs3 and artificial TALEs. Our data show that the T_0_-dependency of TALEs is affected by the overall RVD-composition and increases with less repeats and if the EBE sequence contains mismatches. Finally, we discovered that repeat 1 cooperates with the degenerated repeats and that the RVD in repeat 1 affects the nucleotide specificity for T_0_. T_0_ appears to be particularly important if the RVD of the first repeat is HD (‘rep1 effect’; please see the statistical evaluation in Supplementary Figure S9). We think that the T_0_-dependency decreases in case of high DNA-binding affinity provided by a well-balanced RVD-composition of the canonical repeats. It was recently shown that the DNA-binding affinity of a given TALE depends on the overall RVD-composition (24).

Natural TALE EBEs almost always start with T_0_ and, to our knowledge, never correspond to the TAL code-deduced optimal DNA sequence. Furthermore, the amount of TALE proteins secreted into the plant cell by *Xanthomonas* is much lower than the amount of TALE molecules produced by *35S*-driven expression in the eukaryotic cell. We, therefore, believe that in nature TALE activity requires T_0_ in the corresponding EBEs. Nevertheless, a natural TALE consisting of at least 17.5 repeats with a well-balanced RVD-composition might induce target genes independent of T_0_ as exemplified by TalC (15). Hence, we suggest to consider the T_0_-dependency and ‘rep1 effect’ in off-target predictions for artificial TALEs and TALENs which are usually designed to match perfectly to the target sequence. Taken together, our results suggest the following hierarchy for T_0_-independend TALEs: first, repeat number (17.5), followed by the RVD-composition (well-balanced) and finally the ‘rep1 effect’ (no HD1) (compare Figures 5 and 6).

### Generation of T_0_-independent NTRs

Structural data suggested that the tryptophan in repeat −1 (W232) of PthXo1 coordinates T_0_ (10). We, therefore, analyzed whether the T_0_ specificity of AvrBs3 can be changed by W232 substitutions. Although AvrBs3-derivatives with W232 substitutions to aromatic side chains retained considerable activity they were less active. Obviously, T_0_ specificity could not easily be changed by single amino acid substitutions suggesting that T_0_ coordination is more complex and involves additional residues. The need for an aromatic side chain at position 232 in AvrBs3 and T_0_ appears to depend on the RVD HD1. This hypothesis is supported by (i) the natural TALE TalC, which contains a tryptophan to cysteine substitution in repeat −1 and a first canonical repeat with the RVD NS1 and by (ii) the functionality of W232C and W232A mutations in AvrBs3 if the repeat region starts with NS1. Our data provide additional explanations to previous studies, in which W232 of TALEs was mutated (17,25). Notably, we confirmed data obtained by Tsuji *et al.* (25) for a dTALE that starts with HD1 and contains 14.5 repeats. In contrast, Doyle *et al.* (17) obtained variable results for W232 substitutions in PthXo1 (24.3 repeats; NN1; EBE with four mismatches) and dTALE868 (14.5 repeats; NI1; optimal EBE). In the latter case, however, the results were reported to be highly variable (17). Recently, T_0_-independent NTRs were generated by mutation of the ‘RVD-loop’ of repeat −1 (22,25). Notably, a G_0_-specific NTR was generated by the double amino acid substitution W232R/Q231S in the dTALE Avr15 (14.5 repeats; NI1; optimal EBE) (22). In our study, TALEs, containing HD1 always worked less well if combined with an EBE that starts with G_0_. Interestingly, A_0_-specific and C_0_-specific but not G_0_-specific NTRs combined with a repeat region that starts with HD1 were described (25). This might be due to the fact that G_0_ in EBEs of TALEs is unfavored if the first RVD of the canonical repeats is HD1 (as seen with AvrBs3 and the ARTrep18 constructs). One of the novel findings of our study is that a desired change in T_0_ specificity by mutagenesis of the TALE NTR needs to consider different RVDs in repeat 1.

### W232 and T_0_ facilitate interaction of the canonical repeats with the target sequence

**Figure 7.**
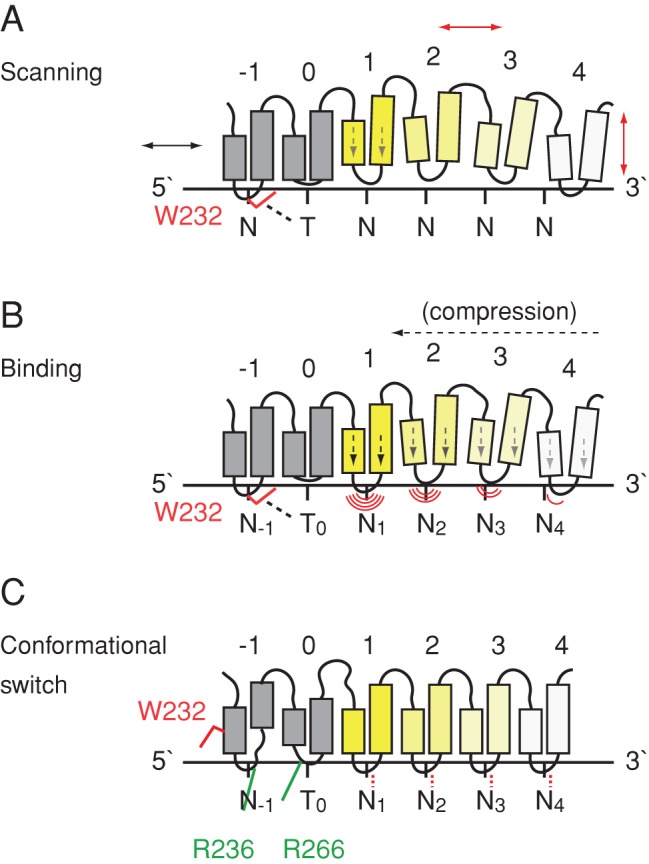
Model of T_0_-coordination by TALE proteins. TALE repeats are coordinated along the sugar-phosphate backbone of the DNA sense strand. Degenerated repeats are indicated in gray, canonical repeats are indicated in yellow and light yellow. (**A**) Upon DNA target scanning the canonical repeats shift laterally and vertically (red arrows) due to sterical clashes between residue 13 and non-matching bases. Thymine, which interacts with tryptophan (W232) stabilizes the interaction between RVD1 and base 1. (**B**) Once the target sequence is bound W232 and T_0_ facilitates interaction of RVD1 and N_1_. Specific interactions of RVDs and matching bases lead to compression of the canonical repeats. Necessity of specific RVD-base interactions (indicated in red) decreases from 5′ to 3′. (**C**) Compression of the canonical repeats induce conformational switch of the NTR, leading to exposure of R236 and R266 in the major groove.

Previously, it was suggested that the NTRs of TALEs serve as nucleation site for the DNA interaction (9,24). To integrate our data we propose the following model (Figure 7). TALEs may slide along the DNA scanning for the target sequence. Once the target sequence is reached, specific contacts between the canonical repeats and nucleobases occur from 5′ to 3′ and allow the repeats to compress (8,9,24). Taking this idea into account we hypothesize that the W232-T_0_ interaction facilitates the specific interaction between canonical repeats and target nucleobases which may be more crucial if an HD1 contact to C_1_ needs to be established (Figure 7). We can only speculate about the strong dependency of TALEs with HD1 on T_0_ and W232. One explanation could be that the amine group of cytosine targeted by HD is more distant from the sugar phosphate backbone than N7 of guanine and adenine or the methyl group of thymine targeted by other RVDs (Supplementary Figure S10). In addition, HD is the only RVD which accepts the hydrogen bond from the base, whereas others donate hydrogen bonds to the base or interact with the base via van der Waals forces (Supplementary Figure S10) (8,10,26).

Based on TALE structures the nucleobase at position zero of a corresponding EBE is contacted by the repeats −1, 0 and 1. Repeat −1 was reported to interact with the base by van der Waals forces (W232-T_0_) whereas repeats 0 and 1 coordinate the phosphate group of T_0_ via direct (*oxyanion clip*) and water-mediated hydrogen bonds (8,10–12). In contrast to Mak *et al.* (10), a recent published structure of DNA-bound AvrBs3 suggests an alternative conformation of repeat −1 and 0, in which T_0_ is coordinated by R266 and not by W232 (11). Notably, the DNA fragment used for crystallization of DNA-bound AvrBs3 started at position −2, i.e. it did not allow the interaction of the complete NTR with the DNA (11). The latter, however, may stabilize the conformation of the NTR in a DNA-bound TALE (10). In both TALE–DNA complexes repeats −2 and −3 are disordered. Mutation of R266 in AvrBs3 slightly reduced the overall AvrBs3 activity but did not alter T_0_ specificity. We, therefore, believe that the suggested role of R266 in T_0_-coordination (11) is unlikely. This is corroborated by the fact that changes in T_0_ specificity were accomplished by mutation of W232 and neighboring residues (22,25). Considering a conformational switch of TALEs upon target binding one cannot exclude that the structure reported by Stella *et al.* (11) may represent a stable state conformation of a DNA-bound TALE. Compression of the canonical repeats upon specific target binding may trigger a conformational switch of the NTR. In this case, W232–T_0_ interaction might be relevant for TALE target finding and the conformational switch initiation (Figure 7). Notably, TALE flexibility was underpinned by molecular dynamics simulation (12,27). To reveal the details of TALE–DNA interactions further structural analyzes of TALEs with T_0_-independent NTRs and TALE–DNA complexes are required.

The data presented here provide explanations for reported variations in T_0_-specificity of different TALEs and give novel insights into the mechanism of T_0_-recognition by TALE proteins. Our findings will improve the design of customized TALE-based DNA-binding proteins, generation of T_0_-independent NTRs, target prediction and off-target prevention.

## SUPPLEMENTARY DATA

Supplementary Data are available at NAR Online, including [1–3].

SUPPLEMENTARY DATA
