# Supplementary material for: Repeat 1 of TAL effectors affects target specificity for the base at position zero
Source: Nucleic Acids Res. 2014 May 3;42(11):7160–9. doi: 10.1093/nar/gku341 (PMC4066769; doi:10.1093/nar/gku341)
Supplement: SUPPLEMENTARY DATA [file supp_42_11_7160__index.html]

Repeat 1 of TAL effectors affects target specificity for the base at position zero — Repeat 1 of TAL effectors affects target specificity for the base at position zero — SUPPLEMENTARY DATA 

# Repeat 1 of TAL effectors affects target specificity for the base at position zero

## SUPPLEMENTARY DATA

**Files in this Data Supplement:**

- SUPPLEMENTARY DATA
- SUPPLEMENTARY DATA
