# Supplementary material for: Repeat 1 of TAL effectors affects target specificity for the base at position zero
Source: Nucleic Acids Res. 2014 May 3;42(11):7160–9. doi: 10.1093/nar/gku341 (PMC4066769; doi:10.1093/nar/gku341)
Supplement: SUPPLEMENTARY DATA [file supp_gku341_nar-03692-h-2013-File009.pdf]

Figure S1

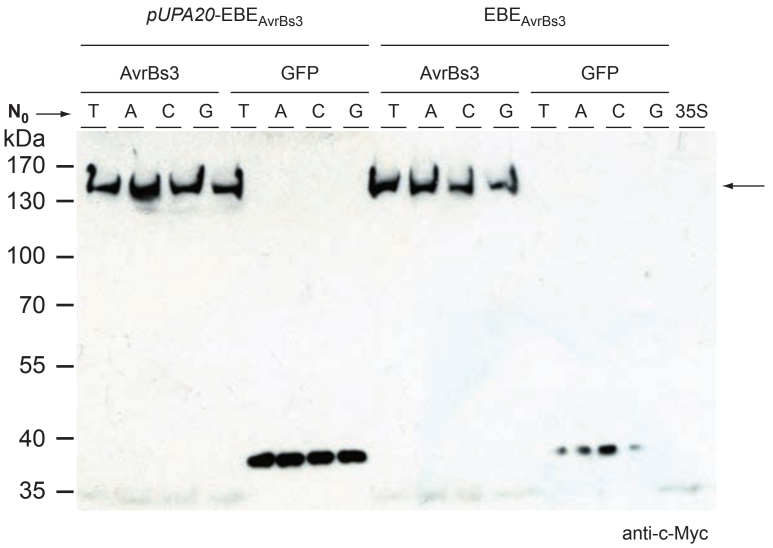

Figure S1. *In planta* expression of effector proteins. Expression of AvrBs3 and GFP fused to an N-terminal c-Myc epitope was confirmed by western blot using an anti-c-Myc antibody. Samples correspond to data presented in Figure 1. The arrow indicates the expected protein size.

Figure S2

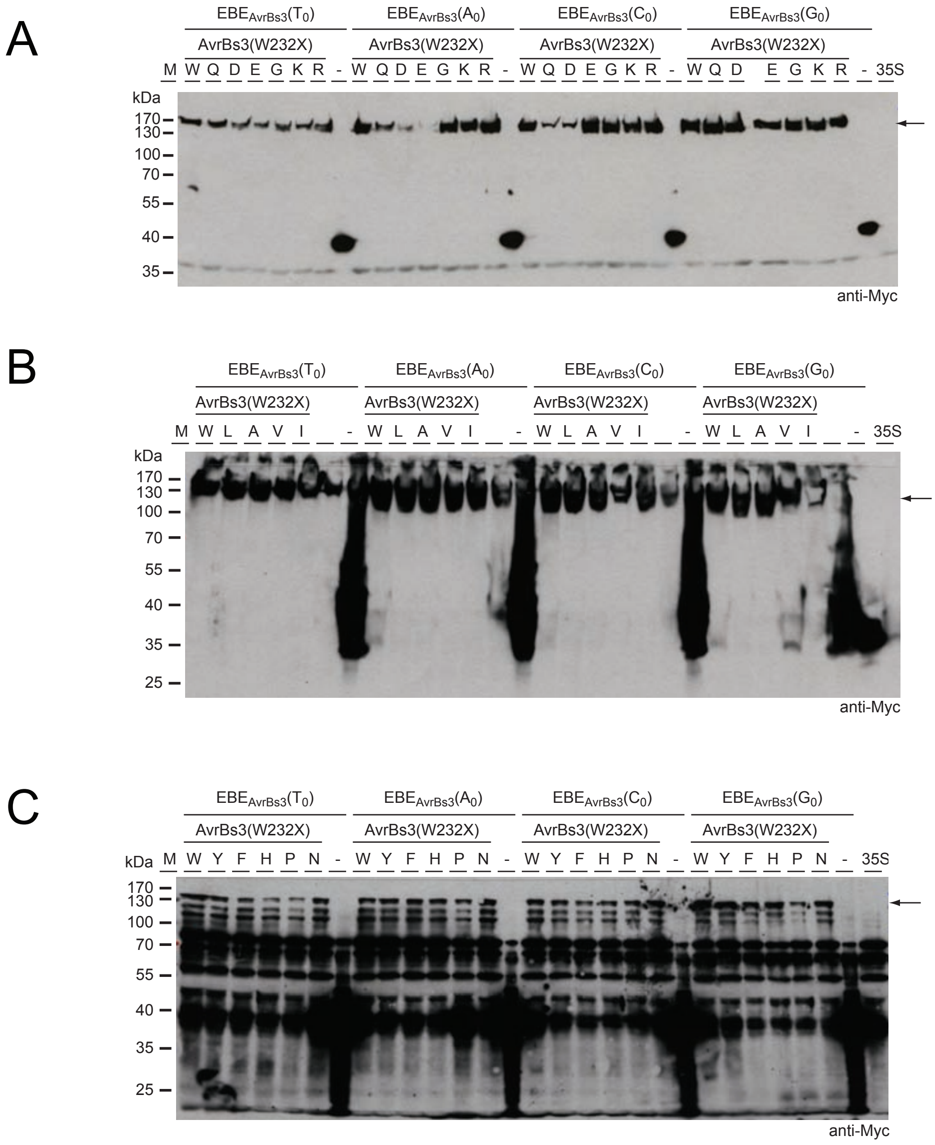

Figure S2. *In planta* expression of effector proteins. **(A, B and C)** Stable expression of AvrBs3, AvrBs3 W232 mutant-derivatives and GFP fused to an N-terminal c-Myc epitope was confirmed by western blot using an anti-c-Myc antibody. Samples correspond to data presented in Figure 2B. Note that protein amounts varied between independent experiments and were not correlated with GUS activities. The arrow indicates the expected protein size. Lanes marked with “-“ represent loading of GFP samples.

Figure S3

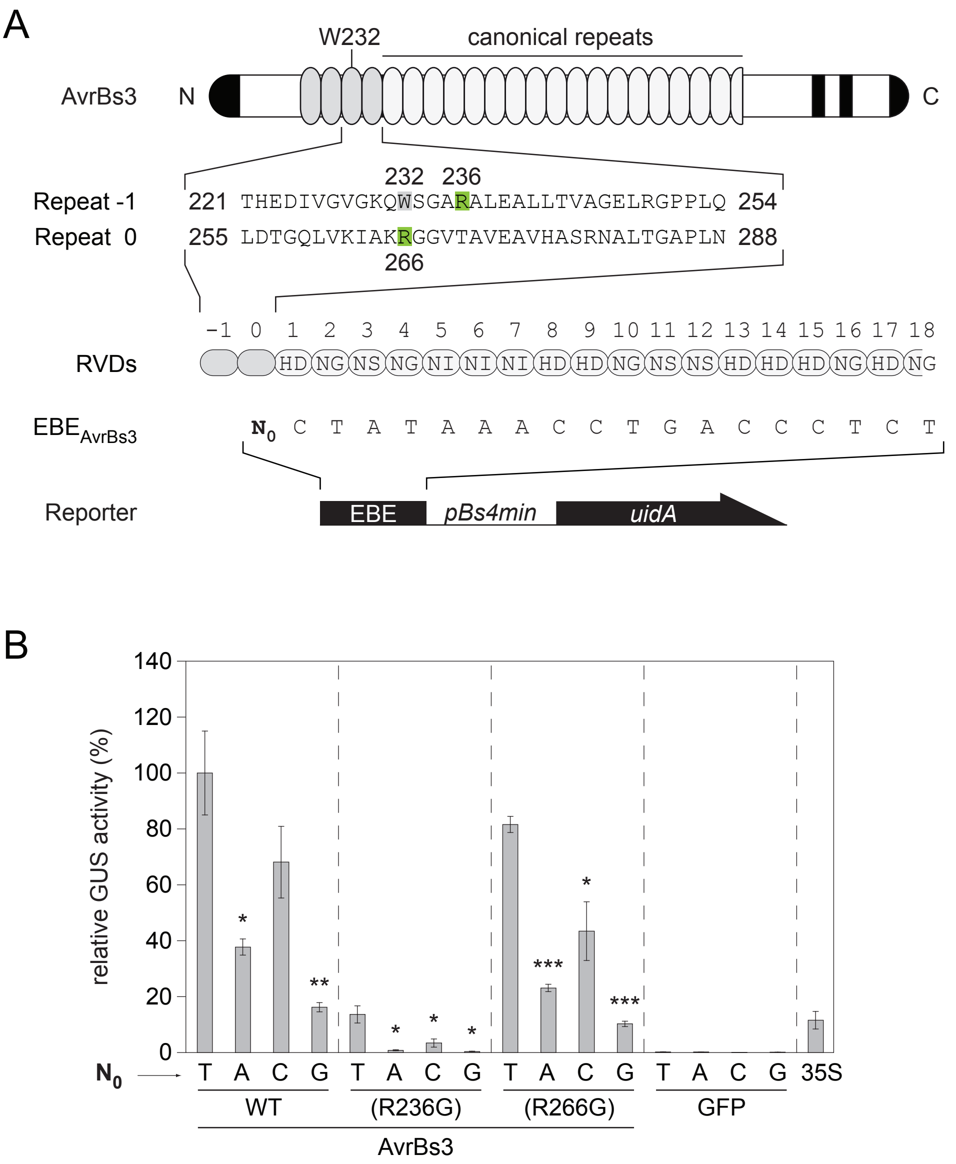

Figure S3. Effect of non-W232 substitutions on AvrBs3 activity. **(A)** Schematic presentation of AvrBs3 and reporter constructs. The amino acid sequence of repeats -1 and 0 is given. Mutated residues R236 and R266 are highlighted in green. **(B)** Relative GUS activities (%) induced by AvrBs3 and derivatives 3 days after *Agrobacterium*-mediated delivery into leaves of *N. benthamiana*. AvrBs3(WT) activity with EBE(T_0) was set to 100%. Experiments were performed three times with similar results.

Figure S4

A

```
                                                                    repeat -1
TalC        1 MDPIRPRAPSPAREVLPGPQPDRVQPTADRGVSAPAGSPLDGLPARRTMSRTRLPSPPAP
AvrBs3      1 MDPIRSRTPSPARELLPGPQPDGVQPTADRGVSPPAGGPLDGLPARRTMSRTRLPSPPAP
consensus   1 MDPIR R PSPAREvLPGPQPD VQPTADRGVS PAG PLDGLPARRTMSRTRLPSPPAP

TalC       61 LPAFSAGSFSDLLRQFDPSLLDTSLFDSMPAVGTPHTEAAPAEGDEVQSALRAADDPPPT
AvrBs3     61 SPAFSAGSFSDLLRQFDPSLFNTSLFDSLPPFGAHHTEAATGEWDEVQSGLRAADAPPPT
consensus  61  PAFSAGSFSDLLRQFDPSL  TSLFDSmP   G   HTEAA aE DEVQSaLRAAD PPPT

TalC      121 VRVAVTAA-----------------------QVDLRTLGYSQQQ-EKIKPNVRSTVAQHH
AvrBs3    121 MRVAVTAARPPRAKPAPRRRAAQPSDASPAAQVDLRTLGYSQQQQEKIKPKVRSTVAQHH
consensus 121 vRVAVTAArpprakpaprrraaqpsdaspaaQVDLRTLGYSQQQqEKIKP VRSTVAQHH

                                                                   repeat -1
TalC      157 EALVGHGFTHAHIVALSRHPAALGTVAVKYQDMIAALPEATHEDIVGVGKQCSGARALEA
AvrBs3    181 EALVGHGFTHAHIVALSQHPAALGTVAVKYQDMIAALPEATHEAIVGVGKQWSGARALEA
consensus 181 EALVGHGFTHAHIVALS HPAALGTVAVKYQDMIAALPEATHE IVGVGKQ SGARALEA

                                         repeat 0
TalC      217 LLTVAGELRGPPLQLDTGQLVKIAKRGGVTAVEAVHASRNALTGAPLNLTPAQVVAIASN
AvrBs3    241 LLTVAGELRGPPLQLDTGQLLKIAKRGGVTAVEAVHAWRNALTGAPLNLTPEQVVAIASH
consensus 241 LLTVAGELRGPPLQLDTGQLvKIAKRGGVTAVEAVHA RNALTGAPLNLTP QVVAIAS

              repeat 1
TalC      277 SGGKQALETVQRLLPVLCQAHG 298
AvrBs3    301 DGGKQALETVQRLLPVLCQAHG 322
consensus 301  GGKQALETVQRLLPVLCQAHG
```

B

Repeat	01	02	03	04	05	06	07	08	09	10	11	12	13	14	15	16	17	18	19	20	21	22
**TalC**	NS	NG	NS	HD	NI	NG	NN	NG	HD	NI	NN	N*	NI	NN	HD	NG	NI	NN	N*	HD	NN	NG
**AvrBs3**	HD	NG	NS	NG	NI	NI	NI	HD	HD	NG	NS	NS	HD	HD	HD	NG	HD	NG				

Figure S4. Amino acid sequence comparison of AvrBs3 and TalC. **(A)** Amino acid comparison of the NTR and repeat 1 of AvrBs3 (1) and TalC (2). The alignment was done using the boxshade server (http://www.ch.embnet.org/software/BOX_form.html). Red lines mark the ends of repeat -1, 0 and 1. **(B)** Comparison of the RVD order in AvrBs3 and TalC.

Figure S5

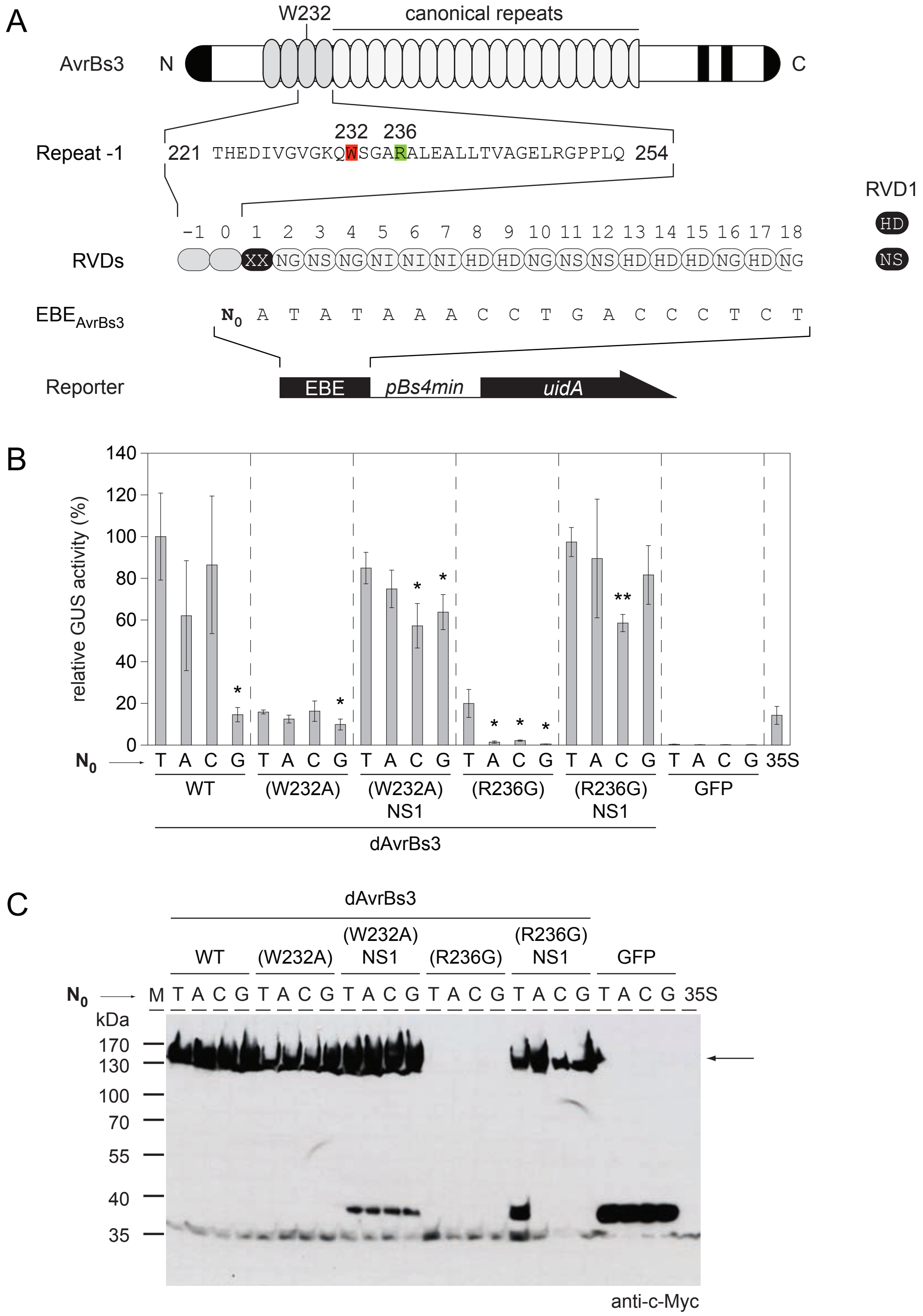

Figure S5. Activity of AvrBs3 with the RVD1 NS is independent of W232 and R236. **(A)** Schematic presentation of AvrBs3-derivatives and reporter constructs. The amino acid sequence of repeats -1 is given. Mutated residues W232 and R236 are highlighted in red and green, respectively. **(B)** Relative GUS activities (%) induced by AvrBs3 and derivatives 3 days after *Agrobacterium*-mediated delivery into leaves of *N. benthamiana*. AvrBs3(WT) activity with EBE(T_0) was set to 100%. Experiments were performed twice with similar results. **(C)** Expression of AvrBs3, AvrBs3-derivatives and GFP fused to an N-terminal c-Myc epitope was confirmed by western blot using an anti-c-Myc antibody.

Figure S6

A

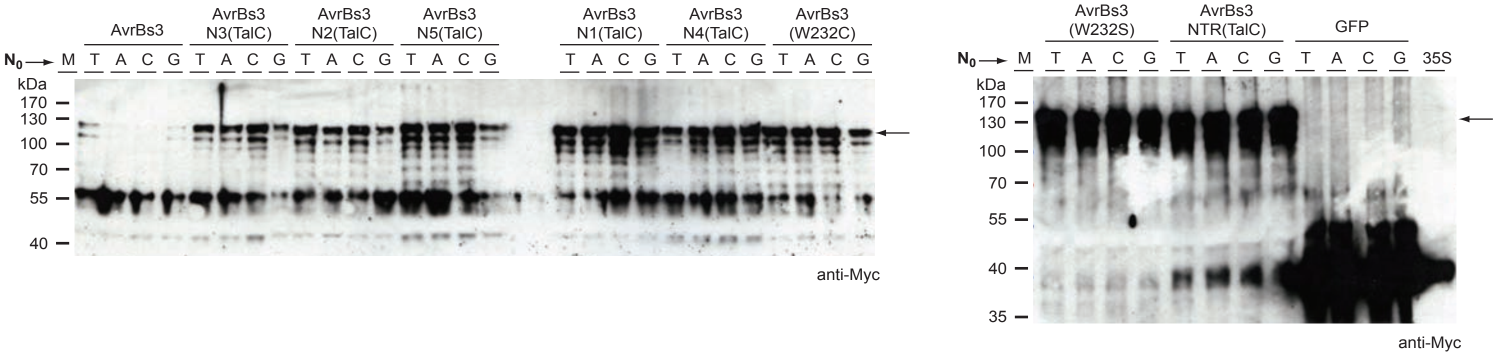

B

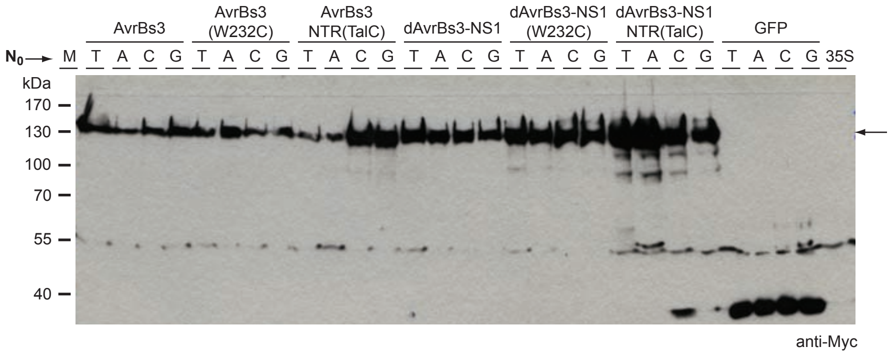

Figure S6. *In planta* expression of effector proteins. **(A, B and C)** Stable expression of AvrBs3, AvrBs3-derivatives and GFP fused to an N-terminal c-Myc epitope was confirmed by western blot using an anti-c-Myc antibody. Samples correspond to data presented in Figure 3. The arrow indicates the expected protein size. Lanes marked with “-“ represent loading of GFP samples. Note that protein amounts vary between independent experiments and are not correlated with GUS activities.

Figure S7

A

AvrBs3	GUS activity (T_0) rel. to AvrBs3 (WT)	relative GUS activity (N_0)			
		T_0	A_0	C_0	G_0
NN1(A_1)	186.3 ± 63.4	100	65.8 ± 3.1	82.8 ± 5.3	56.9 ± 3.3
NS1(A_1)	164.7 ± 38.9	100	76.5 ± 13.2	79.3 ± 0.3	69.9 ± 4.7
NI1(A_1)	164.3 ± 81.0	100	89.5 ± 13.8	91.9 ± 27.2	84.2 ± 11.2
NS1(G_1)	144.8 ± 8.0	100	68.8 ± 0.1	61.6 ± 19.4	60.5 ± 16.1
NN1(G_1)	125.2 ± 12.2	100	71.7 ± 10.0	69.0 ± 3.2	58.7 ± 0.1
NH1(G_1)	120.8 ± 6.7	100	80.2 ± 2.3	73.9 ± 3.5	67.8 ± 2.7
**WT(C_1)**	**100**	**100**	**25.6 ± 11.0**	**85.3 ± 10.9**	**14.1 ± 5.2**
NG1(T_1)	97.3 ± 9.4	100	86.4 ± 38.6	104.0 ± 20.6	75.6 ± 28.5
HD1(C_1)	95.9 ± 12.4	100	46.0 ± 1.4	62.3 ± 9.0	19.1 ± 2.3
NK1(G_1)	83.5 ± 8.6	100	129.0 ± 40.7	70.9 ± 16.1	108.8 ± 24.6
NS1(C_1)	79.0 ± 4.2	100	70.7 ± 4.4	65.1 ± 6.9	52.5 ± 1.3
NS1(T_1)	27.9 ± 1.1	100	72.1 ± 2.6	72.1 ± 26.0	48.6 ± 0.5

scale	>100	≤100	≤75	≤50	≤25

B

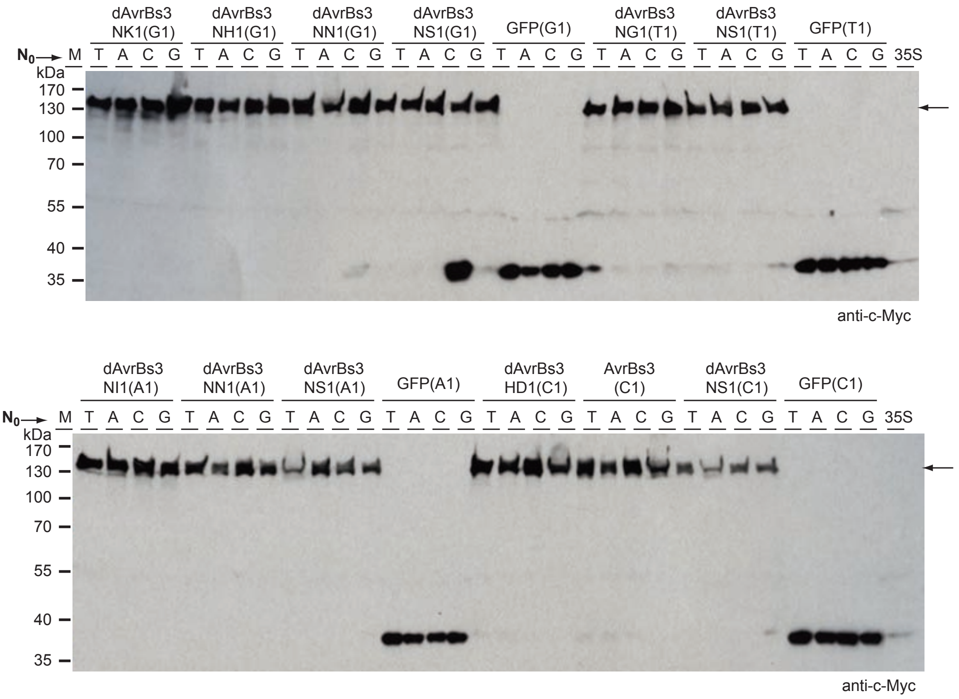

C

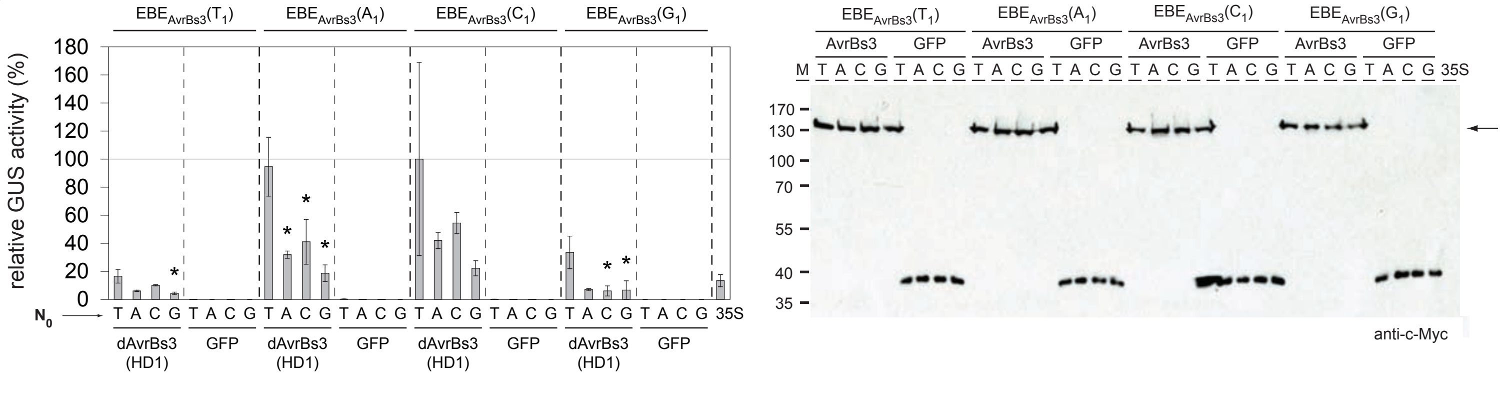

Figure S7. (A) Relative activity of AvrBs3-derivatives differing in the RVD1. Relative GUS activities (%) induced by AvrBs3 and derivatives 3 days after *Agrobacterium*-mediated delivery of the constructs into leaves of *N. benthamiana*. The AvrBs3-derivatives were ranked according to their activity; the activity of AvrBs3 with EBE(T_0) was set to 100%. Mean values and the standard deviation of means from two independent experiments with similar results are shown. Color scale: GUS activities higher and smaller than 100%. **(B)** *In planta* expression of effector proteins. AvrBs3, AvrBs3-derivatives and GFP were expressed as N-terminal c-Myc epitope fusions and analyzed by western blot using an anti-c-Myc antibody. Arrows indicate the expected size of the fusion proteins. Samples correspond to data points presented in Figure 4. Note that protein amounts varied between independent experiments and were not correlated with GUS activities. **(C)** Relative GUS activities (%) induced by dAvrBs3(HD1) with EBE_{AvrBs3}(N_0 and N_1). Samples were harvested 3 days after *Agrobacterium*-mediated delivery of the constructs into leaves of *N. benthamiana*. AvrBs3(WT) activity with EBE(T_0) was set to 100%. N_0: base at position 0 of the EBE.

Figure S8

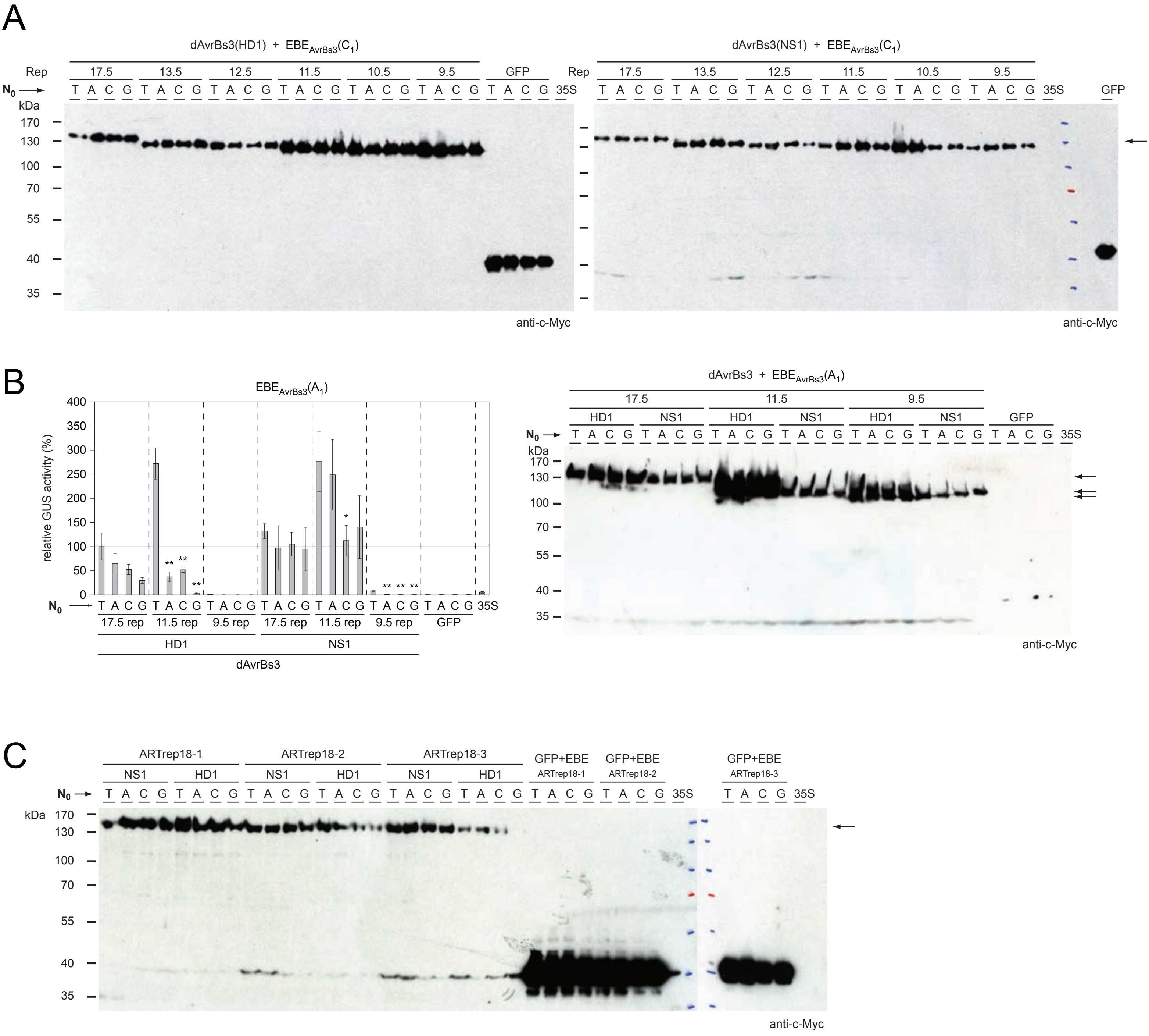

Figure S8. *In planta* expression of TALE proteins. Stable expression of AvrBs3, AvrBs3-derivatives, ARTrep18-derivatives and GFP fused to an N-terminal c-Myc epitope was confirmed by western blot using an anti-c-Myc antibody. Arrows indicate the expected protein sizes. **(A)** Expression analysis of samples corresponding to data points presented in Figure 5A and B. **(B)** GUS activities induced by AvrBs3 and derivatives 3 days after *Agrobacterium*-mediated delivery into leaves of *N. benthamiana*. **(C)** Expression analysis of samples corresponding to data presented in Figure 6A. Experiments were performed three times with similar results. N_0: base at position 0 of the EBE.

Figure S9

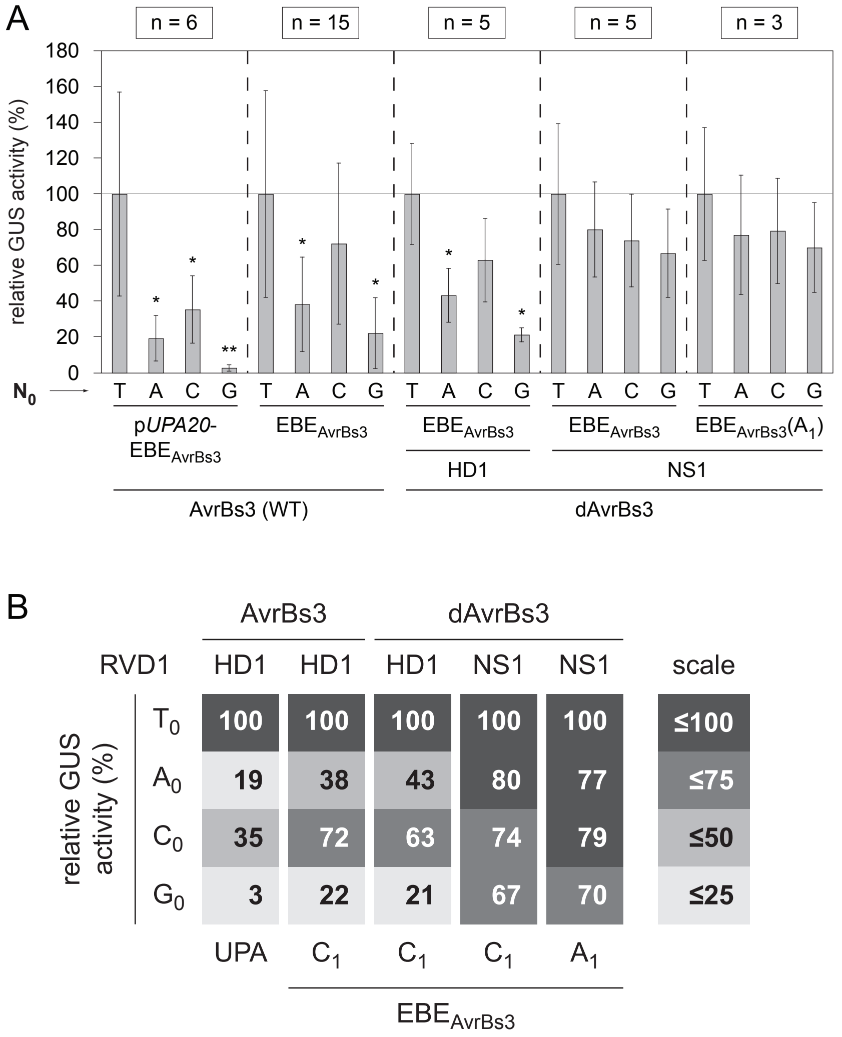

Figure S9. Summary of the “rep1-effect” based on statistical evaluation. **(A)** Standard deviation of means of AvrBs3(HD1), dAvrBs3(HD1) and dAvrBs3(NS1) activities tested on different EBEs. Mean values of single experiments were normalized to 35S:GUS activity. Normalized mean values were used for determination of the average and standard deviation of means. AvrBs3-derivative activity with EBE(T_0) was set to 100%. Numbers of independent biological replicates (n) are given above the diagram. Asterisks indicate a significant difference in activity of the same AvrBs3-derivative tested with EBE-T_0 (student’s t-test; * p-value≤ 0.05; ** p-value≤0.01; *** p-value≤0.001). **(B)** Summary of the average activities of AvrBs3 and derivatives. Color scale: GUS activities smaller than 100%.

Figure S10

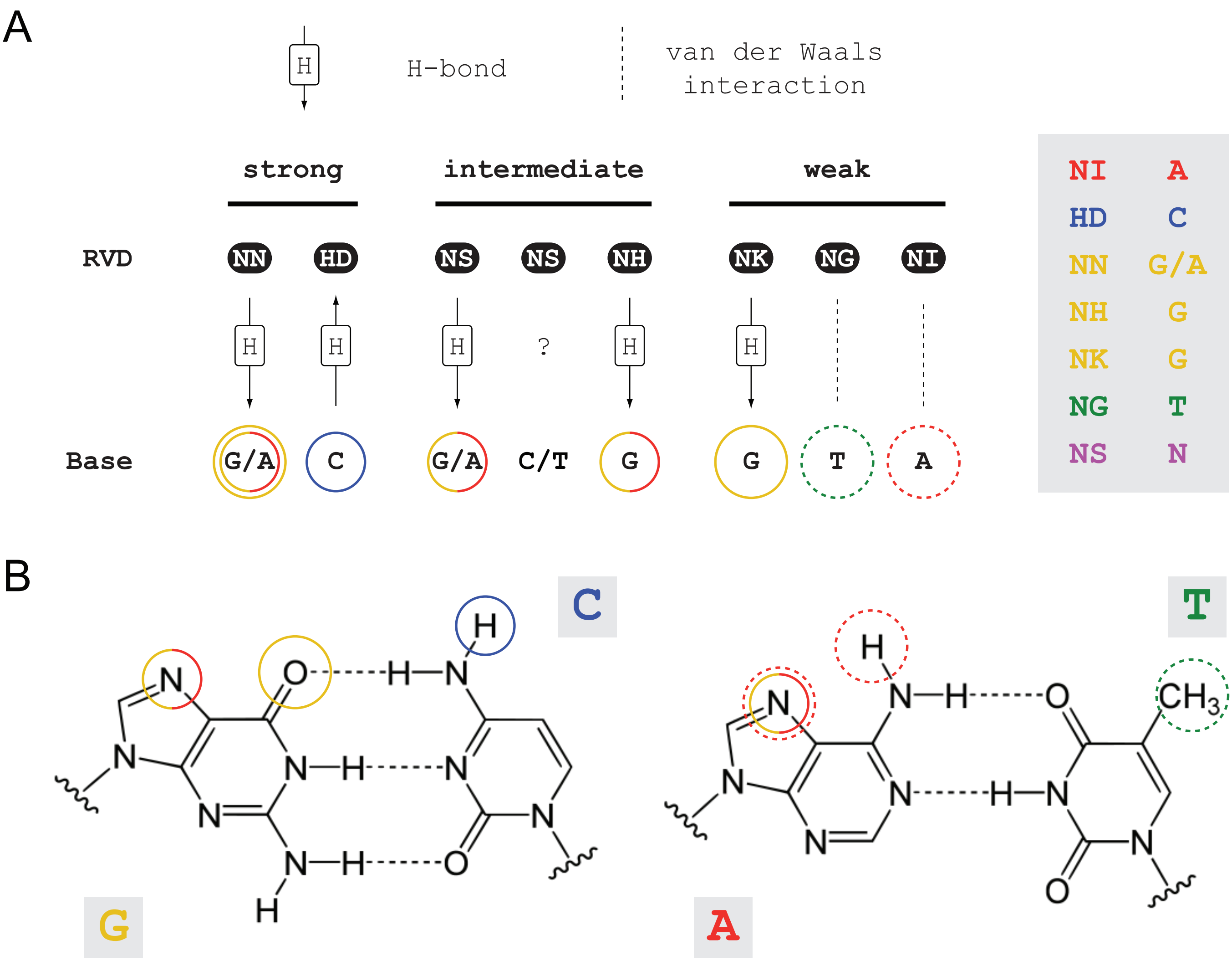

Figure S10. Summary of TALE RVD-base interactions of RVDs used in this study. **(A)** RVD-base interactions are indicated by arrows and dashed lines for hydrogen (H-) bonds and van der Waals interactions, respectively. Arrowheads indicate the direction of H-bond donor-acceptor. Classification of RVDs in strong, intermediate and weak RVDs as described (3). The summary of RVDs analyzed and specificities are given in the box on the right. **(B)** Specific contacts between RVDs and corresponding bases are indicated by circles. The color-code is based on RVD-base specificity. Hydrogen-bonds and van der Waals interactions are indicated in circles and dashed circles, respectively.

Supporting references

1. Bonas, U., Stall, R.E. and Staskawicz, B. (1989) Genetic and structural characterization of the avirulence gene *avrBs3* from *Xanthomonas campestris* pv. *vesicatoria*. *Mol. Gen. Genet.*, **218**, 127-136.

2. Yu, Y., Streubel, J., Balzergue, S., Champion, A., Boch, J., Koebnik, R., Feng, J., Verdier, V. and Szurek, B. (2011) Colonization of rice leaf blades by an African strain of *Xanthomonas oryzae* pv. *oryzae* depends on a new TAL effector that induces the rice nodulin-3 *Os11N3* gene. *Mol Plant Microbe Interact*, **24**, 1102-1113.

3. Streubel, J., Blücher, C., Landgraf, A. and Boch, J. (2012) TAL effector RVD specificities and efficiencies. *Nat. Biotechnol.*, **30**, 593-595.
