# Supplementary material for: Repeat 1 of TAL effectors affects target specificity for the base at position zero
Source: Nucleic Acids Res. 2014 May 3;42(11):7160–9. doi: 10.1093/nar/gku341 (PMC4066769; doi:10.1093/nar/gku341)
Supplement: SUPPLEMENTARY DATA [file supp_gku341_nar-03692-h-2013-File010.doc]

Supplementary Information

**Repat 1 of TAL effectors affects target specificity for base at position zero**

**Tom Schreiber and Ulla Bonas**

Department of Genetics, Martin Luther University Halle-Wittenberg, Weinbergweg 10, D-06120, Halle (Saale), Germany

Corresponding author:

Ulla Bonas, [ulla.bonas@genetik.uni-halle.de](mailto:ulla.bonas@genetik.uni-halle.de)

Tom Schreiber, [tom.schreiber@genetik.uni-halle.de](mailto:tom.schreiber@genetik.uni-halle.de)

**SUPPLEMENTARY SEQUENCES**

**Reporter constructs**

**EBE_AvrBs3_**

**N_0_/N_1_**

**Mismatches**

CDS *uidA*

**pGWB3:*pUPA20*-EBE_AvrBs3_-*pBs4min***

CACC**NA**TATAAACCTGACCCT**T**TTTCTTTCTTGTATATAACTTTGTCCAAAATATCATCAATTGATCTCATCCATACAATTTATTTTTAATCGAATCTAAGGGTGGGCGCGCCGACCCAGCTTTCTTGTACAAAGTGGTTCGATCTAGAGGATCCCCGGGTGGTCAGTCCCTTATG... CDS *uidA*

**pGWB3:EBE_AvrBs3_-*pBs4min***

CACC**NN**TATAAACCTGACCCTCTTTCTTTCTTGTATATAACTTTGTCCAAAATATCATCAATTGATCTCATCCATACAATTTATTTTTAATCGAATCTAAGGGTGGGCGCGCCGACCCAGCTTTCTTGTACAAAGTGGTTCGATCTAGAGGATCCCCGGGTGGTCAGTCCCTTATG... CDS *uidA*

**pGWB3:EBE_ARTrep18-1_-*pBs4min***

CACC**N**CACACACCATAAGGGCATTTCTTTCTTGTATATAACTTTGTCCAAAATATCATCAATTGATCTCATCCATACAATTTATTTTTAATCGAATCTAAGGGTGGGCGCGCCGACCCAGCTTTCTTGTACAAAGTGGTTCGATCTAGAGGATCCCCGGGTGGTCAGTCCCTTATG... CDS *uidA*

**pGWB3:EBE_ARTrep18-2_-*pBs4min***

CACC**N**CATGTGTGCCTTTTCATTTTCTTTCTTGTATATAACTTTGTCCAAAATATCATCAATTGATCTCATCCATACAATTTATTTTTAATCGAATCTAAGGGTGGGCGCGCCGACCCAGCTTTCTTGTACAAAGTGGTTCGATCTAGAGGATCCCCGGGTGGTCAGTCCCTTATG... CDS *uidA*

**pGWB3:EBE_ARTrep18-3_-*pBs4min***

CACC**N**ACACCCAAACATTTCTTTCTTGTATATAACTTTGTCCAAAATATCATCAATTGATCTCATCCATACAATTTATTTTTAATCGAATCTAAGGGTGGGCGCGCCGACCCAGCTTTCTTGTACAAAGTGGTTCGATCTAGAGGATCCCCGGGTGGTCAGTCCCTTATG... CDS *uidA*

**Effector constructs**

**DNA sequence *avrBs3* submodules**

**N-terminal module *avrBs3* (NTR)**

TTGGTCTCATATGGATCCCATTCGTTCGCGCACACCAAGTCCTGCCCGCGAGCTTCTGCCCGGACCCCAACCCGATGGGGTTCAGCCGACTGCAGATCGTGGGGTGTCTCCGCCTGCCGGCGGCCCCCTGGATGGCTTGCCCGCTCGGCGGACGATGTCCCGGACCCGGCTGCCATCTCCCCCTGCCCCCTCACCTGCGTTCTCGGCGGGCAGCTTCAGTGACCTGTTACGTCAGTTCGATCCGTCACTTTTTAATACATCGCTTTTTGATTCATTGCCTCCCTTCGGCGCTCACCATACAGAGGCTGCCACAGGCGAGTGGGATGAGGTGCAATCGGGTCTGCGGGCAGCCGACGCCCCCCCACCCACCATGCGCGTGGCTGTCACTGCCGCGCGGCCGCCGCGCGCCAAGCCGGCGCCGCGACGACGTGCTGCGCAACCCTCCGACGCTTCGCCGGCCGCGCAGGTGGATCTACGCACGCTCGGCTACAGCCAGCAGCAACAGGAGAAGATCAAACCGAAGGTTCGTTCGACAGTGGCGCAGCACCACGAGGCACTGGTCGGCCATGGGTTTACACACGCGCACATCGTTGCGCTCAGCCAACACCCGGCAGCGTTAGGGACCGTCGCTGTCAAGTATCAGGACATGATCGCAGCGTTGCCAGAGGCGACACACGAAGCGATCGTTGGCGTCGGCAAACAGTGGTCCGGCGCACGCGCTCTGGAGGCCTTGCTCACGGTGGCGGGAGAGTTGAGAGGTCCACCGTTGCAGTTGGACACAGGCCAACTTCTCAAGATTGCAAAACGTGGCGGCGTGACCGCAGTGGAGGCAGTGCATGCATGGCGCAATGCACTGACGGGTGCCCCCCTGAACTGAGACCAAA

**Repeat region module *avrBs3* (repeat region)**

CACTGACGGGTGGGTCTCTGAACCTGACCCCGGAGCAGGTGGTGGCCATCGCCAGCCACGATGGCGGCAAGCAGGCGCTGGAGACGGTGCAGCGGCTGTTGCCGGTGCTGTGCCAGGCCCATGGCCTGACCCCGCAGCAGGTGGTGGCCATCGCCAGCAATGGCGGTGGCAAGCAGGCGCTGGAGACGGTGCAGCGGCTGTTGCCGGTGCTGTGCCAGGCCCATGGCCTGACCCCGCAGCAGGTGGTGGCCATCGCCAGCAATAGCGGTGGCAAGCAGGCGCTGGAGACGGTGCAGCGGCTGTTGCCGGTGCTGTGCCAGGCCCATGGCCTGACCCCGGAGCAGGTGGTGGCCATCGCCAGCAATGGCGGTGGCAAGCAGGCGCTGGAGACGGTGCAGCGGCTGTTGCCGGTGCTGTGCCAGGCCCATGGCCTGACCCCGGAGCAGGTGGTGGCCATCGCCAGCAATATTGGTGGCAAGCAGGCGCTGGAGACGGTGCAGGCGCTGTTGCCGGTGCTGTGCCAGGCCCATGGCCTGACCCCGGAGCAGGTGGTGGCCATCGCCAGCAATATTGGTGGCAAGCAGGCGCTGGAGACGGTGCAGGCGCTGTTGCCGGTGCTGTGCCAGGCCCATGGCCTGACCCCGGAGCAGGTGGTGGCCATCGCCAGCAATATTGGTGGCAAGCAGGCGCTGGAGACGGTGCAGGCGCTGTTGCCGGTGCTGTGCCAGGCCCATGGCCTGACCCCGGAGCAGGTGGTGGCCATCGCCAGCCACGATGGCGGCAAGCAGGCGCTGGAGACGGTGCAGCGGCTGTTGCCGGTGCTGTGCCAGGCCCATGGCCTGACCCCGGAGCAGGTGGTGGCCATCGCCAGCCACGATGGCGGCAAGCAGGCGCTGGAGACGGTGCAGCGGCTGTTGCCGGTGCTGTGCCAGGCCCATGGCCTGACCCCGCAGCAGGTGGTGGCCATCGCCAGCAATGGCGGTGGCAAGCAGGCGCTGGAGACGGTGCAGCGGCTGTTGCCGGTGCTGTGCCAGGCCCATGGCCTGACCCCGGAGCAGGTGGTGGCCATCGCCAGCAATAGCGGTGGCAAGCAGGCGCTGGAGACGGTGCAGGCGCTGTTGCCGGTGCTGTGCCAGGCCCATGGCCTGACCCCGGAGCAGGTGGTGGCCATCGCCAGCAATAGCGGTGGCAAGCAGGCGCTGGAGACGGTGCAGCGGCTGTTGCCGGTGCTGTGCCAGGCCCATGGCCTGACCCCGGAGCAGGTGGTGGCCATCGCCAGCCACGATGGCGGCAAGCAGGCGCTGGAGACGGTGCAGCGGCTGTTGCCGGTGCTGTGCCAGGCCCATGGCCTGACCCCGGAGCAGGTGGTGGCCATCGCCAGCCACGATGGCGGCAAGCAGGCGCTGGAGACGGTGCAGCGGCTGTTGCCGGTGCTGTGCCAGGCCCATGGCCTGACCCCGGAGCAGGTGGTGGCCATCGCCAGCCACGATGGCGGCAAGCAGGCGCTGGAGACGGTGCAGCGGCTGTTGCCGGTGCTGTGCCAGGCCCATGGCCTGACCCCGCAGCAGGTGGTGGCCATCGCCAGCAATGGCGGCGGCAGGCCGGCGCTGGAGACGGTGCAGCGGCTGTTGCCGGTGCTGTGCCAGGCCCATGGCCTGACCCCGGAGCAGGTGGTGGCCATCGCCAGCCACGATGGCGGCAAGCAGGCGCTGGAGACGGTGCAGCGGCTGTTGCCGGTGCTGTGCCAGGCCCATGGCCTGACCCCGCAGCAGGTGGTGGCCATCGCCAGCAATGGCGGCGGCAGGCCGGCGCTGGAGAGCATGAGACCCCAGTTATCTCGCCC

**C-terminal module *avrBs3* with stopp (CTRs)**

TTTGGTCTCAAGCATTGTTGCCCAGTTATCTCGCCCTGATCCGGCGTTGGCCGCGTTGACCAACGACCACCTCGTCGCCTTGGCCTGCCTCGGCGGACGTCCTGCGCTGGATGCAGTGAAAAAGGGATTGCCGCACGCGCCGGCCTTGATCAAAAGAACCAATCGCCGTATTCCCGAACGCACATCCCATCGCGTTGCCGACCACGCGCAAGTGGTTCGCGTGCTGGGTTTTTTCCAGTGCCACTCCCACCCAGCGCAAGCATTTGATGACGCCATGACGCAGTTCGGGATGAGCAGGCACGGGTTGTTACAGCTCTTTCGCAGAGTGGGCGTCACCGAACTCGAAGCCCGCAGTGGAACGCTCCCCCCAGCCTCGCAGCGTTGGGACCGTATCCTCCAGGCATCAGGGATGAAAAGGGCCAAACCGTCCCCTACTTCAACTCAAACGCCGGATCAGGCGTCTTTGCATGCATTCGCCGATTCGCTGGAGCGTGACCTTGATGCGCCTAGCCCAATGCACGAGGGAGATCAGACGCGGGCAAGCAGCCGTAAACGGTCCCGATCGGATCGTGCTGTCACCGGTCCCTCCGCACAGCAATCGTTCGAGGTGCGCGTTCCCGAACAGCGCGATGCGCTGCATTTGCCCCTCAGTTGGAGGGTAAAACGCCCGCGTACCAGTATCGGGGGCGGCCTCCCGGATCCTGGTACGCCCACGGCTGCCGACCTGGCAGCGTCCAGCACCGTGATGCGGGAACAAGATGAGGACCCCTTCGCAGGGGCAGCGGATGATTTCCCGGCATTCAACGAAGAGGAGCTCGCATGGTTGATGGAGCTATTGCCTCAGTGAAGCTTTGAGACCAAA

**DNA sequence *c-myc-avrBs3***

overhangs generated by *Bsa*I restriction for directed cloning of AvrBs3 submodules

N-terminal tag (c-Myc/GFP)

N-terminal region (NTR)

Repeat region

C-termnal region

XXX – mutation site (tryptophane(W232X)/RVD1)

XXX – mutation site (arginine (R236G))

XXX - mutation site (arginine (R266G))

**ATG**GGGTTAATTAACGGTGAACAAAAGCTAATCTCCGAGGAAGACTTGAACGGTGAACAAAAATTAATCTCAGAAGAAGACTTGAACGGACTCGACGGTGAACAAAAGTTGATTTCTGAAGAAGATTTGAACGGTGAACAAAAGCTAATCTCCGAGGAAGACTTGAACGGTAGCCATATGGATCCCATTCGTTCGCGCACACCAAGTCCTGCCCGCGAGCTTCTGCCCGGACCCCAACCCGATGGGGTTCAGCCGACTGCAGATCGTGGGGTGTCTCCGCCTGCCGGCGGCCCCCTGGATGGCTTGCCCGCTCGGCGGACGATGTCCCGGACCCGGCTGCCATCTCCCCCTGCCCCCTCACCTGCGTTCTCGGCGGGCAGCTTCAGTGACCTGTTACGTCAGTTCGATCCGTCACTTTTTAATACATCGCTTTTTGATTCATTGCCTCCCTTCGGCGCTCACCATACAGAGGCTGCCACAGGCGAGTGGGATGAGGTGCAATCGGGTCTGCGGGCAGCCGACGCCCCCCCACCCACCATGCGCGTGGCTGTCACTGCCGCGCGGCCGCCGCGCGCCAAGCCGGCGCCGCGACGACGTGCTGCGCAACCCTCCGACGCTTCGCCGGCCGCGCAGGTGGATCTACGCACGCTCGGCTACAGCCAGCAGCAACAGGAGAAGATCAAACCGAAGGTTCGTTCGACAGTGGCGCAGCACCACGAGGCACTGGTCGGCCATGGGTTTACACACGCGCACATCGTTGCGCTCAGCCAACACCCGGCAGCGTTAGGGACCGTCGCTGTCAAGTATCAGGACATGATCGCAGCGTTGCCAGAGGCGACACACGAAGCGATCGTTGGCGTCGGCAAACAGTGGTCCGGCGCACGCGCTCTGGAGGCCTTGCTCACGGTGGCGGGAGAGTTGAGAGGTCCACCGTTACAGTTGGACACAGGCCAACTTCTCAAGATTGCAAAACGTGGCGGCGTGACCGCAGTGGAGGCAGTGCATGCATGGCGCAATGCACTGACGGGTGCCCCCCTGAACCTGACCCCGGAGCAGGTGGTGGCCATCGCCAGCCACGATGGCGGCAAGCAGGCGCTGGAGACGGTGCAGCGGCTGTTGCCGGTGCTGTGCCAGGCCCATGGCCTGACCCCGCAGCAGGTGGTGGCCATCGCCAGCAATGGCGGTGGCAAGCAGGCGCTGGAGACGGTGCAGCGGCTGTTGCCGGTGCTGTGCCAGGCCCATGGCCTGACCCCGCAGCAGGTGGTGGCCATCGCCAGCAATAGCGGTGGCAAGCAGGCGCTGGAGACGGTGCAGCGGCTGTTGCCGGTGCTGTGCCAGGCCCATGGCCTGACCCCGGAGCAGGTGGTGGCCATCGCCAGCAATGGCGGTGGCAAGCAGGCGCTGGAGACGGTGCAGCGGCTGTTGCCGGTGCTGTGCCAGGCCCATGGCCTGACCCCGGAGCAGGTGGTGGCCATCGCCAGCAATATTGGTGGCAAGCAGGCGCTGGAGACGGTGCAGGCGCTGTTGCCGGTGCTGTGCCAGGCCCATGGCCTGACCCCGGAGCAGGTGGTGGCCATCGCCAGCAATATTGGTGGCAAGCAGGCGCTGGAGACGGTGCAGGCGCTGTTGCCGGTGCTGTGCCAGGCCCATGGCCTGACCCCGGAGCAGGTGGTGGCCATCGCCAGCAATATTGGTGGCAAGCAGGCGCTGGAGACGGTGCAGGCGCTGTTGCCGGTGCTGTGCCAGGCCCATGGCCTGACCCCGGAGCAGGTGGTGGCCATCGCCAGCCACGATGGCGGCAAGCAGGCGCTGGAGACGGTGCAGCGGCTGTTGCCGGTGCTGTGCCAGGCCCATGGCCTGACCCCGGAGCAGGTGGTGGCCATCGCCAGCCACGATGGCGGCAAGCAGGCGCTGGAGACGGTGCAGCGGCTGTTGCCGGTGCTGTGCCAGGCCCATGGCCTGACCCCGCAGCAGGTGGTGGCCATCGCCAGCAATGGCGGTGGCAAGCAGGCGCTGGAGACGGTGCAGCGGCTGTTGCCGGTGCTGTGCCAGGCCCATGGCCTGACCCCGGAGCAGGTGGTGGCCATCGCCAGCAATAGCGGTGGCAAGCAGGCGCTGGAGACGGTGCAGGCGCTGTTGCCGGTGCTGTGCCAGGCCCATGGCCTGACCCCGGAGCAGGTGGTGGCCATCGCCAGCAATAGCGGTGGCAAGCAGGCGCTGGAGACGGTGCAGCGGCTGTTGCCGGTGCTGTGCCAGGCCCATGGCCTGACCCCGGAGCAGGTGGTGGCCATCGCCAGCCACGATGGCGGCAAGCAGGCGCTGGAGACGGTGCAGCGGCTGTTGCCGGTGCTGTGCCAGGCCCATGGCCTGACCCCGGAGCAGGTGGTGGCCATCGCCAGCCACGATGGCGGCAAGCAGGCGCTGGAGACGGTGCAGCGGCTGTTGCCGGTGCTGTGCCAGGCCCATGGCCTGACCCCGGAGCAGGTGGTGGCCATCGCCAGCCACGATGGCGGCAAGCAGGCGCTGGAGACGGTGCAGCGGCTGTTGCCGGTGCTGTGCCAGGCCCATGGCCTGACCCCGCAGCAGGTGGTGGCCATCGCCAGCAATGGCGGCGGCAGGCCGGCGCTGGAGACGGTGCAGCGGCTGTTGCCGGTGCTGTGCCAGGCCCATGGCCTGACCCCGGAGCAGGTGGTGGCCATCGCCAGCCACGATGGCGGCAAGCAGGCGCTGGAGACGGTGCAGCGGCTGTTGCCGGTGCTGTGCCAGGCCCATGGCCTGACCCCGCAGCAGGTGGTGGCCATCGCCAGCAATGGCGGCGGCAGGCCGGCGCTGGAGAGCATTGTTGCCCAGTTATCTCGCCCTGATCCGGCGTTGGCCGCGTTGACCAACGACCACCTCGTCGCCTTGGCCTGCCTCGGCGGACGTCCTGCGCTGGATGCAGTGAAAAAGGGATTGCCGCACGCGCCGGCCTTGATCAAAAGAACCAATCGCCGTATTCCCGAACGCACATCCCATCGCGTTGCCGACCACGCGCAAGTGGTTCGCGTGCTGGGTTTTTTCCAGTGCCACTCCCACCCAGCGCAAGCATTTGATGACGCCATGACGCAGTTCGGGATGAGCAGGCACGGGTTGTTACAGCTCTTTCGCAGAGTGGGCGTCACCGAACTCGAAGCCCGCAGTGGAACGCTCCCCCCAGCCTCGCAGCGTTGGGACCGTATCCTCCAGGCATCAGGGATGAAAAGGGCCAAACCGTCCCCTACTTCAACTCAAACGCCGGATCAGGCGTCTTTGCATGCATTCGCCGATTCGCTGGAGCGTGACCTTGATGCGCCTAGCCCAATGCACGAGGGAGATCAGACGCGGGCAAGCAGCCGTAAACGGTCCCGATCGGATCGTGCTGTCACCGGTCCCTCCGCACAGCAATCGTTCGAGGTGCGCGTTCCCGAACAGCGCGATGCGCTGCATTTGCCCCTCAGTTGGAGGGTAAAACGCCCGCGTACCAGTATCGGGGGCGGCCTCCCGGATCCTGGTACGCCCACGGCTGCCGACCTGGCAGCGTCCAGCACCGTGATGCGGGAACAAGATGAGGACCCCTTCGCAGGGGCAGCGGATGATTTCCCGGCATTCAACGAAGAGGAGCTCGCATGGTTGATGGAGCTATTGCCTCAGTGAAGCTT

**Amino acid sequence C-Myc-AvrBs3**

MGLINGEQKLISEEDLNGEQKLISEEDLNGLDGEQKLISEEDLNGEQKLISEEDLNGSHMDPIRSRTPSPARELLPGPQPDGVQPTADRGVSPPAGGPLDGLPARRTMSRTRLPSPPAPSPAFSAGSFSDLLRQFDPSLFNTSLFDSLPPFGAHHTEAATGEWDEVQSGLRAADAPPPTMRVAVTAARPPRAKPAPRRRAAQPSDASPAAQVDLRTLGYSQQQQEKIKPKVRSTVAQHHEALVGHGFTHAHIVALSQHPAALGTVAVKYQDMIAALPEA

-1 THEAIVGVGKQWSGARALEALLTVAGELRGPPLQ

00 LDTGQLLKIAKRGGVTAVEAVHAWRNALTGAPLN

01 LTPEQVVAIASHDGGKQALETVQRLLPVLCQAHG

02 LTPQQVVAIASNGGGKQALETVQRLLPVLCQAHG

03 LTPQQVVAIASNSGGKQALETVQRLLPVLCQAHG

04 LTPEQVVAIASNGGGKQALETVQRLLPVLCQAHG

05 LTPEQVVAIASNIGGKQALETVQALLPVLCQAHG

06 LTPEQVVAIASNIGGKQALETVQALLPVLCQAHG

07 LTPEQVVAIASNIGGKQALETVQALLPVLCQAHG

08 LTPEQVVAIASHDGGKQALETVQRLLPVLCQAHG

09 LTPEQVVAIASHDGGKQALETVQRLLPVLCQAHG

10 LTPQQVVAIASNGGGKQALETVQRLLPVLCQAHG

11 LTPEQVVAIASNSGGKQALETVQALLPVLCQAHG

12 LTPEQVVAIASNSGGKQALETVQRLLPVLCQAHG

13 LTPEQVVAIASHDGGKQALETVQRLLPVLCQAHG

14 LTPEQVVAIASHDGGKQALETVQRLLPVLCQAHG

15 LTPEQVVAIASHDGGKQALETVQRLLPVLCQAHG

16 LTPQQVVAIASNGGGRPALETVQRLLPVLCQAHG

17 LTPEQVVAIASHDGGKQALETVQRLLPVLCQAHG

18 LTPQQVVAIASNGGGRPALESIVA

QLSRPDPALAALTNDHLVALACLGGRPALDAVKKGLPHAPALIKRTNRRIPERTSHRVADHAQVVRVLGFFQCHSHPAQAFDDAMTQFGMSRHGLLQLFRRVGVTELEARSGTLPPASQRWDRILQASGMKRAKPSPTSTQTPDQASLHAFADSLERDLDAPSPMHEGDQTRASSRKRSRSDRAVTGPSAQQSFEVRVPEQRDALHLPLSWRVKRPRTSIGGGLPDPGTPTAADLAASSTVMREQDEDPFAGAADDFPAFNEEELAWLMELLPQ*

**DNA sequence *c-myc-avrBs3-NTR(talC)***

*NTR(talC)*

**ATG**GGGTTAATTAACGGTGAACAAAAGCTAATCTCCGAGGAAGACTTGAACGGTGAACAAAAATTAATCTCAGAAGAAGACTTGAACGGACTCGACGGTGAACAAAAGTTGATTTCTGAAGAAGATTTGAACGGTGAACAAAAGCTAATCTCCGAGGAAGACTTGAACGGTAGCCATATGGATCCCATTCGTCCGCGCGCGCCAAGTCCTGCCCGCGAGGTTCTGCCCGGCCCCCAACCGGATAGGGTTCAGCCGACTGCAGATCGTGGGGTGTCTGCGCCTGCTGGCAGCCCTCTGGATGGCTTGCCCGCTCGGCGGACGATGTCCCGGACCCGGCTGCCATCTCCCCCTGCCCCCTTGCCTGCGTTCTCGGCGGGCAGCTTCAGCGATCTGCTCCGTCAGTTCGATCCGTCGCTTCTTGATACATCGCTTTTTGATTCGATGCCTGCCGTCGGCACGCCTCATACAGAGGCTGCCCCAGCAGAGGGGGATGAGGTGCAATCGGCTCTGCGTGCAGCCGATGACCCGCCACCCACCGTGCGTGTCGCTGTCACTGCCGCGCAGGTGGATCTACGCACGCTCGGCTACAGTCAGCAGCAAGAGAAGATCAAACCGAATGTTCGTTCGACAGTGGCGCAGCACCACGAGGCACTGGTGGGCCATGGGTTTACACACGCGCACATCGTTGCGCTCAGCCGACACCCGGCAGCGTTAGGGACCGTCGCTGTCAAGTATCAGGACATGATCGCGGCGTTACCAGAGGCGACACACGAAGACATCGTTGGGGTCGGCAAACAGTGTTCCGGCGCACGCGCCCTGGAGGCCTTGCTCACGGTGGCGGGAGAGTTGAGAGGTCCACCGTTACAGTTGGACACAGGCCAACTTGTCAAGATTGCAAAACGTGGCGGCGTGACCGCAGTGGAGGCAGTGCATGCATCGCGCAATGCACTGACGGGTGCCCCCCTGAACCTGACCCCGGAGCAGGTGGTGGCCATCGCCAGCCACGATGGCGGCAAGCAGGCGCTGGAGACGGTGCAGCGGCTGTTGCCGGTGCTGTGCCAGGCCCATGGCCTGACCCCGCAGCAGGTGGTGGCCATCGCCAGCAATGGCGGTGGCAAGCAGGCGCTGGAGACGGTGCAGCGGCTGTTGCCGGTGCTGTGCCAGGCCCATGGCCTGACCCCGCAGCAGGTGGTGGCCATCGCCAGCAATAGCGGTGGCAAGCAGGCGCTGGAGACGGTGCAGCGGCTGTTGCCGGTGCTGTGCCAGGCCCATGGCCTGACCCCGGAGCAGGTGGTGGCCATCGCCAGCAATGGCGGTGGCAAGCAGGCGCTGGAGACGGTGCAGCGGCTGTTGCCGGTGCTGTGCCAGGCCCATGGCCTGACCCCGGAGCAGGTGGTGGCCATCGCCAGCAATATTGGTGGCAAGCAGGCGCTGGAGACGGTGCAGGCGCTGTTGCCGGTGCTGTGCCAGGCCCATGGCCTGACCCCGGAGCAGGTGGTGGCCATCGCCAGCAATATTGGTGGCAAGCAGGCGCTGGAGACGGTGCAGGCGCTGTTGCCGGTGCTGTGCCAGGCCCATGGCCTGACCCCGGAGCAGGTGGTGGCCATCGCCAGCAATATTGGTGGCAAGCAGGCGCTGGAGACGGTGCAGGCGCTGTTGCCGGTGCTGTGCCAGGCCCATGGCCTGACCCCGGAGCAGGTGGTGGCCATCGCCAGCCACGATGGCGGCAAGCAGGCGCTGGAGACGGTGCAGCGGCTGTTGCCGGTGCTGTGCCAGGCCCATGGCCTGACCCCGGAGCAGGTGGTGGCCATCGCCAGCCACGATGGCGGCAAGCAGGCGCTGGAGACGGTGCAGCGGCTGTTGCCGGTGCTGTGCCAGGCCCATGGCCTGACCCCGCAGCAGGTGGTGGCCATCGCCAGCAATGGCGGTGGCAAGCAGGCGCTGGAGACGGTGCAGCGGCTGTTGCCGGTGCTGTGCCAGGCCCATGGCCTGACCCCGGAGCAGGTGGTGGCCATCGCCAGCAATAGCGGTGGCAAGCAGGCGCTGGAGACGGTGCAGGCGCTGTTGCCGGTGCTGTGCCAGGCCCATGGCCTGACCCCGGAGCAGGTGGTGGCCATCGCCAGCAATAGCGGTGGCAAGCAGGCGCTGGAGACGGTGCAGCGGCTGTTGCCGGTGCTGTGCCAGGCCCATGGCCTGACCCCGGAGCAGGTGGTGGCCATCGCCAGCCACGATGGCGGCAAGCAGGCGCTGGAGACGGTGCAGCGGCTGTTGCCGGTGCTGTGCCAGGCCCATGGCCTGACCCCGGAGCAGGTGGTGGCCATCGCCAGCCACGATGGCGGCAAGCAGGCGCTGGAGACGGTGCAGCGGCTGTTGCCGGTGCTGTGCCAGGCCCATGGCCTGACCCCGGAGCAGGTGGTGGCCATCGCCAGCCACGATGGCGGCAAGCAGGCGCTGGAGACGGTGCAGCGGCTGTTGCCGGTGCTGTGCCAGGCCCATGGCCTGACCCCGCAGCAGGTGGTGGCCATCGCCAGCAATGGCGGCGGCAGGCCGGCGCTGGAGACGGTGCAGCGGCTGTTGCCGGTGCTGTGCCAGGCCCATGGCCTGACCCCGGAGCAGGTGGTGGCCATCGCCAGCCACGATGGCGGCAAGCAGGCGCTGGAGACGGTGCAGCGGCTGTTGCCGGTGCTGTGCCAGGCCCATGGCCTGACCCCGCAGCAGGTGGTGGCCATCGCCAGCAATGGCGGCGGCAGGCCGGCGCTGGAGAGCATTGTTGCCCAGTTATCTCGCCCTGATCCGGCGTTGGCCGCGTTGACCAACGACCACCTCGTCGCCTTGGCCTGCCTCGGCGGACGTCCTGCGCTGGATGCAGTGAAAAAGGGATTGCCGCACGCGCCGGCCTTGATCAAAAGAACCAATCGCCGTATTCCCGAACGCACATCCCATCGCGTTGCCGACCACGCGCAAGTGGTTCGCGTGCTGGGTTTTTTCCAGTGCCACTCCCACCCAGCGCAAGCATTTGATGACGCCATGACGCAGTTCGGGATGAGCAGGCACGGGTTGTTACAGCTCTTTCGCAGAGTGGGCGTCACCGAACTCGAAGCCCGCAGTGGAACGCTCCCCCCAGCCTCGCAGCGTTGGGACCGTATCCTCCAGGCATCAGGGATGAAAAGGGCCAAACCGTCCCCTACTTCAACTCAAACGCCGGATCAGGCGTCTTTGCATGCATTCGCCGATTCGCTGGAGCGTGACCTTGATGCGCCTAGCCCAATGCACGAGGGAGATCAGACGCGGGCAAGCAGCCGTAAACGGTCCCGATCGGATCGTGCTGTCACCGGTCCCTCCGCACAGCAATCGTTCGAGGTGCGCGTTCCCGAACAGCGCGATGCGCTGCATTTGCCCCTCAGTTGGAGGGTAAAACGCCCGCGTACCAGTATCGGGGGCGGCCTCCCGGATCCTGGTACGCCCACGGCTGCCGACCTGGCAGCGTCCAGCACCGTGATGCGGGAACAAGATGAGGACCCCTTCGCAGGGGCAGCGGATGATTTCCCGGCATTCAACGAAGAGGAGCTCGCATGGTTGATGGAGCTATTGCCTCAGTGAAGCTT

**Amino acid sequence C-Myc-AvrBs3-NTR(TalC)**

MGLINGEQKLISEEDLNGEQKLISEEDLNGLDGEQKLISEEDLNGEQKLISEEDLNGSHMDPIRPRAPSPAREVLPGPQPDRVQPTADRGVSAPAGSPLDGLPARRTMSRTRLPSPPAPLPAFSAGSFSDLLRQFDPSLLDTSLFDSMPAVGTPHTEAAPAEGDEVQSALRAADDPPPTVRVAVTAAQVDLRTLGYSQQQEKIKPNVRSTVAQHHEALVGHGFTHAHIVALSRHPAALGTVAVKYQDMIAALPEA

-1 THEDIVGVGKQCSGARALEALLTVAGELRGPPLQ

00 LDTGQLVKIAKRGGVTAVEAVHASRNALTGAPLN

01 LTPEQVVAIASHDGGKQALETVQRLLPVLCQAHG

02 LTPQQVVAIASNGGGKQALETVQRLLPVLCQAHG

03 LTPQQVVAIASNSGGKQALETVQRLLPVLCQAHG

04 LTPEQVVAIASNGGGKQALETVQRLLPVLCQAHG

05 LTPEQVVAIASNIGGKQALETVQALLPVLCQAHG

06 LTPEQVVAIASNIGGKQALETVQALLPVLCQAHG

07 LTPEQVVAIASNIGGKQALETVQALLPVLCQAHG

08 LTPEQVVAIASHDGGKQALETVQRLLPVLCQAHG

09 LTPEQVVAIASHDGGKQALETVQRLLPVLCQAHG

10 LTPQQVVAIASNGGGKQALETVQRLLPVLCQAHG

11 LTPEQVVAIASNSGGKQALETVQALLPVLCQAHG

12 LTPEQVVAIASNSGGKQALETVQRLLPVLCQAHG

13 LTPEQVVAIASHDGGKQALETVQRLLPVLCQAHG

14 LTPEQVVAIASHDGGKQALETVQRLLPVLCQAHG

15 LTPEQVVAIASHDGGKQALETVQRLLPVLCQAHG

16 LTPQQVVAIASNGGGRPALETVQRLLPVLCQAHG

17 LTPEQVVAIASHDGGKQALETVQRLLPVLCQAHG

18 LTPQQVVAIASNGGGRPALESIVA

QLSRPDPALAALTNDHLVALACLGGRPALDAVKKGLPHAPALIKRTNRRIPERTSHRVADHAQVVRVLGFFQCHSHPAQAFDDAMTQFGMSRHGLLQLFRRVGVTELEARSGTLPPASQRWDRILQASGMKRAKPSPTSTQTPDQASLHAFADSLERDLDAPSPMHEGDQTRASSRKRSRSDRAVTGPSAQQSFEVRVPEQRDALHLPLSWRVKRPRTSIGGGLPDPGTPTAADLAASSTVMREQDEDPFAGAADDFPAFNEEELAWLMELLPQ*

**Nucleotide sequence *c-myc-avrBs3-N1(talC)***

**ATG**GGGTTAATTAACGGTGAACAAAAGCTAATCTCCGAGGAAGACTTGAACGGTGAACAAAAATTAATCTCAGAAGAAGACTTGAACGGACTCGACGGTGAACAAAAGTTGATTTCTGAAGAAGATTTGAACGGTGAACAAAAGCTAATCTCCGAGGAAGACTTGAACGGTAGCCATATGGATCCCATTCGTCCGCGCGCGCCAAGTCCTGCCCGCGAGGTTCTGCCCGGCCCCCAACCGGATAGGGTTCAGCCGACTGCAGATCGTGGGGTGTCTGCGCCTGCTGGCAGCCCTCTGGATGGCTTGCCCGCTCGGCGGACGATGTCCCGGACCCGGCTGCCATCTCCCCCTGCCCCCTTGCCTGCGTTCTCGGCGGGCAGCTTCAGCGATCTGCTCCGTCAGTTCGATCCGTCGCTTCTTGATACATCGCTTTTTGATTCGATGCCTGCCGTCGGCACGCCTCATACAGAGGCTGCCCCAGCAGAGGGGGATGAGGTGCAATCGGCTCTGCGTGCAGCCGATGACCCGCCACCCACCGTGCGTGTCGCTGTCACTGCCGCGCAGGTGGATCTACGCACGCTCGGCTACAGTCAGCAGCAAGAGAAGATCAAACCGAATGTTCGTTCGACAGTGGCGCAGCACCACGAGGCACTGGTGGGCCATGGGTTTACACACGCGCACATCGTTGCGCTCAGCCGACACCCGGCAGCGTTAGGGACCGTCGCTGTCAAGTATCAGGACATGATCGCGGCGTTACCAGAGGCGACACACGAAGACATCGTTGGGGTCGGCAAACAGTGTTCCGGCGCACGCGCCCTGGAGGCCTTGCTCACGGTGGCGGGAGAGTTGAGAGGTCCACCGTTACAGTTGGACACAGGCCAACTTCTCAAGATTGCAAAACGTGGCGGCGTGACCGCAGTGGAGGCAGTGCATGCATGGCGCAATGCACTGACGGGTGCCCCCCTGAACCTGACCCCGGAGCAGGTGGTGGCCATCGCCAGCCACGATGGCGGCAAGCAGGCGCTGGAGACGGTGCAGCGGCTGTTGCCGGTGCTGTGCCAGGCCCATGGCCTGACCCCGCAGCAGGTGGTGGCCATCGCCAGCAATGGCGGTGGCAAGCAGGCGCTGGAGACGGTGCAGCGGCTGTTGCCGGTGCTGTGCCAGGCCCATGGCCTGACCCCGCAGCAGGTGGTGGCCATCGCCAGCAATAGCGGTGGCAAGCAGGCGCTGGAGACGGTGCAGCGGCTGTTGCCGGTGCTGTGCCAGGCCCATGGCCTGACCCCGGAGCAGGTGGTGGCCATCGCCAGCAATGGCGGTGGCAAGCAGGCGCTGGAGACGGTGCAGCGGCTGTTGCCGGTGCTGTGCCAGGCCCATGGCCTGACCCCGGAGCAGGTGGTGGCCATCGCCAGCAATATTGGTGGCAAGCAGGCGCTGGAGACGGTGCAGGCGCTGTTGCCGGTGCTGTGCCAGGCCCATGGCCTGACCCCGGAGCAGGTGGTGGCCATCGCCAGCAATATTGGTGGCAAGCAGGCGCTGGAGACGGTGCAGGCGCTGTTGCCGGTGCTGTGCCAGGCCCATGGCCTGACCCCGGAGCAGGTGGTGGCCATCGCCAGCAATATTGGTGGCAAGCAGGCGCTGGAGACGGTGCAGGCGCTGTTGCCGGTGCTGTGCCAGGCCCATGGCCTGACCCCGGAGCAGGTGGTGGCCATCGCCAGCCACGATGGCGGCAAGCAGGCGCTGGAGACGGTGCAGCGGCTGTTGCCGGTGCTGTGCCAGGCCCATGGCCTGACCCCGGAGCAGGTGGTGGCCATCGCCAGCCACGATGGCGGCAAGCAGGCGCTGGAGACGGTGCAGCGGCTGTTGCCGGTGCTGTGCCAGGCCCATGGCCTGACCCCGCAGCAGGTGGTGGCCATCGCCAGCAATGGCGGTGGCAAGCAGGCGCTGGAGACGGTGCAGCGGCTGTTGCCGGTGCTGTGCCAGGCCCATGGCCTGACCCCGGAGCAGGTGGTGGCCATCGCCAGCAATAGCGGTGGCAAGCAGGCGCTGGAGACGGTGCAGGCGCTGTTGCCGGTGCTGTGCCAGGCCCATGGCCTGACCCCGGAGCAGGTGGTGGCCATCGCCAGCAATAGCGGTGGCAAGCAGGCGCTGGAGACGGTGCAGCGGCTGTTGCCGGTGCTGTGCCAGGCCCATGGCCTGACCCCGGAGCAGGTGGTGGCCATCGCCAGCCACGATGGCGGCAAGCAGGCGCTGGAGACGGTGCAGCGGCTGTTGCCGGTGCTGTGCCAGGCCCATGGCCTGACCCCGGAGCAGGTGGTGGCCATCGCCAGCCACGATGGCGGCAAGCAGGCGCTGGAGACGGTGCAGCGGCTGTTGCCGGTGCTGTGCCAGGCCCATGGCCTGACCCCGGAGCAGGTGGTGGCCATCGCCAGCCACGATGGCGGCAAGCAGGCGCTGGAGACGGTGCAGCGGCTGTTGCCGGTGCTGTGCCAGGCCCATGGCCTGACCCCGCAGCAGGTGGTGGCCATCGCCAGCAATGGCGGCGGCAGGCCGGCGCTGGAGACGGTGCAGCGGCTGTTGCCGGTGCTGTGCCAGGCCCATGGCCTGACCCCGGAGCAGGTGGTGGCCATCGCCAGCCACGATGGCGGCAAGCAGGCGCTGGAGACGGTGCAGCGGCTGTTGCCGGTGCTGTGCCAGGCCCATGGCCTGACCCCGCAGCAGGTGGTGGCCATCGCCAGCAATGGCGGCGGCAGGCCGGCGCTGGAGAGCATTGTTGCCCAGTTATCTCGCCCTGATCCGGCGTTGGCCGCGTTGACCAACGACCACCTCGTCGCCTTGGCCTGCCTCGGCGGACGTCCTGCGCTGGATGCAGTGAAAAAGGGATTGCCGCACGCGCCGGCCTTGATCAAAAGAACCAATCGCCGTATTCCCGAACGCACATCCCATCGCGTTGCCGACCACGCGCAAGTGGTTCGCGTGCTGGGTTTTTTCCAGTGCCACTCCCACCCAGCGCAAGCATTTGATGACGCCATGACGCAGTTCGGGATGAGCAGGCACGGGTTGTTACAGCTCTTTCGCAGAGTGGGCGTCACCGAACTCGAAGCCCGCAGTGGAACGCTCCCCCCAGCCTCGCAGCGTTGGGACCGTATCCTCCAGGCATCAGGGATGAAAAGGGCCAAACCGTCCCCTACTTCAACTCAAACGCCGGATCAGGCGTCTTTGCATGCATTCGCCGATTCGCTGGAGCGTGACCTTGATGCGCCTAGCCCAATGCACGAGGGAGATCAGACGCGGGCAAGCAGCCGTAAACGGTCCCGATCGGATCGTGCTGTCACCGGTCCCTCCGCACAGCAATCGTTCGAGGTGCGCGTTCCCGAACAGCGCGATGCGCTGCATTTGCCCCTCAGTTGGAGGGTAAAACGCCCGCGTACCAGTATCGGGGGCGGCCTCCCGGATCCTGGTACGCCCACGGCTGCCGACCTGGCAGCGTCCAGCACCGTGATGCGGGAACAAGATGAGGACCCCTTCGCAGGGGCAGCGGATGATTTCCCGGCATTCAACGAAGAGGAGCTCGCATGGTTGATGGAGCTATTGCCTCAGTGAAGCTT

**Amino acid sequence C-Myc-AvrBs3-N1(TalC)**

MGLINGEQKLISEEDLNGEQKLISEEDLNGLDGEQKLISEEDLNGEQKLISEEDLNGSHMDPIRPRAPSPAREVLPGPQPDRVQPTADRGVSAPAGSPLDGLPARRTMSRTRLPSPPAPLPAFSAGSFSDLLRQFDPSLLDTSLFDSMPAVGTPHTEAAPAEGDEVQSALRAADDPPPTVRVAVTAAQVDLRTLGYSQQQEKIKPNVRSTVAQHHEALVGHGFTHAHIVALSRHPAALGTVAVKYQDMIAALPEA

-1 THEDIVGVGKQCSGARALEALLTVAGELRGPPLQ

00 LDTGQLLKIAKRGGVTAVEAVHAWRNALTGAPLN

01 LTPEQVVAIASHDGGKQALETVQRLLPVLCQAHG

02 LTPQQVVAIASNGGGKQALETVQRLLPVLCQAHG

03 LTPQQVVAIASNSGGKQALETVQRLLPVLCQAHG

04 LTPEQVVAIASNGGGKQALETVQRLLPVLCQAHG

05 LTPEQVVAIASNIGGKQALETVQALLPVLCQAHG

06 LTPEQVVAIASNIGGKQALETVQALLPVLCQAHG

07 LTPEQVVAIASNIGGKQALETVQALLPVLCQAHG

08 LTPEQVVAIASHDGGKQALETVQRLLPVLCQAHG

09 LTPEQVVAIASHDGGKQALETVQRLLPVLCQAHG

10 LTPQQVVAIASNGGGKQALETVQRLLPVLCQAHG

11 LTPEQVVAIASNSGGKQALETVQALLPVLCQAHG

12 LTPEQVVAIASNSGGKQALETVQRLLPVLCQAHG

13 LTPEQVVAIASHDGGKQALETVQRLLPVLCQAHG

14 LTPEQVVAIASHDGGKQALETVQRLLPVLCQAHG

15 LTPEQVVAIASHDGGKQALETVQRLLPVLCQAHG

16 LTPQQVVAIASNGGGRPALETVQRLLPVLCQAHG

17 LTPEQVVAIASHDGGKQALETVQRLLPVLCQAHG

18 LTPQQVVAIASNGGGRPALESIVA

QLSRPDPALAALTNDHLVALACLGGRPALDAVKKGLPHAPALIKRTNRRIPERTSHRVADHAQVVRVLGFFQCHSHPAQAFDDAMTQFGMSRHGLLQLFRRVGVTELEARSGTLPPASQRWDRILQASGMKRAKPSPTSTQTPDQASLHAFADSLERDLDAPSPMHEGDQTRASSRKRSRSDRAVTGPSAQQSFEVRVPEQRDALHLPLSWRVKRPRTSIGGGLPDPGTPTAADLAASSTVMREQDEDPFAGAADDFPAFNEEELAWLMELLPQ*

**DNA sequence *c-myc-avrBs3-N2(talC)***

**ATG**GGGTTAATTAACGGTGAACAAAAGCTAATCTCCGAGGAAGACTTGAACGGTGAACAAAAATTAATCTCAGAAGAAGACTTGAACGGACTCGACGGTGAACAAAAGTTGATTTCTGAAGAAGATTTGAACGGTGAACAAAAGCTAATCTCCGAGGAAGACTTGAACGGTAGCCAT**ATG**GATCCCATTCGTCCGCGCGCGCCAAGTCCTGCCCGCGAGGTTCTGCCCGGCCCCCAACCGGATAGGGTTCAGCCGACTGCAGATCGTGGGGTGTCTGCGCCTGCTGGCAGCCCTCTGGATGGCTTGCCCGCTCGGCGGACGATGTCCCGGACCCGGCTGCCATCTCCCCCTGCCCCCTTGCCTGCGTTCTCGGCGGGCAGCTTCAGCGATCTGCTCCGTCAGTTCGATCCGTCGCTTCTTGATACATCGCTTTTTGATTCGATGCCTGCCGTCGGCACGCCTCATACAGAGGCTGCCCCAGCAGAGGGGGATGAGGTGCAATCGGCTCTGCGTGCAGCCGATGACCCGCCACCCACCGTGCGTGTCGCTGTCACTGCCGCGCAGGTGGATCTACGCACGCTCGGCTACAGTCAGCAGCAAGAGAAGATCAAACCGAATGTTCGTTCGACAGTGGCGCAGCACCACGAGGCACTGGTGGGCCATGGGTTTACACACGCGCACATCGTTGCGCTCAGCCGACACCCGGCAGCGTTAGGGACCGTCGCTGTCAAGTATCAGGACATGATCGCAGCGTTGCCAGAGGCGACACACGAAGCGATCGTTGGCGTCGGCAAACAGTGGTCCGGCGCACGCGCTCTGGAGGCCTTGCTCACGGTGGCGGGAGAGTTGAGAGGTCCACCGTTACAGTTGGACACAGGCCAACTTCTCAAGATTGCAAAACGTGGCGGCGTGACCGCAGTGGAGGCAGTGCATGCATGGCGCAATGCACTGACGGGTGCCCCCCTGAACCTGACCCCGGAGCAGGTGGTGGCCATCGCCAGCCACGATGGCGGCAAGCAGGCGCTGGAGACGGTGCAGCGGCTGTTGCCGGTGCTGTGCCAGGCCCATGGCCTGACCCCGCAGCAGGTGGTGGCCATCGCCAGCAATGGCGGTGGCAAGCAGGCGCTGGAGACGGTGCAGCGGCTGTTGCCGGTGCTGTGCCAGGCCCATGGCCTGACCCCGCAGCAGGTGGTGGCCATCGCCAGCAATAGCGGTGGCAAGCAGGCGCTGGAGACGGTGCAGCGGCTGTTGCCGGTGCTGTGCCAGGCCCATGGCCTGACCCCGGAGCAGGTGGTGGCCATCGCCAGCAATGGCGGTGGCAAGCAGGCGCTGGAGACGGTGCAGCGGCTGTTGCCGGTGCTGTGCCAGGCCCATGGCCTGACCCCGGAGCAGGTGGTGGCCATCGCCAGCAATATTGGTGGCAAGCAGGCGCTGGAGACGGTGCAGGCGCTGTTGCCGGTGCTGTGCCAGGCCCATGGCCTGACCCCGGAGCAGGTGGTGGCCATCGCCAGCAATATTGGTGGCAAGCAGGCGCTGGAGACGGTGCAGGCGCTGTTGCCGGTGCTGTGCCAGGCCCATGGCCTGACCCCGGAGCAGGTGGTGGCCATCGCCAGCAATATTGGTGGCAAGCAGGCGCTGGAGACGGTGCAGGCGCTGTTGCCGGTGCTGTGCCAGGCCCATGGCCTGACCCCGGAGCAGGTGGTGGCCATCGCCAGCCACGATGGCGGCAAGCAGGCGCTGGAGACGGTGCAGCGGCTGTTGCCGGTGCTGTGCCAGGCCCATGGCCTGACCCCGGAGCAGGTGGTGGCCATCGCCAGCCACGATGGCGGCAAGCAGGCGCTGGAGACGGTGCAGCGGCTGTTGCCGGTGCTGTGCCAGGCCCATGGCCTGACCCCGCAGCAGGTGGTGGCCATCGCCAGCAATGGCGGTGGCAAGCAGGCGCTGGAGACGGTGCAGCGGCTGTTGCCGGTGCTGTGCCAGGCCCATGGCCTGACCCCGGAGCAGGTGGTGGCCATCGCCAGCAATAGCGGTGGCAAGCAGGCGCTGGAGACGGTGCAGGCGCTGTTGCCGGTGCTGTGCCAGGCCCATGGCCTGACCCCGGAGCAGGTGGTGGCCATCGCCAGCAATAGCGGTGGCAAGCAGGCGCTGGAGACGGTGCAGCGGCTGTTGCCGGTGCTGTGCCAGGCCCATGGCCTGACCCCGGAGCAGGTGGTGGCCATCGCCAGCCACGATGGCGGCAAGCAGGCGCTGGAGACGGTGCAGCGGCTGTTGCCGGTGCTGTGCCAGGCCCATGGCCTGACCCCGGAGCAGGTGGTGGCCATCGCCAGCCACGATGGCGGCAAGCAGGCGCTGGAGACGGTGCAGCGGCTGTTGCCGGTGCTGTGCCAGGCCCATGGCCTGACCCCGGAGCAGGTGGTGGCCATCGCCAGCCACGATGGCGGCAAGCAGGCGCTGGAGACGGTGCAGCGGCTGTTGCCGGTGCTGTGCCAGGCCCATGGCCTGACCCCGCAGCAGGTGGTGGCCATCGCCAGCAATGGCGGCGGCAGGCCGGCGCTGGAGACGGTGCAGCGGCTGTTGCCGGTGCTGTGCCAGGCCCATGGCCTGACCCCGGAGCAGGTGGTGGCCATCGCCAGCCACGATGGCGGCAAGCAGGCGCTGGAGACGGTGCAGCGGCTGTTGCCGGTGCTGTGCCAGGCCCATGGCCTGACCCCGCAGCAGGTGGTGGCCATCGCCAGCAATGGCGGCGGCAGGCCGGCGCTGGAGAGCATTGTTGCCCAGTTATCTCGCCCTGATCCGGCGTTGGCCGCGTTGACCAACGACCACCTCGTCGCCTTGGCCTGCCTCGGCGGACGTCCTGCGCTGGATGCAGTGAAAAAGGGATTGCCGCACGCGCCGGCCTTGATCAAAAGAACCAATCGCCGTATTCCCGAACGCACATCCCATCGCGTTGCCGACCACGCGCAAGTGGTTCGCGTGCTGGGTTTTTTCCAGTGCCACTCCCACCCAGCGCAAGCATTTGATGACGCCATGACGCAGTTCGGGATGAGCAGGCACGGGTTGTTACAGCTCTTTCGCAGAGTGGGCGTCACCGAACTCGAAGCCCGCAGTGGAACGCTCCCCCCAGCCTCGCAGCGTTGGGACCGTATCCTCCAGGCATCAGGGATGAAAAGGGCCAAACCGTCCCCTACTTCAACTCAAACGCCGGATCAGGCGTCTTTGCATGCATTCGCCGATTCGCTGGAGCGTGACCTTGATGCGCCTAGCCCAATGCACGAGGGAGATCAGACGCGGGCAAGCAGCCGTAAACGGTCCCGATCGGATCGTGCTGTCACCGGTCCCTCCGCACAGCAATCGTTCGAGGTGCGCGTTCCCGAACAGCGCGATGCGCTGCATTTGCCCCTCAGTTGGAGGGTAAAACGCCCGCGTACCAGTATCGGGGGCGGCCTCCCGGATCCTGGTACGCCCACGGCTGCCGACCTGGCAGCGTCCAGCACCGTGATGCGGGAACAAGATGAGGACCCCTTCGCAGGGGCAGCGGATGATTTCCCGGCATTCAACGAAGAGGAGCTCGCATGGTTGATGGAGCTATTGCCTCAGTGAAGCTT

**Amino acid sequence c-Myc-AvrBs3-N2(TalC)**

MGLINGEQKLISEEDLNGEQKLISEEDLNGLDGEQKLISEEDLNGEQKLISEEDLNGSHMDPIRPRAPSPAREVLPGPQPDRVQPTADRGVSAPAGSPLDGLPARRTMSRTRLPSPPAPLPAFSAGSFSDLLRQFDPSLLDTSLFDSMPAVGTPHTEAAPAEGDEVQSALRAADDPPPTVRVAVTAAQVDLRTLGYSQQQEKIKPNVRSTVAQHHEALVGHGFTHAHIVALSRHPAALGTVAVKYQDMIAALPEA

-1 THEAIVGVGKQWSGARALEALLTVAGELRGPPLQ

00 LDTGQLLKIAKRGGVTAVEAVHAWRNALTGAPLN

01 LTPEQVVAIASHDGGKQALETVQRLLPVLCQAHG

02 LTPQQVVAIASNGGGKQALETVQRLLPVLCQAHG

03 LTPQQVVAIASNSGGKQALETVQRLLPVLCQAHG

04 LTPEQVVAIASNGGGKQALETVQRLLPVLCQAHG

05 LTPEQVVAIASNIGGKQALETVQALLPVLCQAHG

06 LTPEQVVAIASNIGGKQALETVQALLPVLCQAHG

07 LTPEQVVAIASNIGGKQALETVQALLPVLCQAHG

08 LTPEQVVAIASHDGGKQALETVQRLLPVLCQAHG

09 LTPEQVVAIASHDGGKQALETVQRLLPVLCQAHG

10 LTPQQVVAIASNGGGKQALETVQRLLPVLCQAHG

11 LTPEQVVAIASNSGGKQALETVQALLPVLCQAHG

12 LTPEQVVAIASNSGGKQALETVQRLLPVLCQAHG

13 LTPEQVVAIASHDGGKQALETVQRLLPVLCQAHG

14 LTPEQVVAIASHDGGKQALETVQRLLPVLCQAHG

15 LTPEQVVAIASHDGGKQALETVQRLLPVLCQAHG

16 LTPQQVVAIASNGGGRPALETVQRLLPVLCQAHG

17 LTPEQVVAIASHDGGKQALETVQRLLPVLCQAHG

18 LTPQQVVAIASNGGGRPALESIVA

QLSRPDPALAALTNDHLVALACLGGRPALDAVKKGLPHAPALIKRTNRRIPERTSHRVADHAQVVRVLGFFQCHSHPAQAFDDAMTQFGMSRHGLLQLFRRVGVTELEARSGTLPPASQRWDRILQASGMKRAKPSPTSTQTPDQASLHAFADSLERDLDAPSPMHEGDQTRASSRKRSRSDRAVTGPSAQQSFEVRVPEQRDALHLPLSWRVKRPRTSIGGGLPDPGTPTAADLAASSTVMREQDEDPFAGAADDFPAFNEEELAWLMELLPQ*

**DNA sequence *c-myc-avrBs3-N3(talC)***

**ATG**GGGTTAATTAACGGTGAACAAAAGCTAATCTCCGAGGAAGACTTGAACGGTGAACAAAAATTAATCTCAGAAGAAGACTTGAACGGACTCGACGGTGAACAAAAGTTGATTTCTGAAGAAGATTTGAACGGTGAACAAAAGCTAATCTCCGAGGAAGACTTGAACGGTAGCCATATGGATCCCATTCGTTCGCGCACACCAAGTCCTGCCCGCGAGCTTCTGCCCGGACCCCAACCCGATGGGGTTCAGCCGACTGCAGATCGTGGGGTGTCTCCGCCTGCCGGCGGCCCCCTGGATGGCTTGCCCGCTCGGCGGACGATGTCCCGGACCCGGCTGCCATCTCCCCCTGCCCCCTCACCTGCGTTCTCGGCGGGCAGCTTCAGTGACCTGTTACGTCAGTTCGATCCGTCACTTTTTAATACATCGCTTTTTGATTCATTGCCTCCCTTCGGCGCTCACCATACAGAGGCTGCCACAGGCGAGTGGGATGAGGTGCAATCGGGTCTGCGGGCAGCCGACGCCCCCCCACCCACCATGCGCGTGGCTGTCACTGCCGCGCGGCCGCCGCGCGCCAAGCCGGCGCCGCGACGACGTGCTGCGCAACCCTCCGACGCTTCGCCGGCCGCGCAGGTGGATCTACGCACGCTCGGCTACAGCCAGCAGCAACAGGAGAAGATCAAACCGAAGGTTCGTTCGACAGTGGCGCAGCACCACGAGGCACTGGTCGGCCATGGGTTTACACACGCGCACATCGTTGCGCTCAGCCAACACCCGGCAGCGTTAGGGACCGTCGCTGTCAAGTATCAGGACATGATCGCGGCGTTACCAGAGGCGACACACGAAGACATCGTTGGGGTCGGCAAACAGTGTTCCGGCGCACGCGCCCTGGAGGCCTTGCTCACGGTGGCGGGAGAGTTGAGAGGTCCACCGTTACAGTTGGACACAGGCCAACTTGTCAAGATTGCAAAACGTGGCGGCGTGACCGCAGTGGAGGCAGTGCATGCATCGCGCAATGCACTGACGGGTGCCCCCCTGAACCTGACCCCGGAGCAGGTGGTGGCCATCGCCAGCCACGATGGCGGCAAGCAGGCGCTGGAGACGGTGCAGCGGCTGTTGCCGGTGCTGTGCCAGGCCCATGGCCTGACCCCGCAGCAGGTGGTGGCCATCGCCAGCAATGGCGGTGGCAAGCAGGCGCTGGAGACGGTGCAGCGGCTGTTGCCGGTGCTGTGCCAGGCCCATGGCCTGACCCCGCAGCAGGTGGTGGCCATCGCCAGCAATAGCGGTGGCAAGCAGGCGCTGGAGACGGTGCAGCGGCTGTTGCCGGTGCTGTGCCAGGCCCATGGCCTGACCCCGGAGCAGGTGGTGGCCATCGCCAGCAATGGCGGTGGCAAGCAGGCGCTGGAGACGGTGCAGCGGCTGTTGCCGGTGCTGTGCCAGGCCCATGGCCTGACCCCGGAGCAGGTGGTGGCCATCGCCAGCAATATTGGTGGCAAGCAGGCGCTGGAGACGGTGCAGGCGCTGTTGCCGGTGCTGTGCCAGGCCCATGGCCTGACCCCGGAGCAGGTGGTGGCCATCGCCAGCAATATTGGTGGCAAGCAGGCGCTGGAGACGGTGCAGGCGCTGTTGCCGGTGCTGTGCCAGGCCCATGGCCTGACCCCGGAGCAGGTGGTGGCCATCGCCAGCAATATTGGTGGCAAGCAGGCGCTGGAGACGGTGCAGGCGCTGTTGCCGGTGCTGTGCCAGGCCCATGGCCTGACCCCGGAGCAGGTGGTGGCCATCGCCAGCCACGATGGCGGCAAGCAGGCGCTGGAGACGGTGCAGCGGCTGTTGCCGGTGCTGTGCCAGGCCCATGGCCTGACCCCGGAGCAGGTGGTGGCCATCGCCAGCCACGATGGCGGCAAGCAGGCGCTGGAGACGGTGCAGCGGCTGTTGCCGGTGCTGTGCCAGGCCCATGGCCTGACCCCGCAGCAGGTGGTGGCCATCGCCAGCAATGGCGGTGGCAAGCAGGCGCTGGAGACGGTGCAGCGGCTGTTGCCGGTGCTGTGCCAGGCCCATGGCCTGACCCCGGAGCAGGTGGTGGCCATCGCCAGCAATAGCGGTGGCAAGCAGGCGCTGGAGACGGTGCAGGCGCTGTTGCCGGTGCTGTGCCAGGCCCATGGCCTGACCCCGGAGCAGGTGGTGGCCATCGCCAGCAATAGCGGTGGCAAGCAGGCGCTGGAGACGGTGCAGCGGCTGTTGCCGGTGCTGTGCCAGGCCCATGGCCTGACCCCGGAGCAGGTGGTGGCCATCGCCAGCCACGATGGCGGCAAGCAGGCGCTGGAGACGGTGCAGCGGCTGTTGCCGGTGCTGTGCCAGGCCCATGGCCTGACCCCGGAGCAGGTGGTGGCCATCGCCAGCCACGATGGCGGCAAGCAGGCGCTGGAGACGGTGCAGCGGCTGTTGCCGGTGCTGTGCCAGGCCCATGGCCTGACCCCGGAGCAGGTGGTGGCCATCGCCAGCCACGATGGCGGCAAGCAGGCGCTGGAGACGGTGCAGCGGCTGTTGCCGGTGCTGTGCCAGGCCCATGGCCTGACCCCGCAGCAGGTGGTGGCCATCGCCAGCAATGGCGGCGGCAGGCCGGCGCTGGAGACGGTGCAGCGGCTGTTGCCGGTGCTGTGCCAGGCCCATGGCCTGACCCCGGAGCAGGTGGTGGCCATCGCCAGCCACGATGGCGGCAAGCAGGCGCTGGAGACGGTGCAGCGGCTGTTGCCGGTGCTGTGCCAGGCCCATGGCCTGACCCCGCAGCAGGTGGTGGCCATCGCCAGCAATGGCGGCGGCAGGCCGGCGCTGGAGAGCATTGTTGCCCAGTTATCTCGCCCTGATCCGGCGTTGGCCGCGTTGACCAACGACCACCTCGTCGCCTTGGCCTGCCTCGGCGGACGTCCTGCGCTGGATGCAGTGAAAAAGGGATTGCCGCACGCGCCGGCCTTGATCAAAAGAACCAATCGCCGTATTCCCGAACGCACATCCCATCGCGTTGCCGACCACGCGCAAGTGGTTCGCGTGCTGGGTTTTTTCCAGTGCCACTCCCACCCAGCGCAAGCATTTGATGACGCCATGACGCAGTTCGGGATGAGCAGGCACGGGTTGTTACAGCTCTTTCGCAGAGTGGGCGTCACCGAACTCGAAGCCCGCAGTGGAACGCTCCCCCCAGCCTCGCAGCGTTGGGACCGTATCCTCCAGGCATCAGGGATGAAAAGGGCCAAACCGTCCCCTACTTCAACTCAAACGCCGGATCAGGCGTCTTTGCATGCATTCGCCGATTCGCTGGAGCGTGACCTTGATGCGCCTAGCCCAATGCACGAGGGAGATCAGACGCGGGCAAGCAGCCGTAAACGGTCCCGATCGGATCGTGCTGTCACCGGTCCCTCCGCACAGCAATCGTTCGAGGTGCGCGTTCCCGAACAGCGCGATGCGCTGCATTTGCCCCTCAGTTGGAGGGTAAAACGCCCGCGTACCAGTATCGGGGGCGGCCTCCCGGATCCTGGTACGCCCACGGCTGCCGACCTGGCAGCGTCCAGCACCGTGATGCGGGAACAAGATGAGGACCCCTTCGCAGGGGCAGCGGATGATTTCCCGGCATTCAACGAAGAGGAGCTCGCATGGTTGATGGAGCTATTGCCTCAGTGAAGCTT

**Amino acid sequence c-Myc-AvrBs3-N3(TalC)**

MGLINGEQKLISEEDLNGEQKLISEEDLNGLDGEQKLISEEDLNGEQKLISEEDLNGSHMDPIRSRTPSPARELLPGPQPDGVQPTADRGVSPPAGGPLDGLPARRTMSRTRLPSPPAPSPAFSAGSFSDLLRQFDPSLFNTSLFDSLPPFGAHHTEAATGEWDEVQSGLRAADAPPPTMRVAVTAARPPRAKPAPRRRAAQPSDASPAAQVDLRTLGYSQQQQEKIKPKVRSTVAQHHEALVGHGFTHAHIVALSQHPAALGTVAVKYQDMIAALPEA

-1 THEDIVGVGKQCSGARALEALLTVAGELRGPPLQ

00 LDTGQLVKIAKRGGVTAVEAVHASRNALTGAPLN

01 LTPEQVVAIASHDGGKQALETVQRLLPVLCQAHG

02 LTPQQVVAIASNGGGKQALETVQRLLPVLCQAHG

03 LTPQQVVAIASNSGGKQALETVQRLLPVLCQAHG

04 LTPEQVVAIASNGGGKQALETVQRLLPVLCQAHG

05 LTPEQVVAIASNIGGKQALETVQALLPVLCQAHG

06 LTPEQVVAIASNIGGKQALETVQALLPVLCQAHG

07 LTPEQVVAIASNIGGKQALETVQALLPVLCQAHG

08 LTPEQVVAIASHDGGKQALETVQRLLPVLCQAHG

09 LTPEQVVAIASHDGGKQALETVQRLLPVLCQAHG

10 LTPQQVVAIASNGGGKQALETVQRLLPVLCQAHG

11 LTPEQVVAIASNSGGKQALETVQALLPVLCQAHG

12 LTPEQVVAIASNSGGKQALETVQRLLPVLCQAHG

13 LTPEQVVAIASHDGGKQALETVQRLLPVLCQAHG

14 LTPEQVVAIASHDGGKQALETVQRLLPVLCQAHG

15 LTPEQVVAIASHDGGKQALETVQRLLPVLCQAHG

16 LTPQQVVAIASNGGGRPALETVQRLLPVLCQAHG

17 LTPEQVVAIASHDGGKQALETVQRLLPVLCQAHG

18 LTPQQVVAIASNGGGRPALESIVA

QLSRPDPALAALTNDHLVALACLGGRPALDAVKKGLPHAPALIKRTNRRIPERTSHRVADHAQVVRVLGFFQCHSHPAQAFDDAMTQFGMSRHGLLQLFRRVGVTELEARSGTLPPASQRWDRILQASGMKRAKPSPTSTQTPDQASLHAFADSLERDLDAPSPMHEGDQTRASSRKRSRSDRAVTGPSAQQSFEVRVPEQRDALHLPLSWRVKRPRTSIGGGLPDPGTPTAADLAASSTVMREQDEDPFAGAADDFPAFNEEELAWLMELLPQ*

**DNA sequence *c-myc-avrBs3-N4(talC)***

**ATG**GGGTTAATTAACGGTGAACAAAAGCTAATCTCCGAGGAAGACTTGAACGGTGAACAAAAATTAATCTCAGAAGAAGACTTGAACGGACTCGACGGTGAACAAAAGTTGATTTCTGAAGAAGATTTGAACGGTGAACAAAAGCTAATCTCCGAGGAAGACTTGAACGGTAGCCATATGGATCCCATTCGTTCGCGCACACCAAGTCCTGCCCGCGAGCTTCTGCCCGGACCCCAACCCGATGGGGTTCAGCCGACTGCAGATCGTGGGGTGTCTCCGCCTGCCGGCGGCCCCCTGGATGGCTTGCCCGCTCGGCGGACGATGTCCCGGACCCGGCTGCCATCTCCCCCTGCCCCCTCACCTGCGTTCTCGGCGGGCAGCTTCAGTGACCTGTTACGTCAGTTCGATCCGTCACTTTTTAATACATCGCTTTTTGATTCATTGCCTCCCTTCGGCGCTCACCATACAGAGGCTGCCACAGGCGAGTGGGATGAGGTGCAATCGGGTCTGCGGGCAGCCGACGCCCCCCCACCCACCATGCGCGTGGCTGTCACTGCCGCGCGGCCGCCGCGCGCCAAGCCGGCGCCGCGACGACGTGCTGCGCAACCCTCCGACGCTTCGCCGGCCGCGCAGGTGGATCTACGCACGCTCGGCTACAGCCAGCAGCAACAGGAGAAGATCAAACCGAAGGTTCGTTCGACAGTGGCGCAGCACCACGAGGCACTGGTCGGCCATGGGTTTACACACGCGCACATCGTTGCGCTCAGCCAACACCCGGCAGCGTTAGGGACCGTCGCTGTCAAGTATCAGGACATGATCGCAGCGTTGCCAGAGGCGACACACGAAGCGATCGTTGGCGTCGGCAAACAGTGCTCCGGCGCACGCGCTCTGGAGGCCTTGCTCACGGTGGCGGGAGAGTTGAGAGGTCCACCGTTACAGTTGGACACAGGCCAACTTGTCAAGATTGCAAAACGTGGCGGCGTGACCGCAGTGGAGGCAGTGCATGCATCGCGCAATGCACTGACGGGTGCCCCCCTGAACCTGACCCCGGAGCAGGTGGTGGCCATCGCCAGCCACGATGGCGGCAAGCAGGCGCTGGAGACGGTGCAGCGGCTGTTGCCGGTGCTGTGCCAGGCCCATGGCCTGACCCCGCAGCAGGTGGTGGCCATCGCCAGCAATGGCGGTGGCAAGCAGGCGCTGGAGACGGTGCAGCGGCTGTTGCCGGTGCTGTGCCAGGCCCATGGCCTGACCCCGCAGCAGGTGGTGGCCATCGCCAGCAATAGCGGTGGCAAGCAGGCGCTGGAGACGGTGCAGCGGCTGTTGCCGGTGCTGTGCCAGGCCCATGGCCTGACCCCGGAGCAGGTGGTGGCCATCGCCAGCAATGGCGGTGGCAAGCAGGCGCTGGAGACGGTGCAGCGGCTGTTGCCGGTGCTGTGCCAGGCCCATGGCCTGACCCCGGAGCAGGTGGTGGCCATCGCCAGCAATATTGGTGGCAAGCAGGCGCTGGAGACGGTGCAGGCGCTGTTGCCGGTGCTGTGCCAGGCCCATGGCCTGACCCCGGAGCAGGTGGTGGCCATCGCCAGCAATATTGGTGGCAAGCAGGCGCTGGAGACGGTGCAGGCGCTGTTGCCGGTGCTGTGCCAGGCCCATGGCCTGACCCCGGAGCAGGTGGTGGCCATCGCCAGCAATATTGGTGGCAAGCAGGCGCTGGAGACGGTGCAGGCGCTGTTGCCGGTGCTGTGCCAGGCCCATGGCCTGACCCCGGAGCAGGTGGTGGCCATCGCCAGCCACGATGGCGGCAAGCAGGCGCTGGAGACGGTGCAGCGGCTGTTGCCGGTGCTGTGCCAGGCCCATGGCCTGACCCCGGAGCAGGTGGTGGCCATCGCCAGCCACGATGGCGGCAAGCAGGCGCTGGAGACGGTGCAGCGGCTGTTGCCGGTGCTGTGCCAGGCCCATGGCCTGACCCCGCAGCAGGTGGTGGCCATCGCCAGCAATGGCGGTGGCAAGCAGGCGCTGGAGACGGTGCAGCGGCTGTTGCCGGTGCTGTGCCAGGCCCATGGCCTGACCCCGGAGCAGGTGGTGGCCATCGCCAGCAATAGCGGTGGCAAGCAGGCGCTGGAGACGGTGCAGGCGCTGTTGCCGGTGCTGTGCCAGGCCCATGGCCTGACCCCGGAGCAGGTGGTGGCCATCGCCAGCAATAGCGGTGGCAAGCAGGCGCTGGAGACGGTGCAGCGGCTGTTGCCGGTGCTGTGCCAGGCCCATGGCCTGACCCCGGAGCAGGTGGTGGCCATCGCCAGCCACGATGGCGGCAAGCAGGCGCTGGAGACGGTGCAGCGGCTGTTGCCGGTGCTGTGCCAGGCCCATGGCCTGACCCCGGAGCAGGTGGTGGCCATCGCCAGCCACGATGGCGGCAAGCAGGCGCTGGAGACGGTGCAGCGGCTGTTGCCGGTGCTGTGCCAGGCCCATGGCCTGACCCCGGAGCAGGTGGTGGCCATCGCCAGCCACGATGGCGGCAAGCAGGCGCTGGAGACGGTGCAGCGGCTGTTGCCGGTGCTGTGCCAGGCCCATGGCCTGACCCCGCAGCAGGTGGTGGCCATCGCCAGCAATGGCGGCGGCAGGCCGGCGCTGGAGACGGTGCAGCGGCTGTTGCCGGTGCTGTGCCAGGCCCATGGCCTGACCCCGGAGCAGGTGGTGGCCATCGCCAGCCACGATGGCGGCAAGCAGGCGCTGGAGACGGTGCAGCGGCTGTTGCCGGTGCTGTGCCAGGCCCATGGCCTGACCCCGCAGCAGGTGGTGGCCATCGCCAGCAATGGCGGCGGCAGGCCGGCGCTGGAGAGCATTGTTGCCCAGTTATCTCGCCCTGATCCGGCGTTGGCCGCGTTGACCAACGACCACCTCGTCGCCTTGGCCTGCCTCGGCGGACGTCCTGCGCTGGATGCAGTGAAAAAGGGATTGCCGCACGCGCCGGCCTTGATCAAAAGAACCAATCGCCGTATTCCCGAACGCACATCCCATCGCGTTGCCGACCACGCGCAAGTGGTTCGCGTGCTGGGTTTTTTCCAGTGCCACTCCCACCCAGCGCAAGCATTTGATGACGCCATGACGCAGTTCGGGATGAGCAGGCACGGGTTGTTACAGCTCTTTCGCAGAGTGGGCGTCACCGAACTCGAAGCCCGCAGTGGAACGCTCCCCCCAGCCTCGCAGCGTTGGGACCGTATCCTCCAGGCATCAGGGATGAAAAGGGCCAAACCGTCCCCTACTTCAACTCAAACGCCGGATCAGGCGTCTTTGCATGCATTCGCCGATTCGCTGGAGCGTGACCTTGATGCGCCTAGCCCAATGCACGAGGGAGATCAGACGCGGGCAAGCAGCCGTAAACGGTCCCGATCGGATCGTGCTGTCACCGGTCCCTCCGCACAGCAATCGTTCGAGGTGCGCGTTCCCGAACAGCGCGATGCGCTGCATTTGCCCCTCAGTTGGAGGGTAAAACGCCCGCGTACCAGTATCGGGGGCGGCCTCCCGGATCCTGGTACGCCCACGGCTGCCGACCTGGCAGCGTCCAGCACCGTGATGCGGGAACAAGATGAGGACCCCTTCGCAGGGGCAGCGGATGATTTCCCGGCATTCAACGAAGAGGAGCTCGCATGGTTGATGGAGCTATTGCCTCAGTGAAGCTT

**Amino acid sequence c-Myc-AvrBs3-N4(TalC)**

MGLINGEQKLISEEDLNGEQKLISEEDLNGLDGEQKLISEEDLNGEQKLISEEDLNGSHMDPIRSRTPSPARELLPGPQPDGVQPTADRGVSPPAGGPLDGLPARRTMSRTRLPSPPAPSPAFSAGSFSDLLRQFDPSLFNTSLFDSLPPFGAHHTEAATGEWDEVQSGLRAADAPPPTMRVAVTAARPPRAKPAPRRRAAQPSDASPAAQVDLRTLGYSQQQQEKIKPKVRSTVAQHHEALVGHGFTHAHIVALSQHPAALGTVAVKYQDMIAALPEA

-1 THEAIVGVGKQCSGARALEALLTVAGELRGPPLQ

00 LDTGQLVKIAKRGGVTAVEAVHASRNALTGAPLN

01 LTPEQVVAIASHDGGKQALETVQRLLPVLCQAHG

02 LTPQQVVAIASNGGGKQALETVQRLLPVLCQAHG

03 LTPQQVVAIASNSGGKQALETVQRLLPVLCQAHG

04 LTPEQVVAIASNGGGKQALETVQRLLPVLCQAHG

05 LTPEQVVAIASNIGGKQALETVQALLPVLCQAHG

06 LTPEQVVAIASNIGGKQALETVQALLPVLCQAHG

07 LTPEQVVAIASNIGGKQALETVQALLPVLCQAHG

08 LTPEQVVAIASHDGGKQALETVQRLLPVLCQAHG

09 LTPEQVVAIASHDGGKQALETVQRLLPVLCQAHG

10 LTPQQVVAIASNGGGKQALETVQRLLPVLCQAHG

11 LTPEQVVAIASNSGGKQALETVQALLPVLCQAHG

12 LTPEQVVAIASNSGGKQALETVQRLLPVLCQAHG

13 LTPEQVVAIASHDGGKQALETVQRLLPVLCQAHG

14 LTPEQVVAIASHDGGKQALETVQRLLPVLCQAHG

15 LTPEQVVAIASHDGGKQALETVQRLLPVLCQAHG

16 LTPQQVVAIASNGGGRPALETVQRLLPVLCQAHG

17 LTPEQVVAIASHDGGKQALETVQRLLPVLCQAHG

18 LTPQQVVAIASNGGGRPALESIVA

QLSRPDPALAALTNDHLVALACLGGRPALDAVKKGLPHAPALIKRTNRRIPERTSHRVADHAQVVRVLGFFQCHSHPAQAFDDAMTQFGMSRHGLLQLFRRVGVTELEARSGTLPPASQRWDRILQASGMKRAKPSPTSTQTPDQASLHAFADSLERDLDAPSPMHEGDQTRASSRKRSRSDRAVTGPSAQQSFEVRVPEQRDALHLPLSWRVKRPRTSIGGGLPDPGTPTAADLAASSTVMREQDEDPFAGAADDFPAFNEEELAWLMELLPQ*

**DNA sequence *c-myc-avrBs3-N5(talC)***

**ATG**GGGTTAATTAACGGTGAACAAAAGCTAATCTCCGAGGAAGACTTGAACGGTGAACAAAAATTAATCTCAGAAGAAGACTTGAACGGACTCGACGGTGAACAAAAGTTGATTTCTGAAGAAGATTTGAACGGTGAACAAAAGCTAATCTCCGAGGAAGACTTGAACGGTAGCCATATGGATCCCATTCGTTCGCGCACACCAAGTCCTGCCCGCGAGCTTCTGCCCGGACCCCAACCCGATGGGGTTCAGCCGACTGCAGATCGTGGGGTGTCTCCGCCTGCCGGCGGCCCCCTGGATGGCTTGCCCGCTCGGCGGACGATGTCCCGGACCCGGCTGCCATCTCCCCCTGCCCCCTCACCTGCGTTCTCGGCGGGCAGCTTCAGTGACCTGTTACGTCAGTTCGATCCGTCACTTTTTAATACATCGCTTTTTGATTCATTGCCTCCCTTCGGCGCTCACCATACAGAGGCTGCCACAGGCGAGTGGGATGAGGTGCAATCGGGTCTGCGGGCAGCCGACGCCCCCCCACCCACCATGCGCGTGGCTGTCACTGCCGCGCGGCCGCCGCGCGCCAAGCCGGCGCCGCGACGACGTGCTGCGCAACCCTCCGACGCTTCGCCGGCCGCGCAGGTGGATCTACGCACGCTCGGCTACAGCCAGCAGCAACAGGAGAAGATCAAACCGAAGGTTCGTTCGACAGTGGCGCAGCACCACGAGGCACTGGTCGGCCATGGGTTTACACACGCGCACATCGTTGCGCTCAGCCAACACCCGGCAGCGTTAGGGACCGTCGCTGTCAAGTATCAGGACATGATCGCAGCGTTGCCAGAGGCGACACACGAAGCGATCGTTGGCGTCGGCAAACAGTGGTCCGGCGCACGCGCTCTGGAGGCCTTGCTCACGGTGGCGGGAGAGTTGAGAGGTCCACCGTTACAGTTGGACACAGGCCAACTTGTCAAGATTGCAAAACGTGGCGGCGTGACCGCAGTGGAGGCAGTGCATGCATCGCGCAATGCACTGACGGGTGCCCCCCTGAACCTGACCCCGGAGCAGGTGGTGGCCATCGCCAGCCACGATGGCGGCAAGCAGGCGCTGGAGACGGTGCAGCGGCTGTTGCCGGTGCTGTGCCAGGCCCATGGCCTGACCCCGCAGCAGGTGGTGGCCATCGCCAGCAATGGCGGTGGCAAGCAGGCGCTGGAGACGGTGCAGCGGCTGTTGCCGGTGCTGTGCCAGGCCCATGGCCTGACCCCGCAGCAGGTGGTGGCCATCGCCAGCAATAGCGGTGGCAAGCAGGCGCTGGAGACGGTGCAGCGGCTGTTGCCGGTGCTGTGCCAGGCCCATGGCCTGACCCCGGAGCAGGTGGTGGCCATCGCCAGCAATGGCGGTGGCAAGCAGGCGCTGGAGACGGTGCAGCGGCTGTTGCCGGTGCTGTGCCAGGCCCATGGCCTGACCCCGGAGCAGGTGGTGGCCATCGCCAGCAATATTGGTGGCAAGCAGGCGCTGGAGACGGTGCAGGCGCTGTTGCCGGTGCTGTGCCAGGCCCATGGCCTGACCCCGGAGCAGGTGGTGGCCATCGCCAGCAATATTGGTGGCAAGCAGGCGCTGGAGACGGTGCAGGCGCTGTTGCCGGTGCTGTGCCAGGCCCATGGCCTGACCCCGGAGCAGGTGGTGGCCATCGCCAGCAATATTGGTGGCAAGCAGGCGCTGGAGACGGTGCAGGCGCTGTTGCCGGTGCTGTGCCAGGCCCATGGCCTGACCCCGGAGCAGGTGGTGGCCATCGCCAGCCACGATGGCGGCAAGCAGGCGCTGGAGACGGTGCAGCGGCTGTTGCCGGTGCTGTGCCAGGCCCATGGCCTGACCCCGGAGCAGGTGGTGGCCATCGCCAGCCACGATGGCGGCAAGCAGGCGCTGGAGACGGTGCAGCGGCTGTTGCCGGTGCTGTGCCAGGCCCATGGCCTGACCCCGCAGCAGGTGGTGGCCATCGCCAGCAATGGCGGTGGCAAGCAGGCGCTGGAGACGGTGCAGCGGCTGTTGCCGGTGCTGTGCCAGGCCCATGGCCTGACCCCGGAGCAGGTGGTGGCCATCGCCAGCAATAGCGGTGGCAAGCAGGCGCTGGAGACGGTGCAGGCGCTGTTGCCGGTGCTGTGCCAGGCCCATGGCCTGACCCCGGAGCAGGTGGTGGCCATCGCCAGCAATAGCGGTGGCAAGCAGGCGCTGGAGACGGTGCAGCGGCTGTTGCCGGTGCTGTGCCAGGCCCATGGCCTGACCCCGGAGCAGGTGGTGGCCATCGCCAGCCACGATGGCGGCAAGCAGGCGCTGGAGACGGTGCAGCGGCTGTTGCCGGTGCTGTGCCAGGCCCATGGCCTGACCCCGGAGCAGGTGGTGGCCATCGCCAGCCACGATGGCGGCAAGCAGGCGCTGGAGACGGTGCAGCGGCTGTTGCCGGTGCTGTGCCAGGCCCATGGCCTGACCCCGGAGCAGGTGGTGGCCATCGCCAGCCACGATGGCGGCAAGCAGGCGCTGGAGACGGTGCAGCGGCTGTTGCCGGTGCTGTGCCAGGCCCATGGCCTGACCCCGCAGCAGGTGGTGGCCATCGCCAGCAATGGCGGCGGCAGGCCGGCGCTGGAGACGGTGCAGCGGCTGTTGCCGGTGCTGTGCCAGGCCCATGGCCTGACCCCGGAGCAGGTGGTGGCCATCGCCAGCCACGATGGCGGCAAGCAGGCGCTGGAGACGGTGCAGCGGCTGTTGCCGGTGCTGTGCCAGGCCCATGGCCTGACCCCGCAGCAGGTGGTGGCCATCGCCAGCAATGGCGGCGGCAGGCCGGCGCTGGAGAGCATTGTTGCCCAGTTATCTCGCCCTGATCCGGCGTTGGCCGCGTTGACCAACGACCACCTCGTCGCCTTGGCCTGCCTCGGCGGACGTCCTGCGCTGGATGCAGTGAAAAAGGGATTGCCGCACGCGCCGGCCTTGATCAAAAGAACCAATCGCCGTATTCCCGAACGCACATCCCATCGCGTTGCCGACCACGCGCAAGTGGTTCGCGTGCTGGGTTTTTTCCAGTGCCACTCCCACCCAGCGCAAGCATTTGATGACGCCATGACGCAGTTCGGGATGAGCAGGCACGGGTTGTTACAGCTCTTTCGCAGAGTGGGCGTCACCGAACTCGAAGCCCGCAGTGGAACGCTCCCCCCAGCCTCGCAGCGTTGGGACCGTATCCTCCAGGCATCAGGGATGAAAAGGGCCAAACCGTCCCCTACTTCAACTCAAACGCCGGATCAGGCGTCTTTGCATGCATTCGCCGATTCGCTGGAGCGTGACCTTGATGCGCCTAGCCCAATGCACGAGGGAGATCAGACGCGGGCAAGCAGCCGTAAACGGTCCCGATCGGATCGTGCTGTCACCGGTCCCTCCGCACAGCAATCGTTCGAGGTGCGCGTTCCCGAACAGCGCGATGCGCTGCATTTGCCCCTCAGTTGGAGGGTAAAACGCCCGCGTACCAGTATCGGGGGCGGCCTCCCGGATCCTGGTACGCCCACGGCTGCCGACCTGGCAGCGTCCAGCACCGTGATGCGGGAACAAGATGAGGACCCCTTCGCAGGGGCAGCGGATGATTTCCCGGCATTCAACGAAGAGGAGCTCGCATGGTTGATGGAGCTATTGCCTCAGTGAAGCTT

**Amino acid sequence c-Myc-AvrBs3-N5(TalC)**

MGLINGEQKLISEEDLNGEQKLISEEDLNGLDGEQKLISEEDLNGEQKLISEEDLNGSHMDPIRSRTPSPARELLPGPQPDGVQPTADRGVSPPAGGPLDGLPARRTMSRTRLPSPPAPSPAFSAGSFSDLLRQFDPSLFNTSLFDSLPPFGAHHTEAATGEWDEVQSGLRAADAPPPTMRVAVTAARPPRAKPAPRRRAAQPSDASPAAQVDLRTLGYSQQQQEKIKPKVRSTVAQHHEALVGHGFTHAHIVALSQHPAALGTVAVKYQDMIAALPEA

-1 THEAIVGVGKQWSGARALEALLTVAGELRGPPLQ

00 LDTGQLVKIAKRGGVTAVEAVHASRNALTGAPLN

01 LTPEQVVAIASHDGGKQALETVQRLLPVLCQAHG

02 LTPQQVVAIASNGGGKQALETVQRLLPVLCQAHG

03 LTPQQVVAIASNSGGKQALETVQRLLPVLCQAHG

04 LTPEQVVAIASNGGGKQALETVQRLLPVLCQAHG

05 LTPEQVVAIASNIGGKQALETVQALLPVLCQAHG

06 LTPEQVVAIASNIGGKQALETVQALLPVLCQAHG

07 LTPEQVVAIASNIGGKQALETVQALLPVLCQAHG

08 LTPEQVVAIASHDGGKQALETVQRLLPVLCQAHG

09 LTPEQVVAIASHDGGKQALETVQRLLPVLCQAHG

10 LTPQQVVAIASNGGGKQALETVQRLLPVLCQAHG

11 LTPEQVVAIASNSGGKQALETVQALLPVLCQAHG

12 LTPEQVVAIASNSGGKQALETVQRLLPVLCQAHG

13 LTPEQVVAIASHDGGKQALETVQRLLPVLCQAHG

14 LTPEQVVAIASHDGGKQALETVQRLLPVLCQAHG

15 LTPEQVVAIASHDGGKQALETVQRLLPVLCQAHG

16 LTPQQVVAIASNGGGRPALETVQRLLPVLCQAHG

17 LTPEQVVAIASHDGGKQALETVQRLLPVLCQAHG

18 LTPQQVVAIASNGGGRPALESIVA

QLSRPDPALAALTNDHLVALACLGGRPALDAVKKGLPHAPALIKRTNRRIPERTSHRVADHAQVVRVLGFFQCHSHPAQAFDDAMTQFGMSRHGLLQLFRRVGVTELEARSGTLPPASQRWDRILQASGMKRAKPSPTSTQTPDQASLHAFADSLERDLDAPSPMHEGDQTRASSRKRSRSDRAVTGPSAQQSFEVRVPEQRDALHLPLSWRVKRPRTSIGGGLPDPGTPTAADLAASSTVMREQDEDPFAGAADDFPAFNEEELAWLMELLPQ*

**DNA sequence designer-*avrBs3*(RVD1=XX) (17.5 repeats)**

**ATG**GGGTTAATTAACGGTGAACAAAAGCTAATCTCCGAGGAAGACTTGAACGGTGAACAAAAATTAATCTCAGAAGAAGACTTGAACGGACTCGACGGTGAACAAAAGTTGATTTCTGAAGAAGATTTGAACGGTGAACAAAAGCTAATCTCCGAGGAAGACTTGAACGGTAGCCATATGGATCCCATTCGTTCGCGCACACCAAGTCCTGCCCGCGAGCTTCTGCCCGGACCCCAACCCGATGGGGTTCAGCCGACTGCAGATCGTGGGGTGTCTCCGCCTGCCGGCGGCCCCCTGGATGGCTTGCCCGCTCGGCGGACGATGTCCCGGACCCGGCTGCCATCTCCCCCTGCCCCCTCACCTGCGTTCTCGGCGGGCAGCTTCAGTGACCTGTTACGTCAGTTCGATCCGTCACTTTTTAATACATCGCTTTTTGATTCATTGCCTCCCTTCGGCGCTCACCATACAGAGGCTGCCACAGGCGAGTGGGATGAGGTGCAATCGGGTCTGCGGGCAGCCGACGCCCCCCCACCCACCATGCGCGTGGCTGTCACTGCCGCGCGGCCGCCGCGCGCCAAGCCGGCGCCGCGACGACGTGCTGCGCAACCCTCCGACGCTTCGCCGGCCGCGCAGGTGGATCTACGCACGCTCGGCTACAGCCAGCAGCAACAGGAGAAGATCAAACCGAAGGTTCGTTCGACAGTGGCGCAGCACCACGAGGCACTGGTCGGCCATGGGTTTACACACGCGCACATCGTTGCGCTCAGCCAACACCCGGCAGCGTTAGGGACCGTCGCTGTCAAGTATCAGGACATGATCGCAGCGTTGCCAGAGGCGACACACGAAGCGATCGTTGGCGTCGGCAAACAGTGGTCCGGCGCACGCGCTCTGGAGGCCTTGCTCACGGTGGCGGGAGAGTTGAGAGGTCCACCGTTACAGTTGGACACAGGCCAACTTCTCAAGATTGCAAAACGTGGCGGCGTGACCGCAGTGGAGGCAGTGCATGCATGGCGCAATGCACTGACGGGTGCCCCCCTGAACCTTACCCCGGAGCAGGTGGTGGCCATCGCCAGCXXXXXXGGTGGCAAGCAGGCGCTGGAGACGGTGCAGCGGCTGTTGCCGGTGCTGTGCCAGGCCCATGGCCTGACCCCGGAGCAGGTGGTGGCCATCGCCAGCAATGGCGGCGGCAAGCAGGCGCTGGAGACGGTGCAGCGGCTGTTGCCGGTGCTGTGCCAGGCCCATGGCCTGACACCGGAGCAGGTGGTGGCCATCGCCAGCAATAGCGGTGGCAAGCAGGCGCTGGAGACGGTGCAGCGGCTGTTGCCGGTGCTGTGCCAGGCCCATGGCCTCACCCCGGAGCAGGTGGTGGCCATCGCCAGCAATGGCGGCGGCAAGCAGGCGCTGGAGACGGTGCAGCGGCTGTTGCCGGTGCTGTGCCAGGCCCATGGCCTGACTCCGGAGCAGGTGGTGGCCATCGCCAGCAATATTGGCGGCAAGCAGGCGCTGGAGACGGTGCAGCGGCTGTTGCCGGTGCTGTGCCAGGCCCATGGCCTGACCCCGGAGCAGGTGGTGGCCATCGCCAGCAATATTGGCGGCAAGCAGGCGCTGGAGACGGTGCAGCGGCTGTTGCCGGTGCTGTGCCAGGCGCATGGCCTTACCCCGGAGCAGGTGGTGGCCATCGCCAGCAATATTGGTGGCAAGCAGGCGCTGGAGACGGTGCAGCGGCTGTTGCCGGTGCTGTGCCAGGCCCATGGCCTGACCCCGGAGCAGGTGGTGGCCATCGCCAGCCACGATGGCGGCAAGCAGGCGCTGGAGACGGTGCAGCGGCTGTTGCCGGTGCTGTGCCAGGCCCATGGCCTGACACCGGAGCAGGTGGTGGCCATCGCCAGCCACGATGGTGGCAAGCAGGCGCTGGAGACGGTGCAGCGGCTGTTGCCGGTGCTGTGCCAGGCCCATGGCCTCACCCCGGAGCAGGTGGTGGCCATCGCCAGCAATGGCGGCGGCAAGCAGGCGCTGGAGACGGTGCAGCGGCTGTTGCCGGTGCTGTGCCAGGCCCATGGCCTGACTCCGGAGCAGGTGGTGGCCATCGCCAGCAATAGCGGCGGCAAGCAGGCGCTGGAGACGGTGCAGCGGCTGTTGCCGGTGCTGTGCCAGGCCCATGGCCTGACCCCGGAGCAGGTGGTGGCCATCGCCAGCAATAGCGGCGGCAAGCAGGCGCTGGAGACGGTGCAGCGGCTGTTGCCGGTGCTGTGCCAGGCGCATGGCCTTACCCCGGAGCAGGTGGTGGCCATCGCCAGCCACGATGGTGGCAAGCAGGCGCTGGAGACGGTGCAGCGGCTGTTGCCGGTGCTGTGCCAGGCCCATGGCCTGACCCCGGAGCAGGTGGTGGCCATCGCCAGCCACGATGGCGGCAAGCAGGCGCTGGAGACGGTGCAGCGGCTGTTGCCGGTGCTGTGCCAGGCCCATGGCCTGACACCGGAGCAGGTGGTGGCCATCGCCAGCCACGATGGTGGCAAGCAGGCGCTGGAGACGGTGCAGCGGCTGTTGCCGGTGCTGTGCCAGGCCCATGGCCTCACCCCGGAGCAGGTGGTGGCCATCGCCAGCAATGGCGGCGGCAAGCAGGCGCTGGAGACGGTGCAGCGGCTGTTGCCGGTGCTGTGCCAGGCCCATGGCCTGACTCCGGAGCAGGTGGTGGCCATCGCCAGCCACGATGGCGGCAAGCAGGCGCTGGAGACGGTGCAGCGGCTGTTGCCGGTGCTGTGCCAGGCCCATGGCCTGACCCCGGAGCAGGTGGTGGCCATCGCCAGCAATGGCGGCGGCAAGCAGGCGCTGGAGAGCATTGTTGCCCAGTTATCTCGCCCTGATCCGGCGTTGGCCGCGTTGACCAACGACCACCTCGTCGCCTTGGCCTGCCTCGGCGGACGTCCTGCGCTGGATGCAGTGAAAAAGGGATTGCCGCACGCGCCGGCCTTGATCAAAAGAACCAATCGCCGTATTCCCGAACGCACATCCCATCGCGTTGCCGACCACGCGCAAGTGGTTCGCGTGCTGGGTTTTTTCCAGTGCCACTCCCACCCAGCGCAAGCATTTGATGACGCCATGACGCAGTTCGGGATGAGCAGGCACGGGTTGTTACAGCTCTTTCGCAGAGTGGGCGTCACCGAACTCGAAGCCCGCAGTGGAACGCTCCCCCCAGCCTCGCAGCGTTGGGACCGTATCCTCCAGGCATCAGGGATGAAAAGGGCCAAACCGTCCCCTACTTCAACTCAAACGCCGGATCAGGCGTCTTTGCATGCATTCGCCGATTCGCTGGAGCGTGACCTTGATGCGCCTAGCCCAATGCACGAGGGAGATCAGACGCGGGCAAGCAGCCGTAAACGGTCCCGATCGGATCGTGCTGTCACCGGTCCCTCCGCACAGCAATCGTTCGAGGTGCGCGTTCCCGAACAGCGCGATGCGCTGCATTTGCCCCTCAGTTGGAGGGTAAAACGCCCGCGTACCAGTATCGGGGGCGGCCTCCCGGATCCTGGTACGCCCACGGCTGCCGACCTGGCAGCGTCCAGCACCGTGATGCGGGAACAAGATGAGGACCCCTTCGCAGGGGCAGCGGATGATTTCCCGGCATTCAACGAAGAGGAGCTCGCATGGTTGATGGAGCTATTGCCTCAGTGAAGCTT

**RVD1 = codon usage**

NK = AAT AAG

HD = CAC GAT

NS = AAT AGC

NH = AAT CAT

NI = AAT ATT

NN = AAT AAT

NG = AAT GGC

**Amino acid sequence dAvrBs3(RVD1=XX) (17.5 repeats)**

MGLINGEQKLISEEDLNGEQKLISEEDLNGLDGEQKLISEEDLNGEQKLISEEDLNGSHMDPIRSRTPSPARELLPGPQPDGVQPTADRGVSPPAGGPLDGLPARRTMSRTRLPSPPAPSPAFSAGSFSDLLRQFDPSLFNTSLFDSLPPFGAHHTEAATGEWDEVQSGLRAADAPPPTMRVAVTAARPPRAKPAPRRRAAQPSDASPAAQVDLRTLGYSQQQQEKIKPKVRSTVAQHHEALVGHGFTHAHIVALSQHPAALGTVAVKYQDMIAALPEA

-1 THEAIVGVGKQWSGARALEALLTVAGELRGPPLQ

00 LDTGQLLKIAKRGGVTAVEAVHAWRNALTGAPLN

01 LTPEQVVAIASXXGGKQALETVQRLLPVLCQAHG

02 LTPEQVVAIASNGGGKQALETVQRLLPVLCQAHG

03 LTPEQVVAIASNSGGKQALETVQRLLPVLCQAHG

04 LTPEQVVAIASNGGGKQALETVQRLLPVLCQAHG

05 LTPEQVVAIASNIGGKQALETVQRLLPVLCQAHG

06 LTPEQVVAIASNIGGKQALETVQRLLPVLCQAHG

07 LTPEQVVAIASNIGGKQALETVQRLLPVLCQAHG

08 LTPEQVVAIASHDGGKQALETVQRLLPVLCQAHG

09 LTPEQVVAIASHDGGKQALETVQRLLPVLCQAHG

10 LTPEQVVAIASNGGGKQALETVQRLLPVLCQAHG

11 LTPEQVVAIASNSGGKQALETVQRLLPVLCQAHG

12 LTPEQVVAIASNSGGKQALETVQRLLPVLCQAHG

13 LTPEQVVAIASHDGGKQALETVQRLLPVLCQAHG

14 LTPEQVVAIASHDGGKQALETVQRLLPVLCQAHG

15 LTPEQVVAIASHDGGKQALETVQRLLPVLCQAHG

16 LTPEQVVAIASNGGGKQALETVQRLLPVLCQAHG

17 LTPEQVVAIASHDGGKQALETVQRLLPVLCQAHG

18 LTPEQVVAIASNGGGKQALESIVA

QLSRPDPALAALTNDHLVALACLGGRPALDAVKKGLPHAPALIKRTNRRIPERTSHRVADHAQVVRVLGFFQCHSHPAQAFDDAMTQFGMSRHGLLQLFRRVGVTELEARSGTLPPASQRWDRILQASGMKRAKPSPTSTQTPDQASLHAFADSLERDLDAPSPMHEGDQTRASSRKRSRSDRAVTGPSAQQSFEVRVPEQRDALHLPLSWRVKRPRTSIGGGLPDPGTPTAADLAASSTVMREQDEDPFAGAADDFPAFNEEELAWLMELLPQ*

**DNA sequence designer-*avrBs3*(RVD1=XX) (13.5 repeats)**

**ATG**GGGTTAATTAACGGTGAACAAAAGCTAATCTCCGAGGAAGACTTGAACGGTGAACAAAAATTAATCTCAGAAGAAGACTTGAACGGACTCGACGGTGAACAAAAGTTGATTTCTGAAGAAGATTTGAACGGTGAACAAAAGCTAATCTCCGAGGAAGACTTGAACGGTAGCCATATGGATCCCATTCGTTCGCGCACACCAAGTCCTGCCCGCGAGCTTCTGCCCGGACCCCAACCCGATGGGGTTCAGCCGACTGCAGATCGTGGGGTGTCTCCGCCTGCCGGCGGCCCCCTGGATGGCTTGCCCGCTCGGCGGACGATGTCCCGGACCCGGCTGCCATCTCCCCCTGCCCCCTCACCTGCGTTCTCGGCGGGCAGCTTCAGTGACCTGTTACGTCAGTTCGATCCGTCACTTTTTAATACATCGCTTTTTGATTCATTGCCTCCCTTCGGCGCTCACCATACAGAGGCTGCCACAGGCGAGTGGGATGAGGTGCAATCGGGTCTGCGGGCAGCCGACGCCCCCCCACCCACCATGCGCGTGGCTGTCACTGCCGCGCGGCCGCCGCGCGCCAAGCCGGCGCCGCGACGACGTGCTGCGCAACCCTCCGACGCTTCGCCGGCCGCGCAGGTGGATCTACGCACGCTCGGCTACAGCCAGCAGCAACAGGAGAAGATCAAACCGAAGGTTCGTTCGACAGTGGCGCAGCACCACGAGGCACTGGTCGGCCATGGGTTTACACACGCGCACATCGTTGCGCTCAGCCAACACCCGGCAGCGTTAGGGACCGTCGCTGTCAAGTATCAGGACATGATCGCAGCGTTGCCAGAGGCGACACACGAAGCGATCGTTGGCGTCGGCAAACAGTGGTCCGGCGCACGCGCTCTGGAGGCCTTGCTCACGGTGGCGGGAGAGTTGAGAGGTCCACCGTTACAGTTGGACACAGGCCAACTTCTCAAGATTGCAAAACGTGGCGGCGTGACCGCAGTGGAGGCAGTGCATGCATGGCGCAATGCACTGACGGGTGCCCCCCTGAACCTTACCCCGGAGCAGGTGGTGGCCATCGCCAGCXXXXXXGGTGGCAAGCAGGCGCTGGAGACGGTGCAGCGGCTGTTGCCGGTGCTGTGCCAGGCCCATGGCCTGACCCCGGAGCAGGTGGTGGCCATCGCCAGCAATGGCGGCGGCAAGCAGGCGCTGGAGACGGTGCAGCGGCTGTTGCCGGTGCTGTGCCAGGCCCATGGCCTGACACCGGAGCAGGTGGTGGCCATCGCCAGCAATAGCGGTGGCAAGCAGGCGCTGGAGACGGTGCAGCGGCTGTTGCCGGTGCTGTGCCAGGCCCATGGCCTCACCCCGGAGCAGGTGGTGGCCATCGCCAGCAATGGCGGCGGCAAGCAGGCGCTGGAGACGGTGCAGCGGCTGTTGCCGGTGCTGTGCCAGGCCCATGGCCTGACTCCGGAGCAGGTGGTGGCCATCGCCAGCAATATTGGCGGCAAGCAGGCGCTGGAGACGGTGCAGCGGCTGTTGCCGGTGCTGTGCCAGGCCCATGGCCTGACCCCGGAGCAGGTGGTGGCCATCGCCAGCAATATTGGCGGCAAGCAGGCGCTGGAGACGGTGCAGCGGCTGTTGCCGGTGCTGTGCCAGGCGCATGGCCTTACCCCGGAGCAGGTGGTGGCCATCGCCAGCAATATTGGTGGCAAGCAGGCGCTGGAGACGGTGCAGCGGCTGTTGCCGGTGCTGTGCCAGGCCCATGGCCTGACCCCGGAGCAGGTGGTGGCCATCGCCAGCCACGATGGCGGCAAGCAGGCGCTGGAGACGGTGCAGCGGCTGTTGCCGGTGCTGTGCCAGGCCCATGGCCTGACACCGGAGCAGGTGGTGGCCATCGCCAGCCACGATGGTGGCAAGCAGGCGCTGGAGACGGTGCAGCGGCTGTTGCCGGTGCTGTGCCAGGCCCATGGCCTCACCCCGGAGCAGGTGGTGGCCATCGCCAGCAATGGCGGCGGCAAGCAGGCGCTGGAGACGGTGCAGCGGCTGTTGCCGGTGCTGTGCCAGGCCCATGGCCTGACTCCGGAGCAGGTGGTGGCCATCGCCAGCAATAGCGGCGGCAAGCAGGCGCTGGAGACGGTGCAGCGGCTGTTGCCGGTGCTGTGCCAGGCCCATGGCCTGACCCCGGAGCAGGTGGTGGCCATCGCCAGCAATAGCGGCGGCAAGCAGGCGCTGGAGACGGTGCAGCGGCTGTTGCCGGTGCTGTGCCAGGCGCATGGCCTTACCCCGGAGCAGGTGGTGGCCATCGCCAGCCACGATGGTGGCAAGCAGGCGCTGGAGACGGTGCAGCGGCTGTTGCCGGTGCTGTGCCAGGCCCATGGCCTGACCCCGGAGCAGGTGGTGGCCATCGCCAGCCACGATGGCGGCAAGCAGGCGCTGGAGAGCATTGTTGCCCAGTTATCTCGCCCTGATCCGGCGTTGGCCGCGTTGACCAACGACCACCTCGTCGCCTTGGCCTGCCTCGGCGGACGTCCTGCGCTGGATGCAGTGAAAAAGGGATTGCCGCACGCGCCGGCCTTGATCAAAAGAACCAATCGCCGTATTCCCGAACGCACATCCCATCGCGTTGCCGACCACGCGCAAGTGGTTCGCGTGCTGGGTTTTTTCCAGTGCCACTCCCACCCAGCGCAAGCATTTGATGACGCCATGACGCAGTTCGGGATGAGCAGGCACGGGTTGTTACAGCTCTTTCGCAGAGTGGGCGTCACCGAACTCGAAGCCCGCAGTGGAACGCTCCCCCCAGCCTCGCAGCGTTGGGACCGTATCCTCCAGGCATCAGGGATGAAAAGGGCCAAACCGTCCCCTACTTCAACTCAAACGCCGGATCAGGCGTCTTTGCATGCATTCGCCGATTCGCTGGAGCGTGACCTTGATGCGCCTAGCCCAATGCACGAGGGAGATCAGACGCGGGCAAGCAGCCGTAAACGGTCCCGATCGGATCGTGCTGTCACCGGTCCCTCCGCACAGCAATCGTTCGAGGTGCGCGTTCCCGAACAGCGCGATGCGCTGCATTTGCCCCTCAGTTGGAGGGTAAAACGCCCGCGTACCAGTATCGGGGGCGGCCTCCCGGATCCTGGTACGCCCACGGCTGCCGACCTGGCAGCGTCCAGCACCGTGATGCGGGAACAAGATGAGGACCCCTTCGCAGGGGCAGCGGATGATTTCCCGGCATTCAACGAAGAGGAGCTCGCATGGTTGATGGAGCTATTGCCTCAGTGAAGCTT

**Amino acid sequence dAvrBs3(RVD1=XX) (13.5 repeats)**

MGLINGEQKLISEEDLNGEQKLISEEDLNGLDGEQKLISEEDLNGEQKLISEEDLNGSHMDPIRSRTPSPARELLPGPQPDGVQPTADRGVSPPAGGPLDGLPARRTMSRTRLPSPPAPSPAFSAGSFSDLLRQFDPSLFNTSLFDSLPPFGAHHTEAATGEWDEVQSGLRAADAPPPTMRVAVTAARPPRAKPAPRRRAAQPSDASPAAQVDLRTLGYSQQQQEKIKPKVRSTVAQHHEALVGHGFTHAHIVALSQHPAALGTVAVKYQDMIAALPEA

-1 THEAIVGVGKQWSGARALEALLTVAGELRGPPLQ

00 LDTGQLLKIAKRGGVTAVEAVHAWRNALTGAPLN

01 LTPEQVVAIASXXGGKQALETVQRLLPVLCQAHG

02 LTPEQVVAIASNGGGKQALETVQRLLPVLCQAHG

03 LTPEQVVAIASNSGGKQALETVQRLLPVLCQAHG

04 LTPEQVVAIASNGGGKQALETVQRLLPVLCQAHG

05 LTPEQVVAIASNIGGKQALETVQRLLPVLCQAHG

06 LTPEQVVAIASNIGGKQALETVQRLLPVLCQAHG

07 LTPEQVVAIASNIGGKQALETVQRLLPVLCQAHG

08 LTPEQVVAIASHDGGKQALETVQRLLPVLCQAHG

09 LTPEQVVAIASHDGGKQALETVQRLLPVLCQAHG

10 LTPEQVVAIASNGGGKQALETVQRLLPVLCQAHG

11 LTPEQVVAIASNSGGKQALETVQRLLPVLCQAHG

12 LTPEQVVAIASNSGGKQALETVQRLLPVLCQAHG

13 LTPEQVVAIASHDGGKQALETVQRLLPVLCQAHG

14 LTPEQVVAIASHDGGKQALESIVA

QLSRPDPALAALTNDHLVALACLGGRPALDAVKKGLPHAPALIKRTNRRIPERTSHRVADHAQVVRVLGFFQCHSHPAQAFDDAMTQFGMSRHGLLQLFRRVGVTELEARSGTLPPASQRWDRILQASGMKRAKPSPTSTQTPDQASLHAFADSLERDLDAPSPMHEGDQTRASSRKRSRSDRAVTGPSAQQSFEVRVPEQRDALHLPLSWRVKRPRTSIGGGLPDPGTPTAADLAASSTVMREQDEDPFAGAADDFPAFNEEELAWLMELLPQ*

**DNA sequence designer-*avrBs3*(RVD1=XX) (12.5 repeats)**

**ATG**GGGTTAATTAACGGTGAACAAAAGCTAATCTCCGAGGAAGACTTGAACGGTGAACAAAAATTAATCTCAGAAGAAGACTTGAACGGACTCGACGGTGAACAAAAGTTGATTTCTGAAGAAGATTTGAACGGTGAACAAAAGCTAATCTCCGAGGAAGACTTGAACGGTAGCCATATGGATCCCATTCGTTCGCGCACACCAAGTCCTGCCCGCGAGCTTCTGCCCGGACCCCAACCCGATGGGGTTCAGCCGACTGCAGATCGTGGGGTGTCTCCGCCTGCCGGCGGCCCCCTGGATGGCTTGCCCGCTCGGCGGACGATGTCCCGGACCCGGCTGCCATCTCCCCCTGCCCCCTCACCTGCGTTCTCGGCGGGCAGCTTCAGTGACCTGTTACGTCAGTTCGATCCGTCACTTTTTAATACATCGCTTTTTGATTCATTGCCTCCCTTCGGCGCTCACCATACAGAGGCTGCCACAGGCGAGTGGGATGAGGTGCAATCGGGTCTGCGGGCAGCCGACGCCCCCCCACCCACCATGCGCGTGGCTGTCACTGCCGCGCGGCCGCCGCGCGCCAAGCCGGCGCCGCGACGACGTGCTGCGCAACCCTCCGACGCTTCGCCGGCCGCGCAGGTGGATCTACGCACGCTCGGCTACAGCCAGCAGCAACAGGAGAAGATCAAACCGAAGGTTCGTTCGACAGTGGCGCAGCACCACGAGGCACTGGTCGGCCATGGGTTTACACACGCGCACATCGTTGCGCTCAGCCAACACCCGGCAGCGTTAGGGACCGTCGCTGTCAAGTATCAGGACATGATCGCAGCGTTGCCAGAGGCGACACACGAAGCGATCGTTGGCGTCGGCAAACAGTGGTCCGGCGCACGCGCTCTGGAGGCCTTGCTCACGGTGGCGGGAGAGTTGAGAGGTCCACCGTTACAGTTGGACACAGGCCAACTTCTCAAGATTGCAAAACGTGGCGGCGTGACCGCAGTGGAGGCAGTGCATGCATGGCGCAATGCACTGACGGGTGCCCCCCTGAACCTTACCCCGGAGCAGGTGGTGGCCATCGCCAGCXXXXXXGGTGGCAAGCAGGCGCTGGAGACGGTGCAGCGGCTGTTGCCGGTGCTGTGCCAGGCCCATGGCCTGACCCCGGAGCAGGTGGTGGCCATCGCCAGCAATGGCGGCGGCAAGCAGGCGCTGGAGACGGTGCAGCGGCTGTTGCCGGTGCTGTGCCAGGCCCATGGCCTGACACCGGAGCAGGTGGTGGCCATCGCCAGCAATAGCGGTGGCAAGCAGGCGCTGGAGACGGTGCAGCGGCTGTTGCCGGTGCTGTGCCAGGCCCATGGCCTCACCCCGGAGCAGGTGGTGGCCATCGCCAGCAATGGCGGCGGCAAGCAGGCGCTGGAGACGGTGCAGCGGCTGTTGCCGGTGCTGTGCCAGGCCCATGGCCTGACTCCGGAGCAGGTGGTGGCCATCGCCAGCAATATTGGCGGCAAGCAGGCGCTGGAGACGGTGCAGCGGCTGTTGCCGGTGCTGTGCCAGGCCCATGGCCTGACCCCGGAGCAGGTGGTGGCCATCGCCAGCAATATTGGCGGCAAGCAGGCGCTGGAGACGGTGCAGCGGCTGTTGCCGGTGCTGTGCCAGGCGCATGGCCTTACCCCGGAGCAGGTGGTGGCCATCGCCAGCAATATTGGTGGCAAGCAGGCGCTGGAGACGGTGCAGCGGCTGTTGCCGGTGCTGTGCCAGGCCCATGGCCTGACCCCGGAGCAGGTGGTGGCCATCGCCAGCCACGATGGCGGCAAGCAGGCGCTGGAGACGGTGCAGCGGCTGTTGCCGGTGCTGTGCCAGGCCCATGGCCTGACACCGGAGCAGGTGGTGGCCATCGCCAGCCACGATGGTGGCAAGCAGGCGCTGGAGACGGTGCAGCGGCTGTTGCCGGTGCTGTGCCAGGCCCATGGCCTCACCCCGGAGCAGGTGGTGGCCATCGCCAGCAATGGCGGCGGCAAGCAGGCGCTGGAGACGGTGCAGCGGCTGTTGCCGGTGCTGTGCCAGGCCCATGGCCTGACTCCGGAGCAGGTGGTGGCCATCGCCAGCAATAGCGGCGGCAAGCAGGCGCTGGAGACGGTGCAGCGGCTGTTGCCGGTGCTGTGCCAGGCCCATGGCCTGACCCCGGAGCAGGTGGTGGCCATCGCCAGCAATAGCGGCGGCAAGCAGGCGCTGGAGACGGTGCAGCGGCTGTTGCCGGTGCTGTGCCAGGCGCATGGCCTTACCCCGGAGCAGGTGGTGGCCATCGCCAGCCACGATGGTGGCAAGCAGGCGCTGGAGAGCATTGTTGCCCAGTTATCTCGCCCTGATCCGGCGTTGGCCGCGTTGACCAACGACCACCTCGTCGCCTTGGCCTGCCTCGGCGGACGTCCTGCGCTGGATGCAGTGAAAAAGGGATTGCCGCACGCGCCGGCCTTGATCAAAAGAACCAATCGCCGTATTCCCGAACGCACATCCCATCGCGTTGCCGACCACGCGCAAGTGGTTCGCGTGCTGGGTTTTTTCCAGTGCCACTCCCACCCAGCGCAAGCATTTGATGACGCCATGACGCAGTTCGGGATGAGCAGGCACGGGTTGTTACAGCTCTTTCGCAGAGTGGGCGTCACCGAACTCGAAGCCCGCAGTGGAACGCTCCCCCCAGCCTCGCAGCGTTGGGACCGTATCCTCCAGGCATCAGGGATGAAAAGGGCCAAACCGTCCCCTACTTCAACTCAAACGCCGGATCAGGCGTCTTTGCATGCATTCGCCGATTCGCTGGAGCGTGACCTTGATGCGCCTAGCCCAATGCACGAGGGAGATCAGACGCGGGCAAGCAGCCGTAAACGGTCCCGATCGGATCGTGCTGTCACCGGTCCCTCCGCACAGCAATCGTTCGAGGTGCGCGTTCCCGAACAGCGCGATGCGCTGCATTTGCCCCTCAGTTGGAGGGTAAAACGCCCGCGTACCAGTATCGGGGGCGGCCTCCCGGATCCTGGTACGCCCACGGCTGCCGACCTGGCAGCGTCCAGCACCGTGATGCGGGAACAAGATGAGGACCCCTTCGCAGGGGCAGCGGATGATTTCCCGGCATTCAACGAAGAGGAGCTCGCATGGTTGATGGAGCTATTGCCTCAGTGAAGCTT

**Amino acid sequence dAvrBs3(RVD1=XX) (12.5 repeats)**

MGLINGEQKLISEEDLNGEQKLISEEDLNGLDGEQKLISEEDLNGEQKLISEEDLNGSHMDPIRSRTPSPARELLPGPQPDGVQPTADRGVSPPAGGPLDGLPARRTMSRTRLPSPPAPSPAFSAGSFSDLLRQFDPSLFNTSLFDSLPPFGAHHTEAATGEWDEVQSGLRAADAPPPTMRVAVTAARPPRAKPAPRRRAAQPSDASPAAQVDLRTLGYSQQQQEKIKPKVRSTVAQHHEALVGHGFTHAHIVALSQHPAALGTVAVKYQDMIAALPEA

-1 THEAIVGVGKQWSGARALEALLTVAGELRGPPLQ

00 LDTGQLLKIAKRGGVTAVEAVHAWRNALTGAPLN

01 LTPEQVVAIASXXGGKQALETVQRLLPVLCQAHG

02 LTPEQVVAIASNGGGKQALETVQRLLPVLCQAHG

03 LTPEQVVAIASNSGGKQALETVQRLLPVLCQAHG

04 LTPEQVVAIASNGGGKQALETVQRLLPVLCQAHG

05 LTPEQVVAIASNIGGKQALETVQRLLPVLCQAHG

06 LTPEQVVAIASNIGGKQALETVQRLLPVLCQAHG

07 LTPEQVVAIASNIGGKQALETVQRLLPVLCQAHG

08 LTPEQVVAIASHDGGKQALETVQRLLPVLCQAHG

09 LTPEQVVAIASHDGGKQALETVQRLLPVLCQAHG

10 LTPEQVVAIASNGGGKQALETVQRLLPVLCQAHG

11 LTPEQVVAIASNSGGKQALETVQRLLPVLCQAHG

12 LTPEQVVAIASNSGGKQALETVQRLLPVLCQAHG

13 LTPEQVVAIASHDGGKQALESIVA

QLSRPDPALAALTNDHLVALACLGGRPALDAVKKGLPHAPALIKRTNRRIPERTSHRVADHAQVVRVLGFFQCHSHPAQAFDDAMTQFGMSRHGLLQLFRRVGVTELEARSGTLPPASQRWDRILQASGMKRAKPSPTSTQTPDQASLHAFADSLERDLDAPSPMHEGDQTRASSRKRSRSDRAVTGPSAQQSFEVRVPEQRDALHLPLSWRVKRPRTSIGGGLPDPGTPTAADLAASSTVMREQDEDPFAGAADDFPAFNEEELAWLMELLPQ*

**DNA sequence designer-*avrBs3*(RVD1=XX) (11.5 repeats)**

**ATG**GGGTTAATTAACGGTGAACAAAAGCTAATCTCCGAGGAAGACTTGAACGGTGAACAAAAATTAATCTCAGAAGAAGACTTGAACGGACTCGACGGTGAACAAAAGTTGATTTCTGAAGAAGATTTGAACGGTGAACAAAAGCTAATCTCCGAGGAAGACTTGAACGGTAGCCATATGGATCCCATTCGTTCGCGCACACCAAGTCCTGCCCGCGAGCTTCTGCCCGGACCCCAACCCGATGGGGTTCAGCCGACTGCAGATCGTGGGGTGTCTCCGCCTGCCGGCGGCCCCCTGGATGGCTTGCCCGCTCGGCGGACGATGTCCCGGACCCGGCTGCCATCTCCCCCTGCCCCCTCACCTGCGTTCTCGGCGGGCAGCTTCAGTGACCTGTTACGTCAGTTCGATCCGTCACTTTTTAATACATCGCTTTTTGATTCATTGCCTCCCTTCGGCGCTCACCATACAGAGGCTGCCACAGGCGAGTGGGATGAGGTGCAATCGGGTCTGCGGGCAGCCGACGCCCCCCCACCCACCATGCGCGTGGCTGTCACTGCCGCGCGGCCGCCGCGCGCCAAGCCGGCGCCGCGACGACGTGCTGCGCAACCCTCCGACGCTTCGCCGGCCGCGCAGGTGGATCTACGCACGCTCGGCTACAGCCAGCAGCAACAGGAGAAGATCAAACCGAAGGTTCGTTCGACAGTGGCGCAGCACCACGAGGCACTGGTCGGCCATGGGTTTACACACGCGCACATCGTTGCGCTCAGCCAACACCCGGCAGCGTTAGGGACCGTCGCTGTCAAGTATCAGGACATGATCGCAGCGTTGCCAGAGGCGACACACGAAGCGATCGTTGGCGTCGGCAAACAGTGGTCCGGCGCACGCGCTCTGGAGGCCTTGCTCACGGTGGCGGGAGAGTTGAGAGGTCCACCGTTACAGTTGGACACAGGCCAACTTCTCAAGATTGCAAAACGTGGCGGCGTGACCGCAGTGGAGGCAGTGCATGCATGGCGCAATGCACTGACGGGTGCCCCCCTGAACCTTACCCCGGAGCAGGTGGTGGCCATCGCCAGCXXXXXXGGTGGCAAGCAGGCGCTGGAGACGGTGCAGCGGCTGTTGCCGGTGCTGTGCCAGGCCCATGGCCTGACCCCGGAGCAGGTGGTGGCCATCGCCAGCAATGGCGGCGGCAAGCAGGCGCTGGAGACGGTGCAGCGGCTGTTGCCGGTGCTGTGCCAGGCCCATGGCCTGACACCGGAGCAGGTGGTGGCCATCGCCAGCAATAGCGGTGGCAAGCAGGCGCTGGAGACGGTGCAGCGGCTGTTGCCGGTGCTGTGCCAGGCCCATGGCCTCACCCCGGAGCAGGTGGTGGCCATCGCCAGCAATGGCGGCGGCAAGCAGGCGCTGGAGACGGTGCAGCGGCTGTTGCCGGTGCTGTGCCAGGCCCATGGCCTGACTCCGGAGCAGGTGGTGGCCATCGCCAGCAATATTGGCGGCAAGCAGGCGCTGGAGACGGTGCAGCGGCTGTTGCCGGTGCTGTGCCAGGCCCATGGCCTGACCCCGGAGCAGGTGGTGGCCATCGCCAGCAATATTGGCGGCAAGCAGGCGCTGGAGACGGTGCAGCGGCTGTTGCCGGTGCTGTGCCAGGCGCATGGCCTTACCCCGGAGCAGGTGGTGGCCATCGCCAGCAATATTGGTGGCAAGCAGGCGCTGGAGACGGTGCAGCGGCTGTTGCCGGTGCTGTGCCAGGCCCATGGCCTGACCCCGGAGCAGGTGGTGGCCATCGCCAGCCACGATGGCGGCAAGCAGGCGCTGGAGACGGTGCAGCGGCTGTTGCCGGTGCTGTGCCAGGCCCATGGCCTGACACCGGAGCAGGTGGTGGCCATCGCCAGCCACGATGGTGGCAAGCAGGCGCTGGAGACGGTGCAGCGGCTGTTGCCGGTGCTGTGCCAGGCCCATGGCCTCACCCCGGAGCAGGTGGTGGCCATCGCCAGCAATGGCGGCGGCAAGCAGGCGCTGGAGACGGTGCAGCGGCTGTTGCCGGTGCTGTGCCAGGCCCATGGCCTGACTCCGGAGCAGGTGGTGGCCATCGCCAGCAATAGCGGCGGCAAGCAGGCGCTGGAGACGGTGCAGCGGCTGTTGCCGGTGCTGTGCCAGGCCCATGGCCTGACCCCGGAGCAGGTGGTGGCCATCGCCAGCAATAGCGGCGGCAAGCAGGCGCTGGAGAGCATTGTTGCCCAGTTATCTCGCCCTGATCCGGCGTTGGCCGCGTTGACCAACGACCACCTCGTCGCCTTGGCCTGCCTCGGCGGACGTCCTGCGCTGGATGCAGTGAAAAAGGGATTGCCGCACGCGCCGGCCTTGATCAAAAGAACCAATCGCCGTATTCCCGAACGCACATCCCATCGCGTTGCCGACCACGCGCAAGTGGTTCGCGTGCTGGGTTTTTTCCAGTGCCACTCCCACCCAGCGCAAGCATTTGATGACGCCATGACGCAGTTCGGGATGAGCAGGCACGGGTTGTTACAGCTCTTTCGCAGAGTGGGCGTCACCGAACTCGAAGCCCGCAGTGGAACGCTCCCCCCAGCCTCGCAGCGTTGGGACCGTATCCTCCAGGCATCAGGGATGAAAAGGGCCAAACCGTCCCCTACTTCAACTCAAACGCCGGATCAGGCGTCTTTGCATGCATTCGCCGATTCGCTGGAGCGTGACCTTGATGCGCCTAGCCCAATGCACGAGGGAGATCAGACGCGGGCAAGCAGCCGTAAACGGTCCCGATCGGATCGTGCTGTCACCGGTCCCTCCGCACAGCAATCGTTCGAGGTGCGCGTTCCCGAACAGCGCGATGCGCTGCATTTGCCCCTCAGTTGGAGGGTAAAACGCCCGCGTACCAGTATCGGGGGCGGCCTCCCGGATCCTGGTACGCCCACGGCTGCCGACCTGGCAGCGTCCAGCACCGTGATGCGGGAACAAGATGAGGACCCCTTCGCAGGGGCAGCGGATGATTTCCCGGCATTCAACGAAGAGGAGCTCGCATGGTTGATGGAGCTATTGCCTCAGTGAAGCTT

**Amino acid sequence dAvrBs3(RVD1=XX) (11.5 repeats)**

MGLINGEQKLISEEDLNGEQKLISEEDLNGLDGEQKLISEEDLNGEQKLISEEDLNGSHMDPIRSRTPSPARELLPGPQPDGVQPTADRGVSPPAGGPLDGLPARRTMSRTRLPSPPAPSPAFSAGSFSDLLRQFDPSLFNTSLFDSLPPFGAHHTEAATGEWDEVQSGLRAADAPPPTMRVAVTAARPPRAKPAPRRRAAQPSDASPAAQVDLRTLGYSQQQQEKIKPKVRSTVAQHHEALVGHGFTHAHIVALSQHPAALGTVAVKYQDMIAALPEA

-1 THEAIVGVGKQWSGARALEALLTVAGELRGPPLQ

00 LDTGQLLKIAKRGGVTAVEAVHAWRNALTGAPLN

01 LTPEQVVAIASXXGGKQALETVQRLLPVLCQAHG

02 LTPEQVVAIASNGGGKQALETVQRLLPVLCQAHG

03 LTPEQVVAIASNSGGKQALETVQRLLPVLCQAHG

04 LTPEQVVAIASNGGGKQALETVQRLLPVLCQAHG

05 LTPEQVVAIASNIGGKQALETVQRLLPVLCQAHG

06 LTPEQVVAIASNIGGKQALETVQRLLPVLCQAHG

07 LTPEQVVAIASNIGGKQALETVQRLLPVLCQAHG

08 LTPEQVVAIASHDGGKQALETVQRLLPVLCQAHG

09 LTPEQVVAIASHDGGKQALETVQRLLPVLCQAHG

10 LTPEQVVAIASNGGGKQALETVQRLLPVLCQAHG

11 LTPEQVVAIASNSGGKQALETVQRLLPVLCQAHG

12 LTPEQVVAIASNSGGKQALESIVA

QLSRPDPALAALTNDHLVALACLGGRPALDAVKKGLPHAPALIKRTNRRIPERTSHRVADHAQVVRVLGFFQCHSHPAQAFDDAMTQFGMSRHGLLQLFRRVGVTELEARSGTLPPASQRWDRILQASGMKRAKPSPTSTQTPDQASLHAFADSLERDLDAPSPMHEGDQTRASSRKRSRSDRAVTGPSAQQSFEVRVPEQRDALHLPLSWRVKRPRTSIGGGLPDPGTPTAADLAASSTVMREQDEDPFAGAADDFPAFNEEELAWLMELLPQ*

**DNA sequence designer-*avrBs3*(RVD1=XX) (10.5 repeats)**

**ATG**GGGTTAATTAACGGTGAACAAAAGCTAATCTCCGAGGAAGACTTGAACGGTGAACAAAAATTAATCTCAGAAGAAGACTTGAACGGACTCGACGGTGAACAAAAGTTGATTTCTGAAGAAGATTTGAACGGTGAACAAAAGCTAATCTCCGAGGAAGACTTGAACGGTAGCCATATGGATCCCATTCGTTCGCGCACACCAAGTCCTGCCCGCGAGCTTCTGCCCGGACCCCAACCCGATGGGGTTCAGCCGACTGCAGATCGTGGGGTGTCTCCGCCTGCCGGCGGCCCCCTGGATGGCTTGCCCGCTCGGCGGACGATGTCCCGGACCCGGCTGCCATCTCCCCCTGCCCCCTCACCTGCGTTCTCGGCGGGCAGCTTCAGTGACCTGTTACGTCAGTTCGATCCGTCACTTTTTAATACATCGCTTTTTGATTCATTGCCTCCCTTCGGCGCTCACCATACAGAGGCTGCCACAGGCGAGTGGGATGAGGTGCAATCGGGTCTGCGGGCAGCCGACGCCCCCCCACCCACCATGCGCGTGGCTGTCACTGCCGCGCGGCCGCCGCGCGCCAAGCCGGCGCCGCGACGACGTGCTGCGCAACCCTCCGACGCTTCGCCGGCCGCGCAGGTGGATCTACGCACGCTCGGCTACAGCCAGCAGCAACAGGAGAAGATCAAACCGAAGGTTCGTTCGACAGTGGCGCAGCACCACGAGGCACTGGTCGGCCATGGGTTTACACACGCGCACATCGTTGCGCTCAGCCAACACCCGGCAGCGTTAGGGACCGTCGCTGTCAAGTATCAGGACATGATCGCAGCGTTGCCAGAGGCGACACACGAAGCGATCGTTGGCGTCGGCAAACAGTGGTCCGGCGCACGCGCTCTGGAGGCCTTGCTCACGGTGGCGGGAGAGTTGAGAGGTCCACCGTTACAGTTGGACACAGGCCAACTTCTCAAGATTGCAAAACGTGGCGGCGTGACCGCAGTGGAGGCAGTGCATGCATGGCGCAATGCACTGACGGGTGCCCCCCTGAACCTTACCCCGGAGCAGGTGGTGGCCATCGCCAGCXXXXXXGGTGGCAAGCAGGCGCTGGAGACGGTGCAGCGGCTGTTGCCGGTGCTGTGCCAGGCCCATGGCCTGACCCCGGAGCAGGTGGTGGCCATCGCCAGCAATGGCGGCGGCAAGCAGGCGCTGGAGACGGTGCAGCGGCTGTTGCCGGTGCTGTGCCAGGCCCATGGCCTGACACCGGAGCAGGTGGTGGCCATCGCCAGCAATAGCGGTGGCAAGCAGGCGCTGGAGACGGTGCAGCGGCTGTTGCCGGTGCTGTGCCAGGCCCATGGCCTCACCCCGGAGCAGGTGGTGGCCATCGCCAGCAATGGCGGCGGCAAGCAGGCGCTGGAGACGGTGCAGCGGCTGTTGCCGGTGCTGTGCCAGGCCCATGGCCTGACTCCGGAGCAGGTGGTGGCCATCGCCAGCAATATTGGCGGCAAGCAGGCGCTGGAGACGGTGCAGCGGCTGTTGCCGGTGCTGTGCCAGGCCCATGGCCTGACCCCGGAGCAGGTGGTGGCCATCGCCAGCAATATTGGCGGCAAGCAGGCGCTGGAGACGGTGCAGCGGCTGTTGCCGGTGCTGTGCCAGGCGCATGGCCTTACCCCGGAGCAGGTGGTGGCCATCGCCAGCAATATTGGTGGCAAGCAGGCGCTGGAGACGGTGCAGCGGCTGTTGCCGGTGCTGTGCCAGGCCCATGGCCTGACCCCGGAGCAGGTGGTGGCCATCGCCAGCCACGATGGCGGCAAGCAGGCGCTGGAGACGGTGCAGCGGCTGTTGCCGGTGCTGTGCCAGGCCCATGGCCTGACACCGGAGCAGGTGGTGGCCATCGCCAGCCACGATGGTGGCAAGCAGGCGCTGGAGACGGTGCAGCGGCTGTTGCCGGTGCTGTGCCAGGCCCATGGCCTCACCCCGGAGCAGGTGGTGGCCATCGCCAGCAATGGCGGCGGCAAGCAGGCGCTGGAGACGGTGCAGCGGCTGTTGCCGGTGCTGTGCCAGGCCCATGGCCTGACTCCGGAGCAGGTGGTGGCCATCGCCAGCAATAGCGGCGGCAAGCAGGCGCTGGAGAGCATTGTTGCCCAGTTATCTCGCCCTGATCCGGCGTTGGCCGCGTTGACCAACGACCACCTCGTCGCCTTGGCCTGCCTCGGCGGACGTCCTGCGCTGGATGCAGTGAAAAAGGGATTGCCGCACGCGCCGGCCTTGATCAAAAGAACCAATCGCCGTATTCCCGAACGCACATCCCATCGCGTTGCCGACCACGCGCAAGTGGTTCGCGTGCTGGGTTTTTTCCAGTGCCACTCCCACCCAGCGCAAGCATTTGATGACGCCATGACGCAGTTCGGGATGAGCAGGCACGGGTTGTTACAGCTCTTTCGCAGAGTGGGCGTCACCGAACTCGAAGCCCGCAGTGGAACGCTCCCCCCAGCCTCGCAGCGTTGGGACCGTATCCTCCAGGCATCAGGGATGAAAAGGGCCAAACCGTCCCCTACTTCAACTCAAACGCCGGATCAGGCGTCTTTGCATGCATTCGCCGATTCGCTGGAGCGTGACCTTGATGCGCCTAGCCCAATGCACGAGGGAGATCAGACGCGGGCAAGCAGCCGTAAACGGTCCCGATCGGATCGTGCTGTCACCGGTCCCTCCGCACAGCAATCGTTCGAGGTGCGCGTTCCCGAACAGCGCGATGCGCTGCATTTGCCCCTCAGTTGGAGGGTAAAACGCCCGCGTACCAGTATCGGGGGCGGCCTCCCGGATCCTGGTACGCCCACGGCTGCCGACCTGGCAGCGTCCAGCACCGTGATGCGGGAACAAGATGAGGACCCCTTCGCAGGGGCAGCGGATGATTTCCCGGCATTCAACGAAGAGGAGCTCGCATGGTTGATGGAGCTATTGCCTCAGTGAAGCTT

**Amino acid sequence dAvrBs3(RVD1=XX) (10.5 repeats)**

MGLINGEQKLISEEDLNGEQKLISEEDLNGLDGEQKLISEEDLNGEQKLISEEDLNGSHMDPIRSRTPSPARELLPGPQPDGVQPTADRGVSPPAGGPLDGLPARRTMSRTRLPSPPAPSPAFSAGSFSDLLRQFDPSLFNTSLFDSLPPFGAHHTEAATGEWDEVQSGLRAADAPPPTMRVAVTAARPPRAKPAPRRRAAQPSDASPAAQVDLRTLGYSQQQQEKIKPKVRSTVAQHHEALVGHGFTHAHIVALSQHPAALGTVAVKYQDMIAALPEA

-1 THEAIVGVGKQWSGARALEALLTVAGELRGPPLQ

00 LDTGQLLKIAKRGGVTAVEAVHAWRNALTGAPLN

01 LTPEQVVAIASXXGGKQALETVQRLLPVLCQAHG

02 LTPEQVVAIASNGGGKQALETVQRLLPVLCQAHG

03 LTPEQVVAIASNSGGKQALETVQRLLPVLCQAHG

04 LTPEQVVAIASNGGGKQALETVQRLLPVLCQAHG

05 LTPEQVVAIASNIGGKQALETVQRLLPVLCQAHG

06 LTPEQVVAIASNIGGKQALETVQRLLPVLCQAHG

07 LTPEQVVAIASNIGGKQALETVQRLLPVLCQAHG

08 LTPEQVVAIASHDGGKQALETVQRLLPVLCQAHG

09 LTPEQVVAIASHDGGKQALETVQRLLPVLCQAHG

10 LTPEQVVAIASNGGGKQALETVQRLLPVLCQAHG

11 LTPEQVVAIASNSGGKQALESIVA

QLSRPDPALAALTNDHLVALACLGGRPALDAVKKGLPHAPALIKRTNRRIPERTSHRVADHAQVVRVLGFFQCHSHPAQAFDDAMTQFGMSRHGLLQLFRRVGVTELEARSGTLPPASQRWDRILQASGMKRAKPSPTSTQTPDQASLHAFADSLERDLDAPSPMHEGDQTRASSRKRSRSDRAVTGPSAQQSFEVRVPEQRDALHLPLSWRVKRPRTSIGGGLPDPGTPTAADLAASSTVMREQDEDPFAGAADDFPAFNEEELAWLMELLPQ*

**DNA sequence designer-*avrBs3*(RVD1=XX) (9.5 repeats)**

**ATG**GGGTTAATTAACGGTGAACAAAAGCTAATCTCCGAGGAAGACTTGAACGGTGAACAAAAATTAATCTCAGAAGAAGACTTGAACGGACTCGACGGTGAACAAAAGTTGATTTCTGAAGAAGATTTGAACGGTGAACAAAAGCTAATCTCCGAGGAAGACTTGAACGGTAGCCATATGGATCCCATTCGTTCGCGCACACCAAGTCCTGCCCGCGAGCTTCTGCCCGGACCCCAACCCGATGGGGTTCAGCCGACTGCAGATCGTGGGGTGTCTCCGCCTGCCGGCGGCCCCCTGGATGGCTTGCCCGCTCGGCGGACGATGTCCCGGACCCGGCTGCCATCTCCCCCTGCCCCCTCACCTGCGTTCTCGGCGGGCAGCTTCAGTGACCTGTTACGTCAGTTCGATCCGTCACTTTTTAATACATCGCTTTTTGATTCATTGCCTCCCTTCGGCGCTCACCATACAGAGGCTGCCACAGGCGAGTGGGATGAGGTGCAATCGGGTCTGCGGGCAGCCGACGCCCCCCCACCCACCATGCGCGTGGCTGTCACTGCCGCGCGGCCGCCGCGCGCCAAGCCGGCGCCGCGACGACGTGCTGCGCAACCCTCCGACGCTTCGCCGGCCGCGCAGGTGGATCTACGCACGCTCGGCTACAGCCAGCAGCAACAGGAGAAGATCAAACCGAAGGTTCGTTCGACAGTGGCGCAGCACCACGAGGCACTGGTCGGCCATGGGTTTACACACGCGCACATCGTTGCGCTCAGCCAACACCCGGCAGCGTTAGGGACCGTCGCTGTCAAGTATCAGGACATGATCGCAGCGTTGCCAGAGGCGACACACGAAGCGATCGTTGGCGTCGGCAAACAGTGGTCCGGCGCACGCGCTCTGGAGGCCTTGCTCACGGTGGCGGGAGAGTTGAGAGGTCCACCGTTACAGTTGGACACAGGCCAACTTCTCAAGATTGCAAAACGTGGCGGCGTGACCGCAGTGGAGGCAGTGCATGCATGGCGCAATGCACTGACGGGTGCCCCCCTGAACCTTACCCCGGAGCAGGTGGTGGCCATCGCCAGCXXXXXXGGTGGCAAGCAGGCGCTGGAGACGGTGCAGCGGCTGTTGCCGGTGCTGTGCCAGGCCCATGGCCTGACCCCGGAGCAGGTGGTGGCCATCGCCAGCAATGGCGGCGGCAAGCAGGCGCTGGAGACGGTGCAGCGGCTGTTGCCGGTGCTGTGCCAGGCCCATGGCCTGACACCGGAGCAGGTGGTGGCCATCGCCAGCAATAGCGGTGGCAAGCAGGCGCTGGAGACGGTGCAGCGGCTGTTGCCGGTGCTGTGCCAGGCCCATGGCCTCACCCCGGAGCAGGTGGTGGCCATCGCCAGCAATGGCGGCGGCAAGCAGGCGCTGGAGACGGTGCAGCGGCTGTTGCCGGTGCTGTGCCAGGCCCATGGCCTGACTCCGGAGCAGGTGGTGGCCATCGCCAGCAATATTGGCGGCAAGCAGGCGCTGGAGACGGTGCAGCGGCTGTTGCCGGTGCTGTGCCAGGCCCATGGCCTGACCCCGGAGCAGGTGGTGGCCATCGCCAGCAATATTGGCGGCAAGCAGGCGCTGGAGACGGTGCAGCGGCTGTTGCCGGTGCTGTGCCAGGCGCATGGCCTTACCCCGGAGCAGGTGGTGGCCATCGCCAGCAATATTGGTGGCAAGCAGGCGCTGGAGACGGTGCAGCGGCTGTTGCCGGTGCTGTGCCAGGCCCATGGCCTGACCCCGGAGCAGGTGGTGGCCATCGCCAGCCACGATGGCGGCAAGCAGGCGCTGGAGACGGTGCAGCGGCTGTTGCCGGTGCTGTGCCAGGCCCATGGCCTGACACCGGAGCAGGTGGTGGCCATCGCCAGCCACGATGGCGGCAAGCAGGCGCTGGAGACGGTGCAGCGGCTGTTGCCGGTGCTGTGCCAGGCCCATGGCCTCACCCCGGAGCAGGTGGTGGCCATCGCCAGCAATGGCGGTGGCAAGCAGGCGCTGGAGAGCATTGTTGCCCAGTTATCTCGCCCTGATCCGGCGTTGGCCGCGTTGACCAACGACCACCTCGTCGCCTTGGCCTGCCTCGGCGGACGTCCTGCGCTGGATGCAGTGAAAAAGGGATTGCCGCACGCGCCGGCCTTGATCAAAAGAACCAATCGCCGTATTCCCGAACGCACATCCCATCGCGTTGCCGACCACGCGCAAGTGGTTCGCGTGCTGGGTTTTTTCCAGTGCCACTCCCACCCAGCGCAAGCATTTGATGACGCCATGACGCAGTTCGGGATGAGCAGGCACGGGTTGTTACAGCTCTTTCGCAGAGTGGGCGTCACCGAACTCGAAGCCCGCAGTGGAACGCTCCCCCCAGCCTCGCAGCGTTGGGACCGTATCCTCCAGGCATCAGGGATGAAAAGGGCCAAACCGTCCCCTACTTCAACTCAAACGCCGGATCAGGCGTCTTTGCATGCATTCGCCGATTCGCTGGAGCGTGACCTTGATGCGCCTAGCCCAATGCACGAGGGAGATCAGACGCGGGCAAGCAGCCGTAAACGGTCCCGATCGGATCGTGCTGTCACCGGTCCCTCCGCACAGCAATCGTTCGAGGTGCGCGTTCCCGAACAGCGCGATGCGCTGCATTTGCCCCTCAGTTGGAGGGTAAAACGCCCGCGTACCAGTATCGGGGGCGGCCTCCCGGATCCTGGTACGCCCACGGCTGCCGACCTGGCAGCGTCCAGCACCGTGATGCGGGAACAAGATGAGGACCCCTTCGCAGGGGCAGCGGATGATTTCCCGGCATTCAACGAAGAGGAGCTCGCATGGTTGATGGAGCTATTGCCTCAGTGAAGCTT

**Amino acid sequence dAvrBs3(RVD1=XX) (9.5 repeats)**

MGLINGEQKLISEEDLNGEQKLISEEDLNGLDGEQKLISEEDLNGEQKLISEEDLNGSHMDPIRSRTPSPARELLPGPQPDGVQPTADRGVSPPAGGPLDGLPARRTMSRTRLPSPPAPSPAFSAGSFSDLLRQFDPSLFNTSLFDSLPPFGAHHTEAATGEWDEVQSGLRAADAPPPTMRVAVTAARPPRAKPAPRRRAAQPSDASPAAQVDLRTLGYSQQQQEKIKPKVRSTVAQHHEALVGHGFTHAHIVALSQHPAALGTVAVKYQDMIAALPEA

-1 THEAIVGVGKQWSGARALEALLTVAGELRGPPLQ

00 LDTGQLLKIAKRGGVTAVEAVHAWRNALTGAPLN

01 LTPEQVVAIASXXGGKQALETVQRLLPVLCQAHG

02 LTPEQVVAIASNGGGKQALETVQRLLPVLCQAHG

03 LTPEQVVAIASNSGGKQALETVQRLLPVLCQAHG

04 LTPEQVVAIASNGGGKQALETVQRLLPVLCQAHG

05 LTPEQVVAIASNIGGKQALETVQRLLPVLCQAHG

06 LTPEQVVAIASNIGGKQALETVQRLLPVLCQAHG

07 LTPEQVVAIASNIGGKQALETVQRLLPVLCQAHG

08 LTPEQVVAIASHDGGKQALETVQRLLPVLCQAHG

09 LTPEQVVAIASHDGGKQALETVQRLLPVLCQAHG

10 LTPEQVVAIASNGGGKQALESIVA

QLSRPDPALAALTNDHLVALACLGGRPALDAVKKGLPHAPALIKRTNRRIPERTSHRVADHAQVVRVLGFFQCHSHPAQAFDDAMTQFGMSRHGLLQLFRRVGVTELEARSGTLPPASQRWDRILQASGMKRAKPSPTSTQTPDQASLHAFADSLERDLDAPSPMHEGDQTRASSRKRSRSDRAVTGPSAQQSFEVRVPEQRDALHLPLSWRVKRPRTSIGGGLPDPGTPTAADLAASSTVMREQDEDPFAGAADDFPAFNEEELAWLMELLPQ*

**DNA sequence *c-myc-artrep18-1* (RVD1 = XX)**

**ATG**GGGTTAATTAACGGTGAACAAAAGCTAATCTCCGAGGAAGACTTGAACGGTGAACAAAAATTAATCTCAGAAGAAGACTTGAACGGACTCGACGGTGAACAAAAGTTGATTTCTGAAGAAGATTTGAACGGTGAACAAAAGCTAATCTCCGAGGAAGACTTGAACGGTAGCCATATGGATCCCATTCGTTCGCGCACACCAAGTCCTGCCCGCGAGCTTCTGCCCGGACCCCAACCCGATGGGGTTCAGCCGACTGCAGATCGTGGGGTGTCTCCGCCTGCCGGCGGCCCCCTGGATGGCTTGCCCGCTCGGCGGACGATGTCCCGGACCCGGCTGCCATCTCCCCCTGCCCCCTCACCTGCGTTCTCGGCGGGCAGCTTCAGTGACCTGTTACGTCAGTTCGATCCGTCACTTTTTAATACATCGCTTTTTGATTCATTGCCTCCCTTCGGCGCTCACCATACAGAGGCTGCCACAGGCGAGTGGGATGAGGTGCAATCGGGTCTGCGGGCAGCCGACGCCCCCCCACCCACCATGCGCGTGGCTGTCACTGCCGCGCGGCCGCCGCGCGCCAAGCCGGCGCCGCGACGACGTGCTGCGCAACCCTCCGACGCTTCGCCGGCCGCGCAGGTGGATCTACGCACGCTCGGCTACAGCCAGCAGCAACAGGAGAAGATCAAACCGAAGGTTCGTTCGACAGTGGCGCAGCACCACGAGGCACTGGTCGGCCATGGGTTTACACACGCGCACATCGTTGCGCTCAGCCAACACCCGGCAGCGTTAGGGACCGTCGCTGTCAAGTATCAGGACATGATCGCAGCGTTGCCAGAGGCGACACACGAAGCGATCGTTGGCGTCGGCAAACAGTGGTCCGGCGCACGCGCTCTGGAGGCCTTGCTCACGGTGGCGGGAGAGTTGAGAGGTCCACCGTTACAGTTGGACACAGGCCAACTTCTCAAGATTGCAAAACGTGGCGGCGTGACCGCAGTGGAGGCAGTGCATGCATGGCGCAATGCACTGACGGGTGCCCCCCTGAACCTTACCCCGGAGCAGGTGGTGGCCATCGCCAGCXXXXXXGGTGGCAAGCAGGCGCTGGAGACGGTGCAGCGGCTGTTGCCGGTGCTGTGCCAGGCCCATGGCCTGACCCCGGAGCAGGTGGTGGCCATCGCCAGCAATATTGGCGGCAAGCAGGCGCTGGAGACGGTGCAGCGGCTGTTGCCGGTGCTGTGCCAGGCCCATGGCCTGACACCGGAGCAGGTGGTGGCCATCGCCAGCCACGATGGTGGCAAGCAGGCGCTGGAGACGGTGCAGCGGCTGTTGCCGGTGCTGTGCCAGGCCCATGGCCTCACCCCGGAGCAGGTGGTGGCCATCGCCAGCAATATTGGCGGCAAGCAGGCGCTGGAGACGGTGCAGCGGCTGTTGCCGGTGCTGTGCCAGGCCCATGGCCTGACTCCGGAGCAGGTGGTGGCCATCGCCAGCCACGATGGCGGCAAGCAGGCGCTGGAGACGGTGCAGCGGCTGTTGCCGGTGCTGTGCCAGGCCCATGGCCTGACCCCGGAGCAGGTGGTGGCCATCGCCAGCAATATTGGCGGCAAGCAGGCGCTGGAGACGGTGCAGCGGCTGTTGCCGGTGCTGTGCCAGGCGCATGGCCTTACCCCGGAGCAGGTGGTGGCCATCGCCAGCCACGATGGTGGCAAGCAGGCGCTGGAGACGGTGCAGCGGCTGTTGCCGGTGCTGTGCCAGGCCCATGGCCTGACCCCGGAGCAGGTGGTGGCCATCGCCAGCCACGATGGCGGCAAGCAGGCGCTGGAGACGGTGCAGCGGCTGTTGCCGGTGCTGTGCCAGGCCCATGGCCTGACACCGGAGCAGGTGGTGGCCATCGCCAGCAATATTGGTGGCAAGCAGGCGCTGGAGACGGTGCAGCGGCTGTTGCCGGTGCTGTGCCAGGCCCATGGCCTCACCCCGGAGCAGGTGGTGGCCATCGCCAGCAATGGCGGCGGCAAGCAGGCGCTGGAGACGGTGCAGCGGCTGTTGCCGGTGCTGTGCCAGGCCCATGGCCTGACTCCGGAGCAGGTGGTGGCCATCGCCAGCAATATTGGCGGCAAGCAGGCGCTGGAGACGGTGCAGCGGCTGTTGCCGGTGCTGTGCCAGGCCCATGGCCTGACCCCGGAGCAGGTGGTGGCCATCGCCAGCAATATTGGCGGCAAGCAGGCGCTGGAGACGGTGCAGCGGCTGTTGCCGGTGCTGTGCCAGGCGCATGGCCTTACCCCGGAGCAGGTGGTGGCCATCGCCAGCAATAATGGTGGCAAGCAGGCGCTGGAGACGGTGCAGCGGCTGTTGCCGGTGCTGTGCCAGGCCCATGGCCTGACCCCGGAGCAGGTGGTGGCCATCGCCAGCAATAATGGCGGCAAGCAGGCGCTGGAGACGGTGCAGCGGCTGTTGCCGGTGCTGTGCCAGGCCCATGGCCTGACACCGGAGCAGGTGGTGGCCATCGCCAGCAATAATGGTGGCAAGCAGGCGCTGGAGACGGTGCAGCGGCTGTTGCCGGTGCTGTGCCAGGCCCATGGCCTCACCCCGGAGCAGGTGGTGGCCATCGCCAGCCACGATGGCGGCAAGCAGGCGCTGGAGACGGTGCAGCGGCTGTTGCCGGTGCTGTGCCAGGCCCATGGCCTGACTCCGGAGCAGGTGGTGGCCATCGCCAGCAATATTGGCGGCAAGCAGGCGCTGGAGACGGTGCAGCGGCTGTTGCCGGTGCTGTGCCAGGCCCATGGCCTGACCCCGGAGCAGGTGGTGGCCATCGCCAGCAATGGCGGCGGCAAGCAGGCGCTGGAGAGCATTGTTGCCCAGTTATCTCGCCCTGATCCGGCGTTGGCCGCGTTGACCAACGACCACCTCGTCGCCTTGGCCTGCCTCGGCGGACGTCCTGCGCTGGATGCAGTGAAAAAGGGATTGCCGCACGCGCCGGCCTTGATCAAAAGAACCAATCGCCGTATTCCCGAACGCACATCCCATCGCGTTGCCGACCACGCGCAAGTGGTTCGCGTGCTGGGTTTTTTCCAGTGCCACTCCCACCCAGCGCAAGCATTTGATGACGCCATGACGCAGTTCGGGATGAGCAGGCACGGGTTGTTACAGCTCTTTCGCAGAGTGGGCGTCACCGAACTCGAAGCCCGCAGTGGAACGCTCCCCCCAGCCTCGCAGCGTTGGGACCGTATCCTCCAGGCATCAGGGATGAAAAGGGCCAAACCGTCCCCTACTTCAACTCAAACGCCGGATCAGGCGTCTTTGCATGCATTCGCCGATTCGCTGGAGCGTGACCTTGATGCGCCTAGCCCAATGCACGAGGGAGATCAGACGCGGGCAAGCAGCCGTAAACGGTCCCGATCGGATCGTGCTGTCACCGGTCCCTCCGCACAGCAATCGTTCGAGGTGCGCGTTCCCGAACAGCGCGATGCGCTGCATTTGCCCCTCAGTTGGAGGGTAAAACGCCCGCGTACCAGTATCGGGGGCGGCCTCCCGGATCCTGGTACGCCCACGGCTGCCGACCTGGCAGCGTCCAGCACCGTGATGCGGGAACAAGATGAGGACCCCTTCGCAGGGGCAGCGGATGATTTCCCGGCATTCAACGAAGAGGAGCTCGCATGGTTGATGGAGCTATTGCCTCAGTGAAGCTT

**RVD1 = codon usage**

HD = CAC GAT

NS = AAT AGC

**Amino acid sequence dARTrep18-1(RVD1=XX)**

MGLINGEQKLISEEDLNGEQKLISEEDLNGLDGEQKLISEEDLNGEQKLISEEDLNGSHMDPIRSRTPSPARELLPGPQPDGVQPTADRGVSPPAGGPLDGLPARRTMSRTRLPSPPAPSPAFSAGSFSDLLRQFDPSLFNTSLFDSLPPFGAHHTEAATGEWDEVQSGLRAADAPPPTMRVAVTAARPPRAKPAPRRRAAQPSDASPAAQVDLRTLGYSQQQQEKIKPKVRSTVAQHHEALVGHGFTHAHIVALSQHPAALGTVAVKYQDMIAALPEA

-1 THEAIVGVGKQWSGARALEALLTVAGELRGPPLQ

00 LDTGQLLKIAKRGGVTAVEAVHAWRNALTGAPLN

01 LTPEQVVAIASXXGGKQALETVQRLLPVLCQAHG

02 LTPEQVVAIASNIGGKQALETVQRLLPVLCQAHG

03 LTPEQVVAIASHDGGKQALETVQRLLPVLCQAHG

04 LTPEQVVAIASNIGGKQALETVQRLLPVLCQAHG

05 LTPEQVVAIASHDGGKQALETVQRLLPVLCQAHG

06 LTPEQVVAIASNIGGKQALETVQRLLPVLCQAHG

07 LTPEQVVAIASHDGGKQALETVQRLLPVLCQAHG

08 LTPEQVVAIASHDGGKQALETVQRLLPVLCQAHG

09 LTPEQVVAIASNIGGKQALETVQRLLPVLCQAHG

10 LTPEQVVAIASNGGGKQALETVQRLLPVLCQAHG

11 LTPEQVVAIASNIGGKQALETVQRLLPVLCQAHG

12 LTPEQVVAIASNIGGKQALETVQRLLPVLCQAHG

13 LTPEQVVAIASNNGGKQALETVQRLLPVLCQAHG

14 LTPEQVVAIASNNGGKQALETVQRLLPVLCQAHG

15 LTPEQVVAIASNNGGKQALETVQRLLPVLCQAHG

16 LTPEQVVAIASHDGGKQALETVQRLLPVLCQAHG

17 LTPEQVVAIASNIGGKQALETVQRLLPVLCQAHG

18 LTPEQVVAIASNGGGKQALESIVA

QLSRPDPALAALTNDHLVALACLGGRPALDAVKKGLPHAPALIKRTNRRIPERTSHRVADHAQVVRVLGFFQCHSHPAQAFDDAMTQFGMSRHGLLQLFRRVGVTELEARSGTLPPASQRWDRILQASGMKRAKPSPTSTQTPDQASLHAFADSLERDLDAPSPMHEGDQTRASSRKRSRSDRAVTGPSAQQSFEVRVPEQRDALHLPLSWRVKRPRTSIGGGLPDPGTPTAADLAASSTVMREQDEDPFAGAADDFPAFNEEELAWLMELLPQ*

**DNA sequence *c-myc-artrep18-2* (RVD1 = XX)**

**ATG**GGGTTAATTAACGGTGAACAAAAGCTAATCTCCGAGGAAGACTTGAACGGTGAACAAAAATTAATCTCAGAAGAAGACTTGAACGGACTCGACGGTGAACAAAAGTTGATTTCTGAAGAAGATTTGAACGGTGAACAAAAGCTAATCTCCGAGGAAGACTTGAACGGTAGCCATATGGATCCCATTCGTTCGCGCACACCAAGTCCTGCCCGCGAGCTTCTGCCCGGACCCCAACCCGATGGGGTTCAGCCGACTGCAGATCGTGGGGTGTCTCCGCCTGCCGGCGGCCCCCTGGATGGCTTGCCCGCTCGGCGGACGATGTCCCGGACCCGGCTGCCATCTCCCCCTGCCCCCTCACCTGCGTTCTCGGCGGGCAGCTTCAGTGACCTGTTACGTCAGTTCGATCCGTCACTTTTTAATACATCGCTTTTTGATTCATTGCCTCCCTTCGGCGCTCACCATACAGAGGCTGCCACAGGCGAGTGGGATGAGGTGCAATCGGGTCTGCGGGCAGCCGACGCCCCCCCACCCACCATGCGCGTGGCTGTCACTGCCGCGCGGCCGCCGCGCGCCAAGCCGGCGCCGCGACGACGTGCTGCGCAACCCTCCGACGCTTCGCCGGCCGCGCAGGTGGATCTACGCACGCTCGGCTACAGCCAGCAGCAACAGGAGAAGATCAAACCGAAGGTTCGTTCGACAGTGGCGCAGCACCACGAGGCACTGGTCGGCCATGGGTTTACACACGCGCACATCGTTGCGCTCAGCCAACACCCGGCAGCGTTAGGGACCGTCGCTGTCAAGTATCAGGACATGATCGCAGCGTTGCCAGAGGCGACACACGAAGCGATCGTTGGCGTCGGCAAACAGTGGTCCGGCGCACGCGCTCTGGAGGCCTTGCTCACGGTGGCGGGAGAGTTGAGAGGTCCACCGTTACAGTTGGACACAGGCCAACTTCTCAAGATTGCAAAACGTGGCGGCGTGACCGCAGTGGAGGCAGTGCATGCATGGCGCAATGCACTGACGGGTGCCCCCCTGAACCTTACCCCGGAGCAGGTGGTGGCCATCGCCAGCXXXXXXGGTGGCAAGCAGGCGCTGGAGACGGTGCAGCGGCTGTTGCCGGTGCTGTGCCAGGCCCATGGCCTGACCCCGGAGCAGGTGGTGGCCATCGCCAGCAATATTGGCGGCAAGCAGGCGCTGGAGACGGTGCAGCGGCTGTTGCCGGTGCTGTGCCAGGCCCATGGCCTGACACCGGAGCAGGTGGTGGCCATCGCCAGCAATGGCGGTGGCAAGCAGGCGCTGGAGACGGTGCAGCGGCTGTTGCCGGTGCTGTGCCAGGCCCATGGCCTCACCCCGGAGCAGGTGGTGGCCATCGCCAGCAATAATGGCGGCAAGCAGGCGCTGGAGACGGTGCAGCGGCTGTTGCCGGTGCTGTGCCAGGCCCATGGCCTGACTCCGGAGCAGGTGGTGGCCATCGCCAGCAATGGCGGCGGCAAGCAGGCGCTGGAGACGGTGCAGCGGCTGTTGCCGGTGCTGTGCCAGGCCCATGGCCTGACCCCGGAGCAGGTGGTGGCCATCGCCAGCAATAATGGCGGCAAGCAGGCGCTGGAGACGGTGCAGCGGCTGTTGCCGGTGCTGTGCCAGGCGCATGGCCTTACCCCGGAGCAGGTGGTGGCCATCGCCAGCAATGGCGGTGGCAAGCAGGCGCTGGAGACGGTGCAGCGGCTGTTGCCGGTGCTGTGCCAGGCCCATGGCCTGACCCCGGAGCAGGTGGTGGCCATCGCCAGCAATAATGGCGGCAAGCAGGCGCTGGAGACGGTGCAGCGGCTGTTGCCGGTGCTGTGCCAGGCCCATGGCCTGACACCGGAGCAGGTGGTGGCCATCGCCAGCCACGATGGTGGCAAGCAGGCGCTGGAGACGGTGCAGCGGCTGTTGCCGGTGCTGTGCCAGGCCCATGGCCTCACCCCGGAGCAGGTGGTGGCCATCGCCAGCCACGATGGCGGCAAGCAGGCGCTGGAGACGGTGCAGCGGCTGTTGCCGGTGCTGTGCCAGGCCCATGGCCTGACTCCGGAGCAGGTGGTGGCCATCGCCAGCAATGGCGGCGGCAAGCAGGCGCTGGAGACGGTGCAGCGGCTGTTGCCGGTGCTGTGCCAGGCCCATGGCCTGACCCCGGAGCAGGTGGTGGCCATCGCCAGCAATGGCGGCGGCAAGCAGGCGCTGGAGACGGTGCAGCGGCTGTTGCCGGTGCTGTGCCAGGCGCATGGCCTTACCCCGGAGCAGGTGGTGGCCATCGCCAGCAATGGCGGTGGCAAGCAGGCGCTGGAGACGGTGCAGCGGCTGTTGCCGGTGCTGTGCCAGGCCCATGGCCTGACCCCGGAGCAGGTGGTGGCCATCGCCAGCAATGGCGGCGGCAAGCAGGCGCTGGAGACGGTGCAGCGGCTGTTGCCGGTGCTGTGCCAGGCCCATGGCCTGACACCGGAGCAGGTGGTGGCCATCGCCAGCCACGATGGTGGCAAGCAGGCGCTGGAGACGGTGCAGCGGCTGTTGCCGGTGCTGTGCCAGGCCCATGGCCTCACCCCGGAGCAGGTGGTGGCCATCGCCAGCAATATTGGCGGCAAGCAGGCGCTGGAGACGGTGCAGCGGCTGTTGCCGGTGCTGTGCCAGGCCCATGGCCTGACTCCGGAGCAGGTGGTGGCCATCGCCAGCAATGGCGGCGGCAAGCAGGCGCTGGAGACGGTGCAGCGGCTGTTGCCGGTGCTGTGCCAGGCCCATGGCCTGACCCCGGAGCAGGTGGTGGCCATCGCCAGCAATGGCGGCGGCAAGCAGGCGCTGGAGAGCATTGTTGCCCAGTTATCTCGCCCTGATCCGGCGTTGGCCGCGTTGACCAACGACCACCTCGTCGCCTTGGCCTGCCTCGGCGGACGTCCTGCGCTGGATGCAGTGAAAAAGGGATTGCCGCACGCGCCGGCCTTGATCAAAAGAACCAATCGCCGTATTCCCGAACGCACATCCCATCGCGTTGCCGACCACGCGCAAGTGGTTCGCGTGCTGGGTTTTTTCCAGTGCCACTCCCACCCAGCGCAAGCATTTGATGACGCCATGACGCAGTTCGGGATGAGCAGGCACGGGTTGTTACAGCTCTTTCGCAGAGTGGGCGTCACCGAACTCGAAGCCCGCAGTGGAACGCTCCCCCCAGCCTCGCAGCGTTGGGACCGTATCCTCCAGGCATCAGGGATGAAAAGGGCCAAACCGTCCCCTACTTCAACTCAAACGCCGGATCAGGCGTCTTTGCATGCATTCGCCGATTCGCTGGAGCGTGACCTTGATGCGCCTAGCCCAATGCACGAGGGAGATCAGACGCGGGCAAGCAGCCGTAAACGGTCCCGATCGGATCGTGCTGTCACCGGTCCCTCCGCACAGCAATCGTTCGAGGTGCGCGTTCCCGAACAGCGCGATGCGCTGCATTTGCCCCTCAGTTGGAGGGTAAAACGCCCGCGTACCAGTATCGGGGGCGGCCTCCCGGATCCTGGTACGCCCACGGCTGCCGACCTGGCAGCGTCCAGCACCGTGATGCGGGAACAAGATGAGGACCCCTTCGCAGGGGCAGCGGATGATTTCCCGGCATTCAACGAAGAGGAGCTCGCATGGTTGATGGAGCTATTGCCTCAGTGAAGCTT

**RVD1 = codon usage**

HD = CAC GAT

NS = AAT AGC

**Amino acid sequence dARTrep18-2(RVD1=XX)**

MGLINGEQKLISEEDLNGEQKLISEEDLNGLDGEQKLISEEDLNGEQKLISEEDLNGSHMDPIRSRTPSPARELLPGPQPDGVQPTADRGVSPPAGGPLDGLPARRTMSRTRLPSPPAPSPAFSAGSFSDLLRQFDPSLFNTSLFDSLPPFGAHHTEAATGEWDEVQSGLRAADAPPPTMRVAVTAARPPRAKPAPRRRAAQPSDASPAAQVDLRTLGYSQQQQEKIKPKVRSTVAQHHEALVGHGFTHAHIVALSQHPAALGTVAVKYQDMIAALPEA

-1 THEAIVGVGKQWSGARALEALLTVAGELRGPPLQ

00 LDTGQLLKIAKRGGVTAVEAVHAWRNALTGAPLN

01 LTPEQVVAIASXXGGKQALETVQRLLPVLCQAHG

02 LTPEQVVAIASNIGGKQALETVQRLLPVLCQAHG

03 LTPEQVVAIASNGGGKQALETVQRLLPVLCQAHG

04 LTPEQVVAIASNNGGKQALETVQRLLPVLCQAHG

05 LTPEQVVAIASNGGGKQALETVQRLLPVLCQAHG

06 LTPEQVVAIASNNGGKQALETVQRLLPVLCQAHG

07 LTPEQVVAIASNGGGKQALETVQRLLPVLCQAHG

08 LTPEQVVAIASNNGGKQALETVQRLLPVLCQAHG

09 LTPEQVVAIASHDGGKQALETVQRLLPVLCQAHG

10 LTPEQVVAIASHDGGKQALETVQRLLPVLCQAHG

11 LTPEQVVAIASNGGGKQALETVQRLLPVLCQAHG

12 LTPEQVVAIASNGGGKQALETVQRLLPVLCQAHG

13 LTPEQVVAIASNGGGKQALETVQRLLPVLCQAHG

14 LTPEQVVAIASNGGGKQALETVQRLLPVLCQAHG

15 LTPEQVVAIASHDGGKQALETVQRLLPVLCQAHG

16 LTPEQVVAIASNIGGKQALETVQRLLPVLCQAHG

17 LTPEQVVAIASNGGGKQALETVQRLLPVLCQAHG

18 LTPEQVVAIASNGGGKQALESIVA

QLSRPDPALAALTNDHLVALACLGGRPALDAVKKGLPHAPALIKRTNRRIPERTSHRVADHAQVVRVLGFFQCHSHPAQAFDDAMTQFGMSRHGLLQLFRRVGVTELEARSGTLPPASQRWDRILQASGMKRAKPSPTSTQTPDQASLHAFADSLERDLDAPSPMHEGDQTRASSRKRSRSDRAVTGPSAQQSFEVRVPEQRDALHLPLSWRVKRPRTSIGGGLPDPGTPTAADLAASSTVMREQDEDPFAGAADDFPAFNEEELAWLMELLPQ*

**DNA sequence *c-myc-artrep18-3* (RVD1 = XX)**

**ATG**GGGTTAATTAACGGTGAACAAAAGCTAATCTCCGAGGAAGACTTGAACGGTGAACAAAAATTAATCTCAGAAGAAGACTTGAACGGACTCGACGGTGAACAAAAGTTGATTTCTGAAGAAGATTTGAACGGTGAACAAAAGCTAATCTCCGAGGAAGACTTGAACGGTAGCCATATGGATCCCATTCGTTCGCGCACACCAAGTCCTGCCCGCGAGCTTCTGCCCGGACCCCAACCCGATGGGGTTCAGCCGACTGCAGATCGTGGGGTGTCTCCGCCTGCCGGCGGCCCCCTGGATGGCTTGCCCGCTCGGCGGACGATGTCCCGGACCCGGCTGCCATCTCCCCCTGCCCCCTCACCTGCGTTCTCGGCGGGCAGCTTCAGTGACCTGTTACGTCAGTTCGATCCGTCACTTTTTAATACATCGCTTTTTGATTCATTGCCTCCCTTCGGCGCTCACCATACAGAGGCTGCCACAGGCGAGTGGGATGAGGTGCAATCGGGTCTGCGGGCAGCCGACGCCCCCCCACCCACCATGCGCGTGGCTGTCACTGCCGCGCGGCCGCCGCGCGCCAAGCCGGCGCCGCGACGACGTGCTGCGCAACCCTCCGACGCTTCGCCGGCCGCGCAGGTGGATCTACGCACGCTCGGCTACAGCCAGCAGCAACAGGAGAAGATCAAACCGAAGGTTCGTTCGACAGTGGCGCAGCACCACGAGGCACTGGTCGGCCATGGGTTTACACACGCGCACATCGTTGCGCTCAGCCAACACCCGGCAGCGTTAGGGACCGTCGCTGTCAAGTATCAGGACATGATCGCAGCGTTGCCAGAGGCGACACACGAAGCGATCGTTGGCGTCGGCAAACAGTGGTCCGGCGCACGCGCTCTGGAGGCCTTGCTCACGGTGGCGGGAGAGTTGAGAGGTCCACCGTTACAGTTGGACACAGGCCAACTTCTCAAGATTGCAAAACGTGGCGGCGTGACCGCAGTGGAGGCAGTGCATGCATGGCGCAATGCACTGACGGGTGCCCCCCTGAACCTTACCCCGGAGCAGGTGGTGGCCATCGCCAGCXXXXXXGGTGGCAAGCAGGCGCTGGAGACGGTGCAGCGGCTGTTGCCGGTGCTGTGCCAGGCCCATGGCCTGACCCCGGAGCAGGTGGTGGCCATCGCCAGCCACGATGGCGGCAAGCAGGCGCTGGAGACGGTGCAGCGGCTGTTGCCGGTGCTGTGCCAGGCCCATGGCCTGACACCGGAGCAGGTGGTGGCCATCGCCAGCAATATTGGTGGCAAGCAGGCGCTGGAGACGGTGCAGCGGCTGTTGCCGGTGCTGTGCCAGGCCCATGGCCTCACCCCGGAGCAGGTGGTGGCCATCGCCAGCCACGATGGCGGCAAGCAGGCGCTGGAGACGGTGCAGCGGCTGTTGCCGGTGCTGTGCCAGGCCCATGGCCTGACTCCGGAGCAGGTGGTGGCCATCGCCAGCCACGATGGCGGCAAGCAGGCGCTGGAGACGGTGCAGCGGCTGTTGCCGGTGCTGTGCCAGGCCCATGGCCTGACCCCGGAGCAGGTGGTGGCCATCGCCAGCCACGATGGCGGCAAGCAGGCGCTGGAGACGGTGCAGCGGCTGTTGCCGGTGCTGTGCCAGGCGCATGGCCTTACCCCGGAGCAGGTGGTGGCCATCGCCAGCAATAGCGGTGGCAAGCAGGCGCTGGAGACGGTGCAGCGGCTGTTGCCGGTGCTGTGCCAGGCCCATGGCCTGACCCCGGAGCAGGTGGTGGCCATCGCCAGCAATAGCGGCGGCAAGCAGGCGCTGGAGACGGTGCAGCGGCTGTTGCCGGTGCTGTGCCAGGCCCATGGCCTGACACCGGAGCAGGTGGTGGCCATCGCCAGCAATAGCGGTGGCAAGCAGGCGCTGGAGACGGTGCAGCGGCTGTTGCCGGTGCTGTGCCAGGCCCATGGCCTCACCCCGGAGCAGGTGGTGGCCATCGCCAGCCACGATGGCGGCAAGCAGGCGCTGGAGACGGTGCAGCGGCTGTTGCCGGTGCTGTGCCAGGCCCATGGCCTGACTCCGGAGCAGGTGGTGGCCATCGCCAGCAATATTGGCGGCAAGCAGGCGCTGGAGACGGTGCAGCGGCTGTTGCCGGTGCTGTGCCAGGCCCATGGCCTGACCCCGGAGCAGGTGGTGGCCATCGCCAGCAATGGCGGCGGCAAGCAGGCGCTGGAGACGGTGCAGCGGCTGTTGCCGGTGCTGTGCCAGGCGCATGGCCTTACCCCGGAGCAGGTGGTGGCCATCGCCAGCAATGGCGGTGGCAAGCAGGCGCTGGAGACGGTGCAGCGGCTGTTGCCGGTGCTGTGCCAGGCCCATGGCCTGACCCCGGAGCAGGTGGTGGCCATCGCCAGCAATGGCGGCGGCAAGCAGGCGCTGGAGACGGTGCAGCGGCTGTTGCCGGTGCTGTGCCAGGCCCATGGCCTGACACCGGAGCAGGTGGTGGCCATCGCCAGCCACGATGGTGGCAAGCAGGCGCTGGAGACGGTGCAGCGGCTGTTGCCGGTGCTGTGCCAGGCCCATGGCCTCACCCCGGAGCAGGTGGTGGCCATCGCCAGCAATGGCGGCGGCAAGCAGGCGCTGGAGACGGTGCAGCGGCTGTTGCCGGTGCTGTGCCAGGCCCATGGCCTGACTCCGGAGCAGGTGGTGGCCATCGCCAGCAATGGCGGCGGCAAGCAGGCGCTGGAGACGGTGCAGCGGCTGTTGCCGGTGCTGTGCCAGGCCCATGGCCTGACCCCGGAGCAGGTGGTGGCCATCGCCAGCAATGGCGGCGGCAAGCAGGCGCTGGAGAGCATTGTTGCCCAGTTATCTCGCCCTGATCCGGCGTTGGCCGCGTTGACCAACGACCACCTCGTCGCCTTGGCCTGCCTCGGCGGACGTCCTGCGCTGGATGCAGTGAAAAAGGGATTGCCGCACGCGCCGGCCTTGATCAAAAGAACCAATCGCCGTATTCCCGAACGCACATCCCATCGCGTTGCCGACCACGCGCAAGTGGTTCGCGTGCTGGGTTTTTTCCAGTGCCACTCCCACCCAGCGCAAGCATTTGATGACGCCATGACGCAGTTCGGGATGAGCAGGCACGGGTTGTTACAGCTCTTTCGCAGAGTGGGCGTCACCGAACTCGAAGCCCGCAGTGGAACGCTCCCCCCAGCCTCGCAGCGTTGGGACCGTATCCTCCAGGCATCAGGGATGAAAAGGGCCAAACCGTCCCCTACTTCAACTCAAACGCCGGATCAGGCGTCTTTGCATGCATTCGCCGATTCGCTGGAGCGTGACCTTGATGCGCCTAGCCCAATGCACGAGGGAGATCAGACGCGGGCAAGCAGCCGTAAACGGTCCCGATCGGATCGTGCTGTCACCGGTCCCTCCGCACAGCAATCGTTCGAGGTGCGCGTTCCCGAACAGCGCGATGCGCTGCATTTGCCCCTCAGTTGGAGGGTAAAACGCCCGCGTACCAGTATCGGGGGCGGCCTCCCGGATCCTGGTACGCCCACGGCTGCCGACCTGGCAGCGTCCAGCACCGTGATGCGGGAACAAGATGAGGACCCCTTCGCAGGGGCAGCGGATGATTTCCCGGCATTCAACGAAGAGGAGCTCGCATGGTTGATGGAGCTATTGCCTCAGTGAAGCTT

**RVD1 = codon usage**

HD = CAC GAT

NS = AAT AGC

**Amino acid sequence dARTrep18-3(RVD1=XX)**

MGLINGEQKLISEEDLNGEQKLISEEDLNGLDGEQKLISEEDLNGEQKLISEEDLNGSHMDPIRSRTPSPARELLPGPQPDGVQPTADRGVSPPAGGPLDGLPARRTMSRTRLPSPPAPSPAFSAGSFSDLLRQFDPSLFNTSLFDSLPPFGAHHTEAATGEWDEVQSGLRAADAPPPTMRVAVTAARPPRAKPAPRRRAAQPSDASPAAQVDLRTLGYSQQQQEKIKPKVRSTVAQHHEALVGHGFTHAHIVALSQHPAALGTVAVKYQDMIAALPEA

-1 THEAIVGVGKQWSGARALEALLTVAGELRGPPLQ

00 LDTGQLLKIAKRGGVTAVEAVHAWRNALTGAPLN

01 LTPEQVVAIASXXGGKQALETVQRLLPVLCQAHG

02 LTPEQVVAIASHDGGKQALETVQRLLPVLCQAHG

03 LTPEQVVAIASNIGGKQALETVQRLLPVLCQAHG

04 LTPEQVVAIASHDGGKQALETVQRLLPVLCQAHG

05 LTPEQVVAIASHDGGKQALETVQRLLPVLCQAHG

06 LTPEQVVAIASHDGGKQALETVQRLLPVLCQAHG

07 LTPEQVVAIASNSGGKQALETVQRLLPVLCQAHG

08 LTPEQVVAIASNSGGKQALETVQRLLPVLCQAHG

09 LTPEQVVAIASNSGGKQALETVQRLLPVLCQAHG

10 LTPEQVVAIASHDGGKQALETVQRLLPVLCQAHG

11 LTPEQVVAIASNIGGKQALETVQRLLPVLCQAHG

12 LTPEQVVAIASNGGGKQALETVQRLLPVLCQAHG

13 LTPEQVVAIASNGGGKQALETVQRLLPVLCQAHG

14 LTPEQVVAIASNGGGKQALETVQRLLPVLCQAHG

15 LTPEQVVAIASHDGGKQALETVQRLLPVLCQAHG

16 LTPEQVVAIASNGGGKQALETVQRLLPVLCQAHG

17 LTPEQVVAIASNGGGKQALETVQRLLPVLCQAHG

18 LTPEQVVAIASNGGGKQALESIVA

QLSRPDPALAALTNDHLVALACLGGRPALDAVKKGLPHAPALIKRTNRRIPERTSHRVADHAQVVRVLGFFQCHSHPAQAFDDAMTQFGMSRHGLLQLFRRVGVTELEARSGTLPPASQRWDRILQASGMKRAKPSPTSTQTPDQASLHAFADSLERDLDAPSPMHEGDQTRASSRKRSRSDRAVTGPSAQQSFEVRVPEQRDALHLPLSWRVKRPRTSIGGGLPDPGTPTAADLAASSTVMREQDEDPFAGAADDFPAFNEEELAWLMELLPQ*

**Table S1 DNA sequence of oligonucleotides used in this study**

**Number Designation DNA sequence**

TS 1 Mut-Pos232_R/TS Pho-CTGTTTGCCGACGCCAACG

TS 2 Mut-W232R_F/TS CGTTCCGGCGCACGCGCTC

TS 3 Mut-W232N_F/TS AATTCCGGCGCACGCGCTC

TS 4 Mut-W232C_F/TS TGCTCCGGCGCACGCGCTC

TS 5 Mut-W232H_F/TS CATTCCGGCGCACGCGCTC

TS 6 Mut-W232F_F/TS TTCTCCGGCGCACGCGCTC

TS 7 Mut-W232P_F/TS CCGTCCGGCGCACGCGCTC

TS 8 Mut-W232Y_F/TS TACTCCGGCGCACGCGCTC

TS 9 Mut-W232S_F/TS TCCTCCGGCGCACGCGCTC

TS 10 Mut-W232G_F/TS GGTTCCGGCGCACGCGCTC

TS 11 Mut-W232K_F/TS AAGTCCGGCGCACGCGCTC

TS 12 Mut-W232E_F/TS GAGTCCGGCGCACGCGCTC

TS 13 Mut-W232D_F/TS GACTCCGGCGCACGCGCTC

TS 14 Mut-W232Q_F/TS CAGTCCGGCGCACGCGCTC

TS 15 Mut-W232L_F/TS CTGTCCGGCGCACGCGCTC

TS 16 Mut-W232A_F/TS GCATCCGGCGCACGCGCTC

TS 17 Mut-W232V_F/TS GTGTCCGGCGCACGCGCTC

TS 18 Mut-W232I_F/TS ATCTCCGGCGCACGCGCTC

TS 19 R236G_F/TS CCGGCGCAGGCGCTCTGG

TS 20 R236G_R/TS CCAGAGCGCCTGCGCCGG

TS 21 R266G_F/TS GATTGCAAAAGGTGGCGGCGTG

TS 22 R266G_R/TS CACGCCGCCACCTTTTGCAATC

TS 23 pENTR-CACC_R-pho/TS Pho-GGTGAAGGGGGCGGCCGC

TS 24 U20-Bs4_iniG_F/TS **GA**TATAAACCTGACCCT**T**TTTCTTTCTTG

TS 25 U20-Bs4_iniC_F/TS **CA**TATAAACCTGACCCT**T**TTTCTTTCTTG

TS 26 U20-Bs4_iniA_F/TS **AA**TATAAACCTGACCCT**T**TTTCTTTCTTG

TS 27 EBE-300-iniT-F/TS **T**CTATAAACCTGACCCTCTTTCTTTCTTG

TS 28 EBE-300-iniA-F/TS **A**CTATAAACCTGACCCTCTTTCTTTCTTG

TS 29 EBE-300-iniC-F/TS **C**CTATAAACCTGACCCTCTTTCTTTCTTG

TS 30 EBE-300-iniG-F/TS **G**CTATAAACCTGACCCTCTTTCTTTCTTG

TS 31 EBE-300A1-iniT-F/TS **TA**TATAAACCTGACCCTCTTTCTTTCTTG

TS 32 EBE-300A1-iniA-F/TS **AA**TATAAACCTGACCCTCTTTCTTTCTTG

TS 33 EBE-300A1-iniC-F/TS **CA**TATAAACCTGACCCTCTTTCTTTCTTG

TS 34 EBE-300A1-iniG-F/TS **GA**TATAAACCTGACCCTCTTTCTTTCTTG

TS 35 EBE-300T1-iniT-F/TS **TT**TATAAACCTGACCCTCTTTCTTTCTTG

TS 36 EBE-300T1-iniA-F/TS **AT**TATAAACCTGACCCTCTTTCTTTCTTG

TS 37 EBE-300T1-iniC-F/TS **CT**TATAAACCTGACCCTCTTTCTTTCTTG

TS 38 EBE-300T1-iniG-F/TS **GT**TATAAACCTGACCCTCTTTCTTTCTTG

TS 39 EBE-300G1-iniT-F/TS **TG**TATAAACCTGACCCTCTTTCTTTCTTG

TS 40 EBE-300G1-iniA-F/TS **AG**TATAAACCTGACCCTCTTTCTTTCTTG

TS 41 EBE-300G1-iniC-F/TS **CG**TATAAACCTGACCCTCTTTCTTTCTTG

TS 42 EBE-300G1-iniG-F/TS **GG**TATAAACCTGACCCTCTTTCTTTCTTG

TS 43 EBE-ArtTAL14-1-A0_F/TS **A**CACACACCATAAGGGCATTTCTTTCTTG

TS 44 EBE-ArtTAL14-1-C0_F/TS **C**CACACACCATAAGGGCATTTCTTTCTTG

TS 45 EBE-ArtTAL14-1-G0_F/TS **G**CACACACCATAAGGGCATTTCTTTCTTG

TS 46 EBE-ArtTAL14-2-A0_F/TS **A**CATGTGTGCCTTTTCATTTTCTTTCTTG

TS 47 EBE-ArtTAL14-2-C0_F/TS **C**CATGTGTGCCTTTTCATTTTCTTTCTTG

TS 48 EBE-ArtTAL14-2-G0_F/TS **G**CATGTGTGCCTTTTCATTTTCTTTCTTG
